# Leaf beetles employ tryptophan to detoxify the chemical defenses of poplar trees

**DOI:** 10.1073/pnas.2518096122

**Published:** 2025-12-15

**Authors:** Xingrong Peng, Michael Reichelt, Ana Patricia Baños-Quintana, Beate Rothe, Felix Feistel, Martin Kaltenpoth, Jonathan Gershenzon, Sybille B. Unsicker

**Affiliations:** ^a^Department of Biochemistry, Max Planck Institute for Chemical Ecology, Jena 07745, Germany; ^b^State Key Laboratory of Phytochemistry and Natural Medicines, Kunming Institute of Botany, Chinese Academy of Sciences, Kunming 650201, China; ^c^Department of Insect Symbiosis, Max Planck Institute for Chemical Ecology, Jena 07745, Germany; ^d^Department of Plant-Environment-Interactions, Institute of Botany, Kiel University, Kiel 24118, Germany

**Keywords:** *Populus nigra*, *Chrysomela tremulae*, salicinoids, tryptophan, detoxification

## Abstract

Herbivorous insects have evolved many fascinating adaptations to overcome the chemical defenses of their host plants. This study employed targeted and untargeted metabolomics, coupled with isotope labeling, to shed light on the metabolism of salicinoids—potent antiherbivore phenolic defenses present in the Salicaceae family—in the poplar-specialized leaf beetle, *Chrysomela tremulae*. *C. tremulae* was found to produce a range of metabolites from salicortin and utilize the essential amino acid tryptophan and its breakdown products, namely kynurenine, kynurenic acid, and 4-hydroxyquinoline, to form novel conjugates with the salicinoid metabolite saligenin, which are then excreted in the feces of the beetles. Saligenin and its conjugates are not toxic to *C. tremulae* and similar metabolic pathways were found in other poplar herbivores. Experimental analyses of the gut microbiota revealed that there is no microbial contribution to the formation of tryptophan metabolite–saligenin conjugates. The production of such substances by insect herbivores may be a critical adaptation that enables specialists to survive on a diet high in salicinoid defense compounds. Therefore, identifying the underlying detoxification mechanisms creates opportunities to develop targeted anti-insect agents for protecting salicaceous trees.

## Significance

Specialist insect herbivores feed on plant organs containing high concentrations of chemical defenses without any apparent negative effects. A striking example is the poplar leaf beetle (*Chrysomela tremulae*) which feeds on young poplar leaves that can contain salicinoid defenses at concentrations up to 15%. Here, we show that these leaf beetles efficiently metabolize salicinoids by conjugating them with plant-derived tryptophan metabolites, thereby neutralizing these toxins. This intrinsic detoxification pathway, which functions independently of bacterial symbionts, is an example of a sophisticated biochemical adaptation. This pathway enables *C. tremulae* and related herbivores to exploit heavily defended host plants and could provide novel targets for developing anti-insect agents to protect poplar trees.

Herbivorous insects and their host plants represent the most species-rich groups of living organisms on Earth. Their extraordinary diversity is hypothesized to have arisen from antagonistic coevolution between the two wherein defense and counterdefense reciprocally drove diversification (1–3). The main chemical defenses of plants consist of a series of natural products (2), including terpenoids (4, 5), phenolics (6, 7), *N*-containing compounds (8, 9), and sulfur-containing compounds (10–12). According to optimal defense theory, plants enhance fitness by concentrating these defensive metabolites in tissues critical for survival and reproduction (13). In response, herbivorous insects have evolved diverse mechanisms of resistance, such as behavioral avoidance of toxins, target-site alteration, detoxification, and rapid excretion to facilitate their growth and development on chemically defended plants (14–18). Some herbivores possess specific gut microorganisms that contribute to resistance against plant toxins and pesticides by degrading such chemical compounds (16, 17, 19–22).

**Fig. 1.**
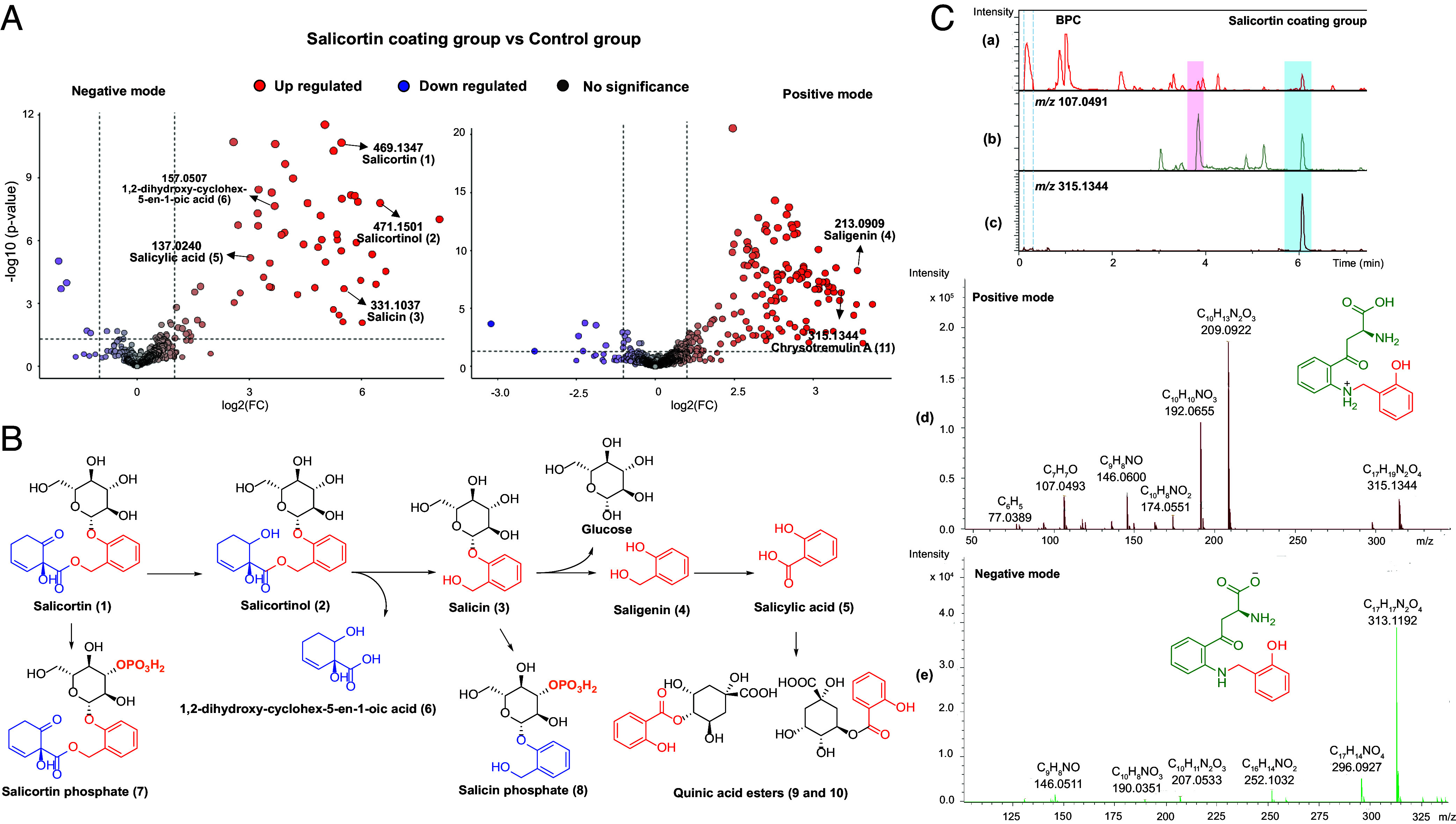
Metabolic analysis of salicortin in the feces of *Chrysomela tremulae*. (*A*) LC-ESI-Q-TOF-MS analysis: Volcano plot analysis of feces metabolites comparing salicortin-coated group with a control group using negative and positive ionization (*P* < 0.05). (*B*) Proposed metabolism of salicortin in *C. tremulae*. (*C*) Detection of chrysotremulin A (**11**) in the feces extract of *C. tremulae*. (*a*) Base peak chromatogram (positive mode) of *C. tremulae* feces in the salicortin-coated group with *Salix viminalis*; (*b*) Extracted ion chromatogram for fragment *m/z* 107.0490 (positive mode); (*c*) Extracted ion chromatogram for molecular ion, *m/z* 315.1344 (positive mode); (*d* and *e*) Positive and negative mode MS/MS fragmentation spectra.

*Populus nigra* L. (Salicaceae) is an economically and ecologically important tree species in many European countries (23) and contains numerous phenolic metabolites, of which the most abundant are proanthocyanidins, hydroxycinnamate esters, and salicinoids (a class of phenolic glycosides) (24–26). Salicinoids are abundant in leaves of poplar trees, and their concentration can reach over 15% of the leaves′ dry weight (27). Salicortin (Fig. 1*B*) and tremulacin, as two of the main salicinoids, are major antiherbivore defenses due to their unstable and toxic 1-hydroxy-6-oxocyclohex-2-ene-1-carboxylic acid (HCC) moiety (24, 27, 28). Previous studies have found that a generalist-feeding herbivore, *Lymantria dispar*, metabolizes salicinoids by conjugating them with glucose, cysteine, or glycine or phosphorylating them. Additionally, a specialist herbivore, *Cerura vinula*, can conjugate salicinoid metabolites with quinic acids (28, 29).

However, many poplar herbivores like *Chrysomela tremulae*, a specialist poplar leaf beetle, preferentially consume the young leaves, where salicinoid concentrations peak (30). This behavioral specialization demonstrates their capacity to effectively metabolize these defense compounds, driving substantial economic losses in *P. nigra* plantations. To deconstruct the underlying biochemical mechanisms, we employ *C. tremulae* as a model system to elucidate salicortin metabolism—tracking the metabolic fate of this dominant *P. nigra* salicinoid through integrated approaches: isotope labeling coupled with targeted/untargeted LC–MS metabolomics, complemented by structural characterization. Complementary bioassays were employed to functionally assess metabolite toxicity/deterrence and microbial contributions to salicinoid processing, thereby identifying novel control targets.

## Results

### Metabolism of Salicortin by *C. tremulae* to Saligenin and Other Products.

Salicinoids are the major phenolic antiherbivore defense metabolites in Salicaceae and among them, salicortin is one of the most dominant compounds, especially in *P. nigra* (27). To investigate salicinoid metabolism in the specialized-feeding poplar leaf beetle *C. tremulae*, we first conducted a feeding experiment with salicortin-coated *Salix viminalis* leaves, that naturally do not contain salicinoids, but *Salix* is closely related to *Populus*. Uncoated *S. viminalis* leaves served as the control. According to the volcano plot analysis (Fig. 1*A* and *SI Appendix*, Fig. S1.3), comparative metabolomic profiling (LC-ESI-Q-TOF-MS analysis) of beetle feces between the control and salicortin-coated group revealed a marked increase in salicortin (**1**), salicortinol (**2**), salicin (**3**), saligenin (**4**), salicylic acid (**5**), and 1,2-dihydroxycyclohex-5-en-1-oic acid (**6**) abundance, which were present exclusively after feeding on salicortin-coated leaves. Detailed MS/MS spectra are shown in *SI Appendix,* Figs. S1.4–S1.9. Fig. 1*B* presents the proposed metabolic pathway for salicortin.

Prior work established that generalist *Lymantria dispar* caterpillars detoxify salicortin via phosphorylation, glycosylation, and glutathione-derived adducts (salicin phosphate, catechol glucosides, N-acetylcysteine catechol) (28). In contrast, the specialist *Cerura vinula* hydrolyzes salicortin to saligenin and HCH, with subsequent oxidation to salicylic acid and esterification with quinic acid (29). In this study, we also tentatively identified salicortin phosphate (**7**), salicin phosphate (**8**), and two salicyloyl quinic acids (**9** and **10**) (*SI Appendix,* Figs. S1.10 and S1.11) in the feces extract of *C. tremulae* larvea fed on *S. viminalis* leaves coated with salicortin, which illustrated that this specialist leaf beetle is also able to metabolize salicortin by conjugating with polar molecules (Fig. 1*B*).

### Saligenin Is Conjugated to Tryptophan Derivatives.

It was suggested earlier that salicinoids can be activated by degradation to the toxic metabolite saligenin or catechol, which may be oxidized to form highly reactive quinones (31, 32). Thus, we investigated the metabolism of saligenin by *C. tremulae* using LC-ESI-HRMS analysis. The detailed analysis of full scan MS spectra of saligenin in positive mode revealed a characteristic in-source fragment (*m/z* 107.0490 [M – H_2_O + H]^+^) (Fig. 1*C* and *SI Appendix,* Fig. S1.7). Two peaks with this diagnostic feature were identified showing significant differences between insects feeding on salicortin-coated leaves compared to the uncoated control group (Fig. 1*C*). One peak was saligenin itself (t*_R_* = 3.8 min, *m/z* 107.0490 [M – H_2_O + H]^+^, *m/z* 213.0909 [2M – 2H_2_O + H]^+^ for C_14_H_13_O_2_). The second peak was compound **11** (t*_R_* = 6.1 min, *m/z* 315.1344 [M + H]^+^ for C_17_H_19_N_2_O_4_) (Fig. 1*C*), deduced as a kynurenine conjugate of saligenin by its MS/MS fragmentation in the positive and negative ionization modes (*SI Appendix*, Figs. S2.1, S2.4 and S2.5).

**Fig. 2.**
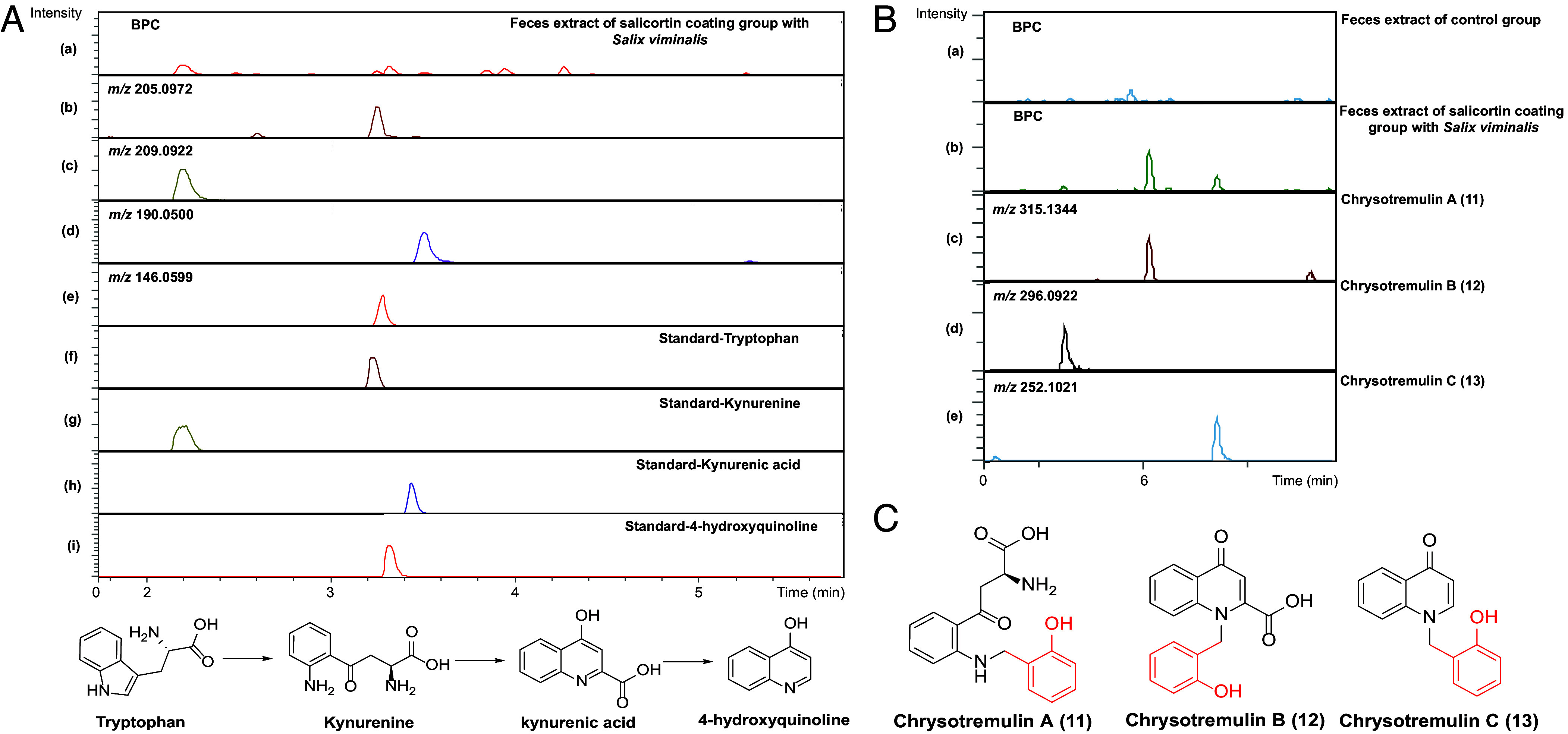
(*A*) Identification of kynurenine pathway metabolites in the fecal extract of the salicortin-coated group with *Salix viminalis* was accomplished by comparison with authentic reference standards utilizing LC-ESI-HRMS. (*a*) Base peak chromatogram in positive ionization mode of the feces extracts in the salicortin-coated group; (*b* to *e*) Extracted ion chromatograms for tryptophan (*m/z* 205.0972), kynurenine (*m/z* 209.0922), kynurenic acid (*m/z* 190.0500), and 4-hydroxyquinoline (*m/z* 146.0599); (*f* to *i*) Base peak chromatograms of authentic standards (10 μg/mL) of tryptophan, kynurenine, kynurenic acid, and 4-hydroxyquinoline. (*B*) The presence of conjugates in the feces extract of the salicortin-coated group with *S. viminalis* was detected by LC-ESI-HRMS in positive ionization mode. (*a* and *b*) Base peak chromatogram of feces extracts in the control and salicortin-coated group; (*c* to *e*) Extracted ion chromatograms for chrysotremulin A (**11**, *m/z* 315.1344), chrysotremulin B (**12**, *m/z* 296.0922), and chrysotremulin C (**13**, *m/z* 252.1021) in the feces extracts of the salicortin-coated group. (*C*) Structures of chrysotremulins A–C (**11–13**).

Kynurenine is a degradation product of tryptophan, formed by a well-established pathway in both mammals and insects (33, 34). We found *C. tremulae* feces to contain diagnostic peaks for tryptophan and kynurenine (35). Further degradation products of kynurenine including kynurenic acid and 4-hydroxyquinoline were also observed (35). These data suggest that tryptophan can be converted by *C. tremulae* into kynurenine, kynurenic acid, and 4-hydroxyquinoline in sequence (Fig. 2*A*). The comparison of their retention times and MS fragmentation patterns with those of authentic standards confirmed our hypothesis (Fig. 2*A* and *SI Appendix,* Figs. S2.6–S2.9).

Since *C. tremulae* conjugates kynurenine to saligenin, we investigated whether kynurenic acid and 4-hydroxyquinonline can also be coupled to this salicortin metabolite. Looking more closely at the LC-ESI-HRMS data of feces extracts from beetles that fed on salicortin-coated leaves, we found two other saligenin conjugates, chrysotremulin B (**12**) and chrysotremulin C (**13**). Compound **12** showed a protonated molecular ion at *m/z* 296.0922 [M + H]^+^ (calcd. for C_17_H_14_NO_4_, 296.0917) and another prominent peak at *m/z* 190.0500 [M – C_7_H_7_O + H]^+^ attributable to the diagnostic fragment of kynurenic acid. A parent ion peak at *m/z* 252.1021 [M + H]^+^ (calcd. for C_16_H_14_NO_2_, 252.1019) and a fragment at *m/z* 146.0599 [M – C_7_H_7_O + H]^+^ assignable to a 4-hydroxyquinoline moiety were consistent with the structure of compound **13** (Fig. 2*B* and *SI Appendix*, Fig. S2.10). The structures of chrysotremulin A (**11**), chrysotremulin B (**12**), and chrysotremulin C (**13**) were further confirmed by NMR spectroscopy (*SI Appendix*). The absolute stereochemistry of compound **11**, which possesses a single chiral center, was unequivocally assigned as 9*S*. This configurational assignment was corroborated by a chemical synthesis starting from enantiomerically pure L-kynurenine. The synthetic and natural compound **11** demonstrated identical spectroscopic properties (*SI Appendix*, Figs. S2.41 and S2.42). Critically, the congruence of their matching circular dichroism (CD) spectra (*SI Appendix*, Figs. S2.44–S2.46) and their optical rotation (*SI Appendix*, Figs. S2.47 and S2.48) provided conclusive evidence for the *S*-configuration at the C9 position.

To confirm that all the products identified in *C. tremulae* feces were indeed products of salicortin, *P. nigra* leaves were coated with ^13^C-labeled salicortin, unlabeled salicortin, or water as a control. Compared to the control group, the relative contents of salicortin, its metabolites, and conjugates significantly increased in the unlabeled salicortin group (*SI Appendix*, Figs. S1.13 and S2.11). In addition, as shown in *SI Appendix,* Table S1.1, all peaks of salicortin, its metabolites, and conjugates were labeled, demonstrating that all were derived by the metabolism of salicortin (*SI Appendix,* Figs. S1.14–S2.20 and S2.12).

### The Salicortin Metabolite Saligenin Has No Negative Impact On *C. tremulae*.

One of the major *C. tremulae* metabolites of salicortin was saligenin. Since this simple salicinoid can be toxic to insect herbivores (13), we tested its effect on *C. tremulae* performance. *P. nigra* leaves were coated with saligenin at three concentrations (low: 3.03 mg/mL; medium: 6.06 mg/mL; high: 20.20 mg/mL) with water as a control, and larval performance was monitored from hatching to pupation.

**Fig. 3.**
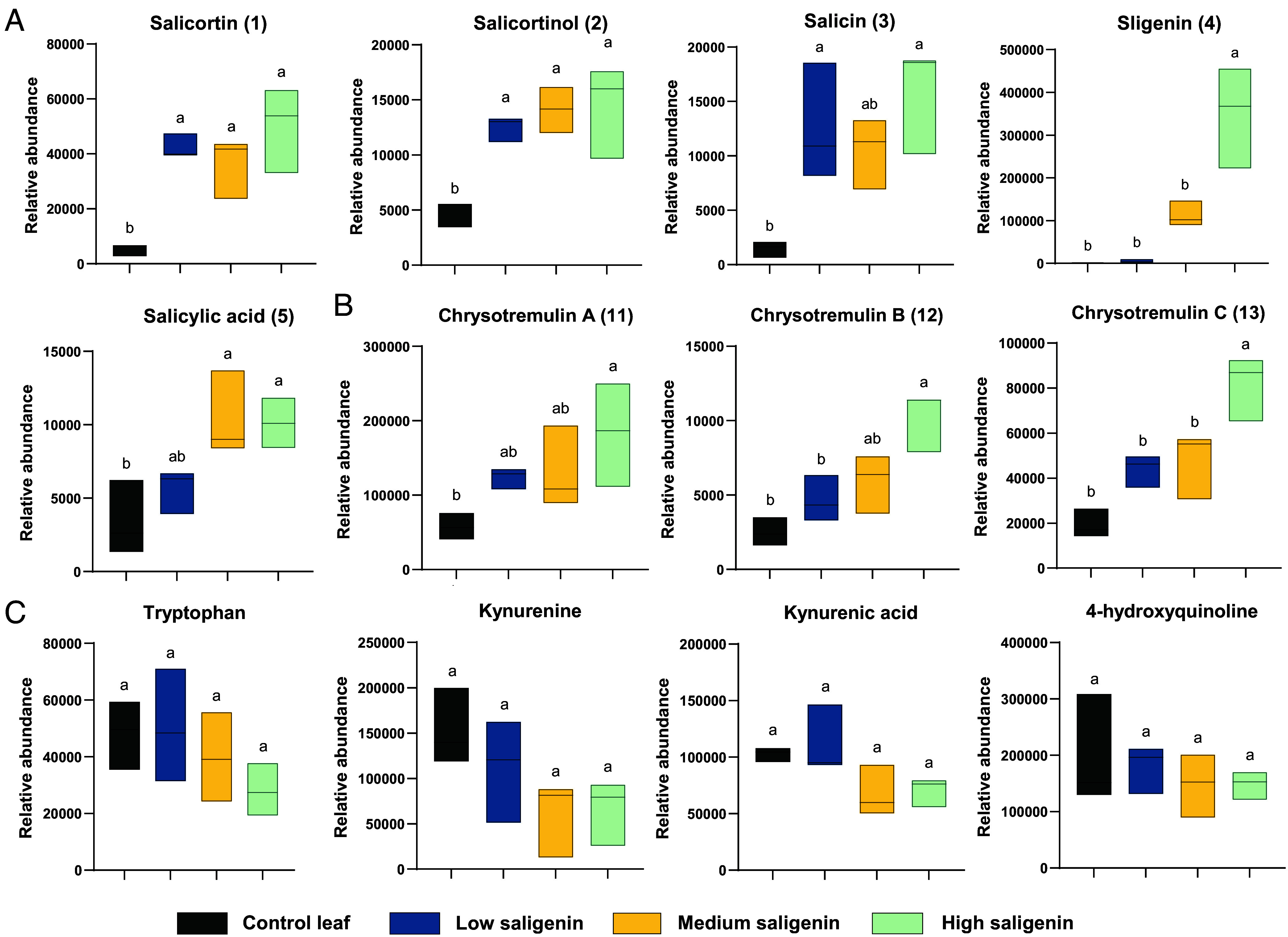
Metabolite profile alterations in *C. tremulae* larval feces following 72 h of feeding on leaves coated with graded saligenin concentrations. (*A*) Change in the relative content of the main salicortin metabolites, salicortin (**1**), salicortinol (**2**), salicin (**3**), saligenin (**4**), and salicylic acid (**5**). (*B*) Change in the relative content of chrysotremulins A–C (**11–13**). (*C*) Change in the relative content of tryptophan metabolites, including tryptophan, kynurenine, kynurenic acid, and 4-hydroxyqunoline. Data were acquired as full scan peak intensities of extracted ion traces on an LC-Q-TOF-MS. Four treatment groups: control leaf (natural salicinoid concentrations), low saligenin (adding 3.03 mg/mL saligenin water solution), medium saligenin (adding 6.06 mg/mL saligenin water solution), and high saligenin (adding 20.2 mg/mL saligenin water solution). Each group had three Petri-dish replicates (with 10 larvae in each Petri-dish). Results shown represent the means ± SE. Different letters indicate significant differences (*P* < 0.05, one-way ANOVA with Tukey’s post hoc test) (n = 30).

Our results showed that saligenin supplementation had no significant effects on the growth parameters of the larvae (e.g., pupation rate, developmental duration) or foliar consumption (leaf area loss) (*SI Appendix,* Figs. S3.1 and S3.2), indicating that saligenin does not function as a toxin or feeding deterrent in *C. tremulae* larvae. However, added saligenin increased the accumulation of salicortin and its metabolites, suggesting saligenin-induced suppression of salicortin degradation (Fig. 3*A*). Concurrent increases in phosphorylated derivatives (salicortin phosphate, salicin phosphate) and salicyloyl quinic acids further implied a limited capacity at the hydrolytic or conjugative steps of salicortin metabolism. Elevated levels of chrysotremulins A–C (**11–13**) (Fig. 3*B*), correlated with higher saligenin doses, highlighting the role of the kynurenine pathway derivatives in detoxifying saligenin. However, increasing saligenin concentrations did not specifically alter the amounts of tryptophan, kynurenine, kynurenic acid, and 4-hydroxyquinoline pools (Fig. 3*C*), suggesting potential suppression of alternative tryptophan metabolic pathways.

### Role of the Gut Microbiota in the Metabolism of Saligenin and Chrysotremulins.

Tryptophan is an essential aromatic amino acid that cannot be synthesized by insects (36) but must be acquired from the diet or from microbial symbionts. Insect–bacteria symbioses where the microbial partner supplies essential amino acids to their hosts are well documented among certain lineages of Coleopterans (37). As tryptophan derivatives are employed to conjugate with saligenin to form the chrysotremulins A–C (**11–13**), we hypothesized that the beetle’s microbiome may be involved in supplying this amino acid needed for chrysotremulin metabolism (27, 38).

We characterized the gut microbiota of *C. tremulae* via 16S rRNA amplicon sequencing, revealing low-diversity communities dominated by persistent taxa (*SI Appendix*, Figs. S4.1–S4.3). One *Lactococcus* and two *Enterobacter* amplicon sequence variants (ASVs) were shared across life stages, while the intracellular symbiont *Symbiodolus* (39) exhibited stage-specific relative abundance (high in adults, low in larvae). Quantitative PCR with general 16S primers confirmed the gut to be the primary bacterial niche, with comparable bacterial titers in both larvae and adults (*SI Appendix,* Fig. S4.4).

To assess microbial involvement in chrysotremulin synthesis, we conducted the saligenin feeding bioassay (20.20 mg/mL vs. water control) using microbiota-depleted (i.e., egg surface-sterilized; *SI Appendix,* Fig. S4.5), reinfected, and control larvae. Neither saligenin exposure nor microbiota manipulation affected larval development (*SI Appendix*, Figs. S4.6–S4.8). Key metabolic shifts included the elevation of kynurenine concentration in larval tissues (*SI Appendix*, Figs. S4.9 and S4.10), and an increase of the fecal saligenin in the high-saligenin diet treatment, as well as higher salicylic acid and kynurenine concentrations in the feces of the control diet treatment (*SI Appendix*, Figs. S4.11 and S4.12). There were no significant changes in salicinoid or tryptophan metabolites explained by the microbiota alteration (*SI Appendix*, Tables S4.4 and S4.5). Interestingly, chrysotremulins A (**11**) and C (**13**) were detected in defensive gland secretions independently of diet and microbiota treatments (*SI Appendix,* Fig. S4.13), implying that these conjugates may be involved in the beetle’s antipredator defense.

Further 16S rRNA amplicon sequencing analysis of the larvae identified *Symbiodolus* as the dominant ASV across all treatments (*SI Appendix,* Fig. S4.14). *Symbiodolus* is likely incapable of producing tryptophan, since the available genomes of 16 strains from different insect taxa encode the shikimate pathway until chorismate, but not the subsequent steps leading to the biosynthesis of tryptophan (39). In silico exclusion of *Symbiodolus* sequences from our 16S dataset confirmed the effective removal of the gut symbiont *Enterobacter* in microbiota-depleted groups, as well as its reacquisition in reinfected larvae (*SI Appendix*, Figs. S4.15 and S4.16). *Enterobacter*’s absence in microbiota-depleted larvae and poplar leaves, coupled with its presence in egg coatings, suggests the vertical transmission via the egg surface—a known Coleopteran symbiont transmission strategy (37).

We applied Koch’s postulates to assess whether the gut microbiota mediates chrysotremulins formation (38). Taken together, our results indicate that the gut bacteria of *C. tremulae* do not play a critical role in this process. While microbially mediated metabolism of plant defense compounds has been shown in other beetles (19), herbivorous insects have often adapted to toxic diets without the aid of microbial partners (40), which seems to be the case in *C. tremulae*.

### Similar Pathways of Salicortin Metabolism Also Occur in Other Poplar Herbivores.

To determine whether the pathways of salicinoid metabolism in *C. tremulae* also occur in other poplar herbivores, we conducted the targeted LC–MS/MS profiling of fecal extracts from 23 additional insect species spanning both related and phylogenetically distant lineages. Our data revealed that the conversion of salicortin to salicylic acid was widespread among poplar herbivores, whereas salicortin phosphate was absent in all species (*SI Appendix,* Table S2.2). Most other insects tested showed the presence of at least one salicyloyl quinic acid, but no salicyloyl quinic acids were detected in *Saperda carcharias* (Cerambycidae) and salicortin phosphate, salicortinol phosphate were absent there too (*SI Appendix,* Table S2.2).

Notably, chrysotremulin A (**11**) was detected in the feces extract of five beetles (*Saperda carcharias*, *Chrysomela populi*, *Chrysomela saliceti*, *C. tremulae*, and *Plagiodera versicolora*) and two caterpillars (*Laothoe populi* and *Smerinthus ocellata*). Quantitative analysis indicated that the highest concentration of compound **11** was found in *P. versicolora* (4.36 mg/g) feces, while the lowest was in *S. carcharias* (0.014 mg/g) (*SI Appendix,* Table S2.3) feces.

## Discussion

Insect herbivores are well known to detoxify plant defenses by functionalization and conjugation with sugars or glutathione (28, 29). Here, we describe conjugations of saligenin—a toxic phenolic metabolite—with essential amino acid derivatives. Such metabolic ingenuity may explain why specialist herbivores like *Chrysomela* spp. preferentially target young leaves—tissues with the highest salicinoid concentrations—turning the plant’s optimal defense allocation into a liability. Critically, discovery of this distinct metabolic mechanism pinpoints the kynurenine pathway as an intervention target for the next-generation insect control agents aimed at protecting *P. nigra* plantations.

The lack of tryptophan depletion despite elevated saligenin conjugation (Fig. 3*C*) challenges conventional detoxification cost paradigms. We propose that saligenin may inhibit competing tryptophan metabolic routes (35), redirecting flux toward kynurenine production.

The detection of chrysotremulin A (**11**) in distantly related poplar herbivores (*Laothoe populi*, *Saperda carcharias*; *SI Appendix,* Table S2.2) indicates convergent evolution of tryptophan-assisted detoxification. Both Coleopteran (*Saperda carcharias*) and Lepidopteran (*Laothoe populi*) herbivores were separated by over 300 million years of evolution, which suggests independent origins of this metabolic trait (41).

In summary, our multiomics study reveals *that C. tremulae* employs a salicinoid detoxification pathway: conjugation of saligenin with dietary tryptophan derivatives via the kynurenine pathway. These conjugates—distinct from known phosphorylated/quinic acid metabolites—enable toxin neutralization without microbial assistance. While saligenin lacks toxicity or deterrence to *C. tremulae*, its rapid conjugation may incur the metabolic costs due to its usage of tryptophan. This intrinsic mechanism is conserved in herbivores adapted to salicinoids and identifies the kynurenine metabolic pathway in pest insects as a target to protect poplar trees from herbivory.

## Materials and Methods

### Plant Materials and Insects.

Black poplar (*P. nigra*) leaves were collected from trees growing in the greenhouse as described in Fabisch et al. 2019 (27). The light period was set from 6:30 to 20:30 (14 h), while temperatures were kept between 21 and 23 °C during the day and between 19 and 21 °C at night. The humidity was regulated between 50 to 60%. *P. nigra* is a natural host plant of the poplar leaf beetle *C. tremulae*. The light period was set from 6:30 to 20:30 (14 h), while temperatures were kept between 21 and 23 °C during the day and between 19 and 21 °C at night. The humidity was regulated between 50 to 60%. *C. tremulae* leaf beetles were derived from a laboratory rearing at the MPI-CE (Jena, Germany) and were hatched from eggs, and then reared on *P. nigra* leaves in the laboratory.

### *Salix viminalis* Leaf Coating Experiment With Salicortin.

*Salix viminalis* leaves derived from one tree individual at the MPI-CE in Jena were cut off from the tree and individually placed in 2 mL Eppendorf tubes, filled with tap water and sealed with Parafilm to avoid leaf desiccation. A solution of salicortin and water was pipetted onto the leaves as depicted in *SI Appendix,* Fig. S1.1. Each treatment had ten repeats. Individual leaves were placed in a petri dish together with one adult *C. tremulae* leaf beetle. After the beetles had consumed the entire leaf, feces from each individual beetle were collected and air-dried before they were extracted with 50% MeOH/H_2_O for chemical analysis.

### Extract and Analysis of Beetle Feces.

First, 20 mg feces were extracted with 50% MeOH/H_2_O (1 mL) by homogenization in a Minilys cell disruptor (2 mL tubes, 60 s and 4,000 rpm), using 1.4 mm o.d. ZrO_2_ beads. Then, 2 μL supernatants from each sample were subjected to ultra-high-performance liquid chromatography–electrospray ionization–quadrupole time-of-flight mass spectrometry system (UHPLC-ESI-Q-ToF-MS, Ultimate 3000 series RSLC; Thermo Dionex, MA, USA coupled to a Bruker tims-TOF mass spectrometer, Bremen, Germany) analysis according to the methods in supporting information. Data were analyzed using the MetaboScape 2023b software (Bruker, Bremen, Germany) and MetaboAnalyst 5.0 (https://dev.metaboanalyst.ca/) (details in *SI Appendix*).

### ^13^C *P. nigra* Labeling and Isolation.

Stable isotope labeling of young *P. nigra* trees was achieved in a growth chamber resembling a setup described previously (29). The ^13^CO_2_-labeling of young *P. nigra* trees was stopped at day 28, and newly grown plant tissue (leaves with petioles) was collected and lyophilized, yielding 10 g dry material. The dry samples containing stainless steel balls were crushed using a paint shaker (Skandex SO-10M, Fluid Management Europe, The Netherlands) and divided into four tubes (50 mL). Then, 45 mL 100% MeOH was added into each tube, and ultrasound was used to extract samples three times. The detailed isolation procedure is shown in *SI Appendix*.

### *P. nigra* Leaf Coating Experiment with ^13^C-Salicortin.

Freshly cut leaves of *P. nigra* were used for feeding experiments. An aqueous solution of [U-^13^C] salicortin (25 mg/mL), an unlabeled salicortin aqueous solution (25 mg/mL), and water were used to separately coat leaves, each treatment had three repeats. The specific method is shown in *SI Appendix*, Fig. S1.1. Feces were collected and analyzed using UHPLC-ESI-Q-ToF-MS analysis.

### The Extraction, Isolation, and Identification of Chrysotremulins A–C (1–3) from *C. tremulae* Feces.

*C. tremulae* larvae were hatched from eggs and reared on *P. nigra* leaves in the laboratory. A total of 43.6 g air-dried feces of adult *C. tremulea* beetles was used for the extraction and isolation (supporting information-2). NMR spectra for the structure elucidation of chrysotremulins A–C (**1**–**3**) were recorded on a Bruker AVANCE NEO 400 MHz, 500 MHz, and Bruker AVANCE III HD 700 MHz spectrometer, equipped with a 1.7 mm TCI micro-cryo probe (Bruker Biospin, Rheinstetten, Germany) using NMR tubes of 1.7 mm outer diameter. NMR spectroscopic data of synthetic mediates and compound **1** were collected on a Bruker AV-600 MHz (Bruker, Zurich, Switzerland), and tetramethyl chlorosilane (TMS) was as internal standard for chemical shifts (details in *SI Appendix*).

### *P. nigra* Leaves Coating Experiment With Saligenin.

In this bioassay experiment, four groups including control, low concentration, medium concentration, and high concentration groups were set. The natural concentration of saligenin in poplar leaves was calculated as 6.45 mg/g (fresh weight) based on the assumption of salicin, salicortin, homaloside D, and salicortin-6-benzoate being fully converted into saligenin using published concentrations of those salicinoids in *P. nigra* leaves (27). One leaf was about 1.25 g, and the natural content of saligenin was 8.06 mg for one leaf. The low concentration, medium concentration, and high concentration were prepared as 3.02, 6.06, and 20.2 mg/mL (saligenin/water, m/v; Merck, Germany), and then 400 μL of this solution (saligenin in water) was added, respectively, thus mimicking 0.15, 0.3, and 1.0-fold natural content of saligenin equivalents. The specific experiment is shown in *SI Appendix*.

### Saligenin Feeding Assay With Microbiota-depleted Reinfected and Control *C. tremulae* Larvae.

To assess the role of the gut microbiota of *C. tremulae* in the saligenin metabolism, we randomly assigned 300 eggs from different egg clutches to the following microbial treatments: microbiota-depleted (surface-sterilized eggs), control (unaltered native microbiota on the egg surface), and reinfected (surface-sterilized eggs which were reinoculated with the PBS suspension of the first washing step). Details are shown in *SI Appendix*.

## Supplementary Material

Appendix 01 (PDF)

## Data Availability

Metabolomics data are available from the Edmond (42). The raw data from the amplicon sequencing studies were deposited in GenBank under the BioProject number: PRJNA1365733 (43). All other data are included in the article and/or *SI Appendix*.
