# Supplementary material for: Leaf beetles employ tryptophan to detoxify the chemical defenses of poplar trees
Source: Proc Natl Acad Sci U S A. 2025 Dec 15;122(51):e2518096122. doi: 10.1073/pnas.2518096122 (PMC12745818; doi:10.1073/pnas.2518096122)
Supplement: Supplementary file 1 — Appendix 01 (PDF) [file pnas.2518096122.sapp.pdf]

PNAS

Supporting Information for

Leaf beetles employ tryptophan to detoxify the chemical defenses of poplar trees

Xingrong Peng[a,b], Michael Reichelt[a], Ana Patricia Baños-Quintana[a,c], Beate Rothe[a], Felix Feistel[a], Martin Kaltenpoth[c], Jonathan Gershenzon[a], and Sybille B. Unsicker[a,d]

[a]Department of Biochemistry, Max Planck Institute for Chemical Ecology, Hans-Knöll Str. 8, 07745 Jena, Germany; [b]Key Laboratory of Phytochemistry and Natural Medicines, Kunming Institute of Botany, Chinese Academy of Sciences, Kunming 650201, China; [c]Department of Insect Symbiosis, Max Planck Institute for Chemical Ecology, Hans-Knöll Str. 8, 07745 Jena, Germany; [d]Plant-Environment-Interactions Group, Institute of Botany, Kiel University, Am Botanischen Garten 5, 24118 Kiel, Germany

*Xingrong Peng; Sybille B. Unsicker

Email: pengxingrong@mail.kib.ac.cn; sunsicker@bot.uni-kiel.de

This PDF file includes:

Supporting text

SI-1: Materials and Methods, Figures S1.1 to S1.22, Tables S1.1

SI-2: Materials and Methods, Figures S2.1 to S2.48, Tables S2.1 to S2.3

SI-3: Materials and Methods, Figures S3.1 to S3.2

SI-4: Materials and Methods, Figures S4.1 to S4.16, Tables S4.1 to S4.5

SI References

SI-1: The metabolism of salicortin in *Chrysomela tremulae*

Materials and Methods

1. Plant materials and insects

Black poplar (*Populus nigra*) leaves were collected from trees growing in the greenhouse as described in Fabisch et al. 2019 (1). The light period was set from 6:30 to 20:30 (14 h), while temperatures were kept between 21 and 23 °C during the day and between 19 and 21°C at night. The humidity was regulated between 50 to 60%. The light period was set from 6:30 to 20:30 (14 h), while temperatures were kept between 21 and 23 °C during the day and between 19 and 21°C at night. The humidity was regulated between 50 to 60%. *P. nigra* is a natural host plant of the poplar leaf beetle *Chrysomela tremulae*. *C. tremulae* leaf beetles were derived from a laboratory rearing at the MPI-CE (Jena, Germany) and were hatched from eggs and reared on *P. nigra* leaves in the laboratory.

2. Salicortin coating experiment with *Salix viminalis*

Salix viminalis leaves derived from one tree individual at the MPI-CE in Jena were cut off from the tree and individually placed in 2 mL Eppendorf tubes, filled with tap water and sealed with Parafilm to avoid leaf desiccation. A solution of salicortin and water was pipetted onto the leaves as depicted in **Figure S1.1**. Individual leaves were placed in a petri dish together with one adult *C. tremulae* leaf beetle. After the beetles had consumed the entire leaf, feces from each individual beetle were collected and air dried before they were extracted with 50% MeOH/H_2O for chemical analysis.

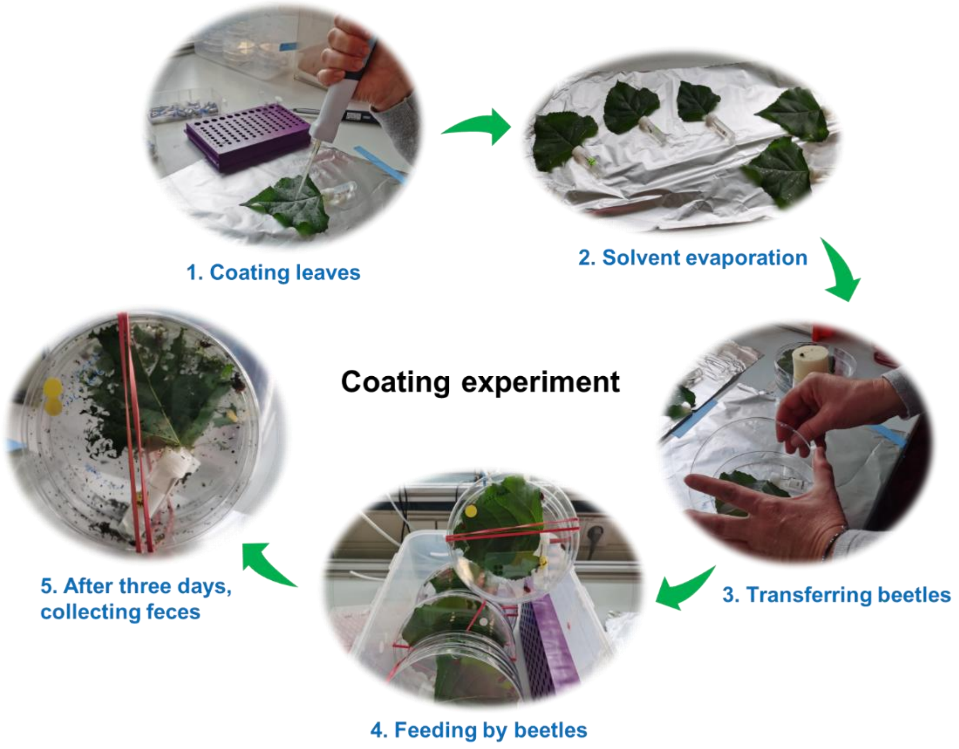

Figure S1.1 The procedure of coating experiment.

3. Extract and analysis of beetle feces

20 mg feces were extracted with 50% MeOH/H_2O (1 mL) by homogenization in a Minilys cell disruptor (2 mL tubes, 60 s and 4000 rpm), using 1.4 mm o.d. ZrO_2 beads. 100 µL supernatants from each sample were subjected to the ultra-high-performance liquid chromatography–electrospray ionization–quadrupole time-of-flight mass spectrometry system (UHPLC-ESI-Q-ToF-MS, Ultimate 3000 series RSLC, Thermo Dionex, MA, USA coupled to a Bruker tims-TOF mass spectrometer, Bremen, Germany) analysis. An aliquot of 2 µL was subjected to UHPLC-ESI-Q-ToF-MS. The UHPLC was equipped with a C18 reverse phase column (ZORBAX Eclipse XDB-C18, 1.8 µm, 2.1 × 100 mm, Agilent Technologies, Boblingen, Germany) maintained at 25 ºC and operated at 0.3 mL/min with a gradient flow of 0.1% aqueous formic acid (solvent A) and acetonitrile (solvent B) with the following profile: 5% B from 0-0.5 min, 5-60% B from 0.5 to 11 min, 60-100% B from 11-11.1 min, and kept at 100% B until 12 min, then re-equilibrated at 5% B from 12.1 to 15 min. HRMS analyses were performed separately with positive and negative ionization mode and automatic MS2 scans (“autoMS”) enabled. The source end plate offset was kept at 500 V and the capillary voltage at 4500 V, with the nebulizer gas at 2.8 bar, dry gas at 8 L/min and the drying temperature at 280 ºC. Ion transfer was performed with a funnel 1 RF of 150 Vpp, funnel 2 RF of 200 Vpp, multipole RF of 50 Vpp, and a deflection delta of 70 V, with the quadrupole ion energy maintained at 4 eV (low mass 90 m/z). The mass scan range was 50-1500 *m/z* at an acquisition rate of 12 Hz. Collision energies were stepped in a 50:50 timing between a collision energy of 20 eV and 50 eV, respectively. At the beginning of each chromatographic analysis 10 µL of a sodium format-isopropanol solution (10 mM solution of NaOH in 50/50 (v/v%) isopropanol-water containing 0.2% formic acid) was injected into the dead volume of the sample injection for recalibration of the mass spectrometer using the expected cluster ion m/z values. Data were analyzed using the MetaboScape 2023b software (Bruker, Bremen, Germany) and MetaboAnalyst 5.0 (https://dev.metaboanalyst.ca/). Automated peak picking and alignment were done using MetaboScape 2023b within a retention time between 0.4 and 11 minutes, signal intensity ≥ 1500, maximum deviation of 2 ppm, and minimum occurrence in at least 4 samples. Feature groups, potentially representing single metabolites, were reduced to one bucket by the MetaboScape software to represent the respective metabolite in later analysis.

4. Analysis of salicortin, salicin, saligenin, and salicylic acid in *C. tremulae* feces

For the preparation of the standard solution, 1 mg salicortin (Carl Roth GmbH & Co. KG; 334T.2; Lot 364333462), salicin (Sigma-Aldrich; S0625; Lot 23460555), saligenin (2-hydroxybenzyl alcohol; Sigma-Aldrich; 166952; Lot 102609726), and salicylic acid (Sigma-Aldrich; S5922; Lot 075K3737) were put into 1.5 mL tubes, and 1 mL MeOH was added to prepare a 1mg/mL solution. Then, standard solution was diluted to 10 µg/mL, which was subjected to UHPLC-HRMS (Bruker timsTOF mass spectrometer, Bremen, Germany) analysis, using

the chromatographic system described in supporting information-1.3.

5. ^{13}C *P. nigra* labeling

Stable isotope labeling of young *P. nigra* trees was achieved in a growth chamber resembling a setup described previously in Feistel et al. (2018) (2). The greenhouse light system (Philips SON-T Agro 400 W) was used to provide constant light exposure from 6:30 to 22:00 (15.5 h). Temperature and relative humidity were kept between 20°C to 30°C and 50% to 80%, respectively. Six *P. nigra* plants were pruned to a height of about 30 cm, leaving a few basal leaves. After being transferred to the growth chamber, plants were kept in darkness for the first 2 days. At the beginning of day 3, the respired CO_2 (natural abundance isotope ratio) was removed from the chamber′s atmosphere and 450 ppm $^{13}CO_2$ was injected into it. This $^{13}CO_2$ level was kept constant during the entire experimental time (26 days). At the end of each day′s light period, $^{13}CO_2$ injection was stopped and respired CO_2 was continuously removed during the night.

6. ^{13}C-salicortin isolation

The $^{13}CO_2$-labeling of young *P. nigra* trees was stopped at day 28, and newly grown plant tissue (leaves with petioles) was collected and lyophilized yielding 10 g dry material. The dry samples containing stainless steel balls were crushed using a paint shaker (Skandex, SO 10M, Fluid Management Europe, The Netherlands) and divided into four tubes (50 mL). 45 mL 100% MeOH were added into each tube and ultrasound was used to extract samples for three times. The extracts were filtered (paper filters, MN 615 ¼, 125 mm, Macherey-Nagel), pooled and evaporated under reduced pressure using rotary evaporator R-114 (Buchi Labortechnik, Flawil, Switzerland) and a Genevac HT-4X vacuum centrifuge (Genevac, Ipswich, UK), resulting in 5 g dry crude extract. The dry matter (5 g) was dissolved in MeOH (20 mL) and centrifuged (AvantiTM J-20 XP centrifuge with JS-4.3 (Beckman Coulter TM, Brea, USA)]. The supernatant was mixed with 5 g polyamide (for column chromatography, 6; Sigma-Aldrich). After the MeOH was evaporated, the dry mixture was subjected to separation on a column chromatography (44 × 2.5 cm) containing 20 g polyamide, which was eluted by Mili-Q water (500 mL), 5% MeOH/H_2O (500 mL), 10% MeOH/H_2O (750 mL), 15% MeOH/H_2O (1000 mL), and 20% MeOH/H_2O (250 mL). 10% MeOH/H_2O (750 mL) was divided into three parts (10%-1, 10%-2, and 10%-3, each 250 mL) for elution, meanwhile, 15% MeOH/H_2O (1000 mL) was divided into 15%-1, 15%-2, 15%-3, and 15%-4 (each 250 mL) for eluting the column and these fractions were kept in 300 mL glass flasks. Finally, the column was washed using MeOH (500 mL) and acetone (500 mL). The combined MeOH and acetone parts were concentrated and samples were stored in fridge at –20 ºC.

HPLC-MS detection of all fractions was performed on an Agilent 1100 HPLC system, consisting of a degasser, a binary pump G1312A, an autosampler G1313A, a photodiode array detector G1316A (detection 200–6500 nm;

Agilent Technologies, Waldbronn, Germany), and an Esquire 6000 ion trap mass spectrometer (Bruker Daltonik, Bremen, Germany). The HPLC and MS parameters were similar to those of the isolation of conjugates (see part 3).

Based on the results of LC-MS, H_2O fraction contained salicortin and some sugars. Then 500 mL H_2O fraction was subjected to solid-phase extraction on two HR-X SPE columns (each 6 mL, 1000 g sorbent, Macherey-Nagel, Düren, Germany). After loading, columns were washed with H_2O, 5% MeOH/H_2O, 10% MeOH/H_2O, 15% MeOH/H_2O, 20% MeOH/H_2O, 30% MeOH/H_2O and 35% MeOH/H_2O (each 300 mL). Similarly, these fractions were detected by LC-MS (Agilent 1100 system coupled to Esquire 6000 MS as above) and three salicortin-containing fractions (A, B, and C) were obtained based on the purity of salicortin. Fractions A, B, and C were combined and concentrated by reduced pressure to get ^{13}C-salicortin (336.44 mg) (**Figure S1.2**). Non-labeled salicortin (natural abundance ^{13}C, 196.11 mg) was isolated in the same manner from unlabeled *P. nigra* tissue.

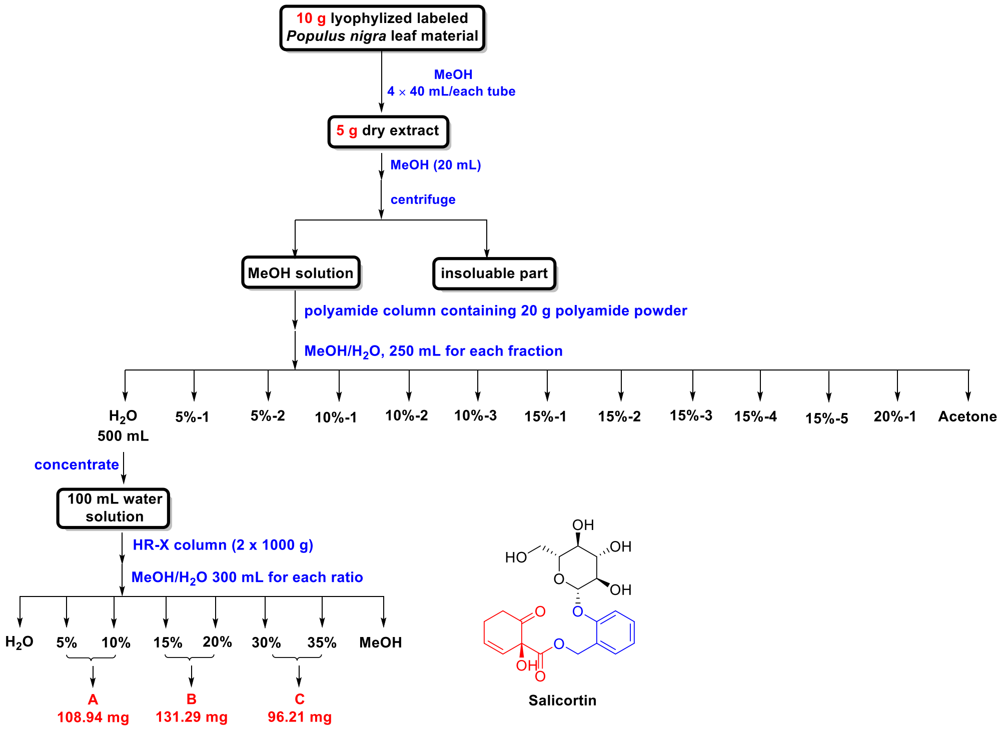

Figure S1.2 Isolation procedure of ^{13}C Salicortin from labeled *Populus nigra* leaves

7. Leaf coating experiment with ^{13}C-salicortin

Freshly cut leaves of *P. nigra* were used for feeding experiments. The leaf petioles were inserted into 2 mL Eppendorf micro reaction vessels. Lids of the vessels were removed for convenient handling prior to being filled with tap water. The opening of the vessels was sealed with Parafilm® fastening the leaf petioles to prevent the water in the vessel from spilling. An aqueous solution of [U-^{13}C] salicortin (25 mg/mL) was spotted evenly on the

surface of three poplar leaves (10 × 20 μL droplets per leaf). Similarly, unlabeled salicortin aqueous solution (25 mg/mL) was coated on the surface of three poplar leaves. Control leaves were spotted with water in an analogous fashion. Leaves were left under the fume hood for 3 h, allowing the droplets to dry completely. Subsequently, the [U-^{13}C] salicortin-, unlabeled salicortin-coated leaves and the control leaves were transferred into Petri dishes (diameter) for feeding experiments. Each dish contained one leaf and six *C. tremulae* adults, which were placed onto the surface of each leaf. The insects were kept in the arena until the leaves had been completely consumed. Feces collected from the control experiments, unlabeled salicortin and the stable isotope feeding experiments, respectively, were pooled in three batches (**Figures S1.1**). Feces were collected and analyzed using above method.

Supplementary figures

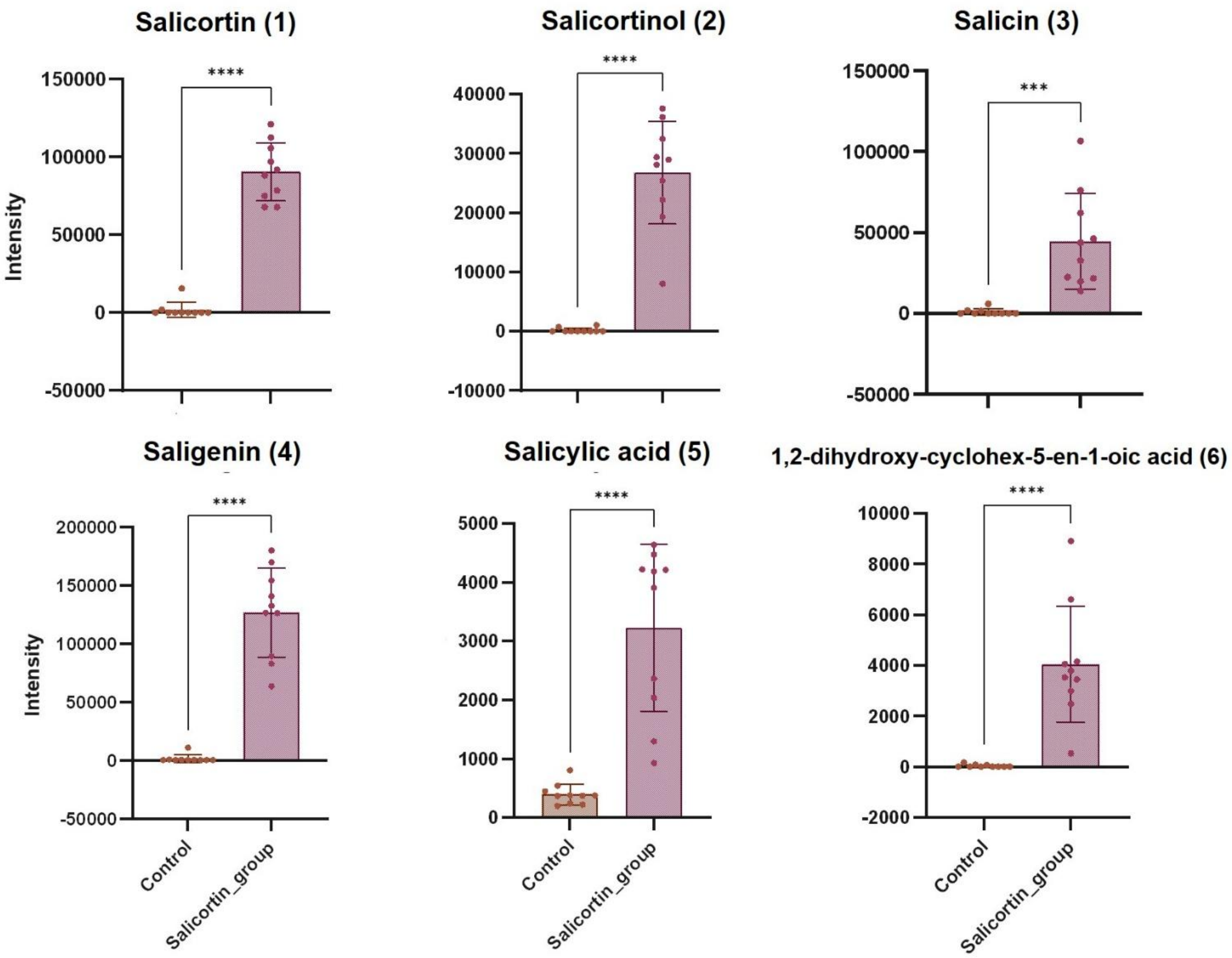

Figure S1.3 The relative content difference of salicortin (**1**) and its related metabolites (**2**–**6**) in the fecal extract of control and salicortin coating group with *Salix viminalis*, measured by LC-Q-TOF-MS. The student's t test with ****P* < 0.005, *****P* < 0.001 vs Control. The data are presented as the mean ± SD (n = 10).

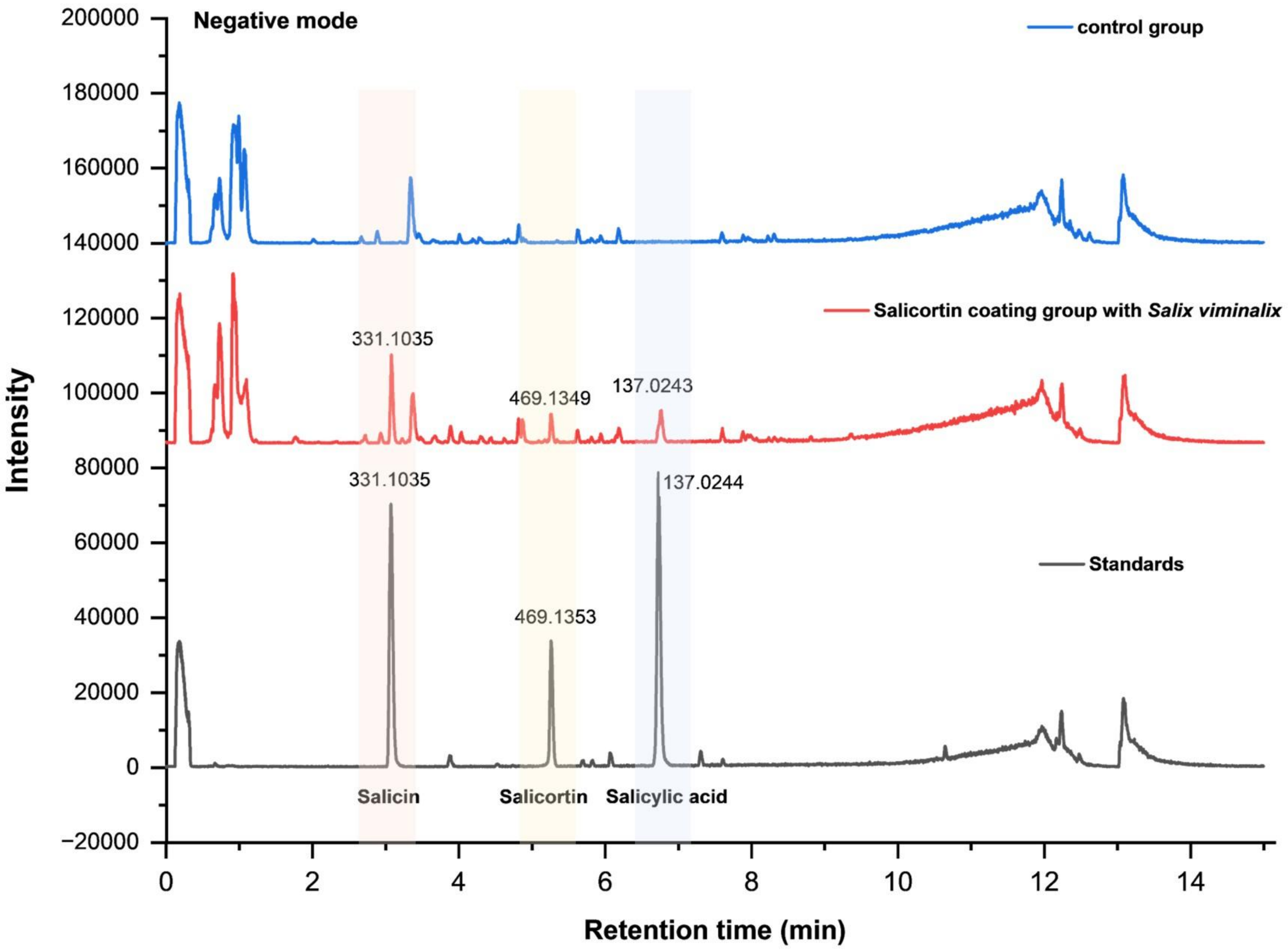

Figure S1.4 Identification of salicortin and its metabolites (salicin and salicylic acid) in the fecal extract of the control and salicortin coating group with *Salix viminalis* were accomplished by comparison with authentic reference standards utilizing LC-HRESI-MS (negative mode).

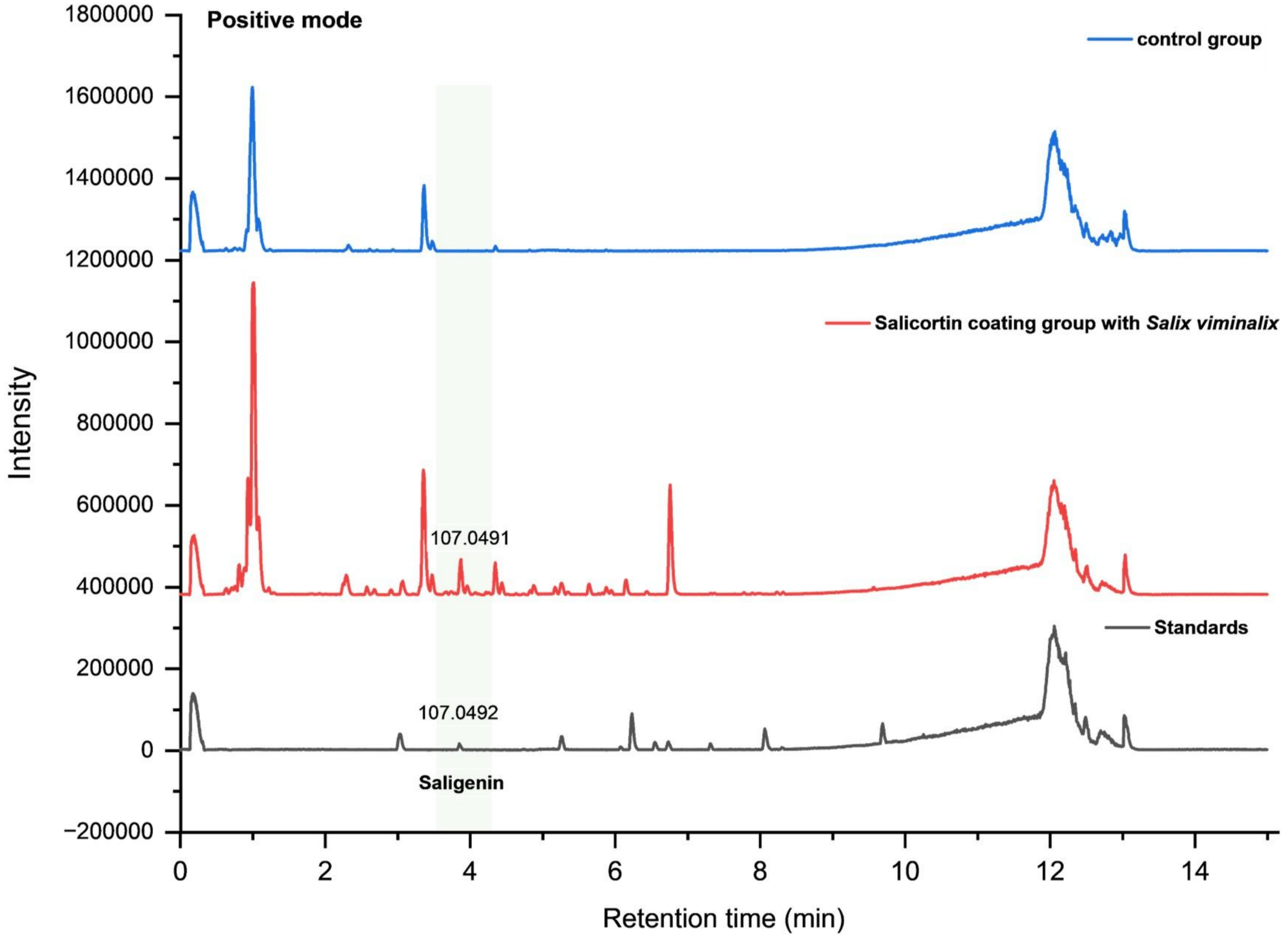

Figure S1.5 Identification of salicortin and its metabolites (saligenin) in the fecal extract of the control and salicortin coating group with *Salix viminalis* were accomplished by comparison with authentic reference standards utilizing LC-HRESI-MS (positive mode).

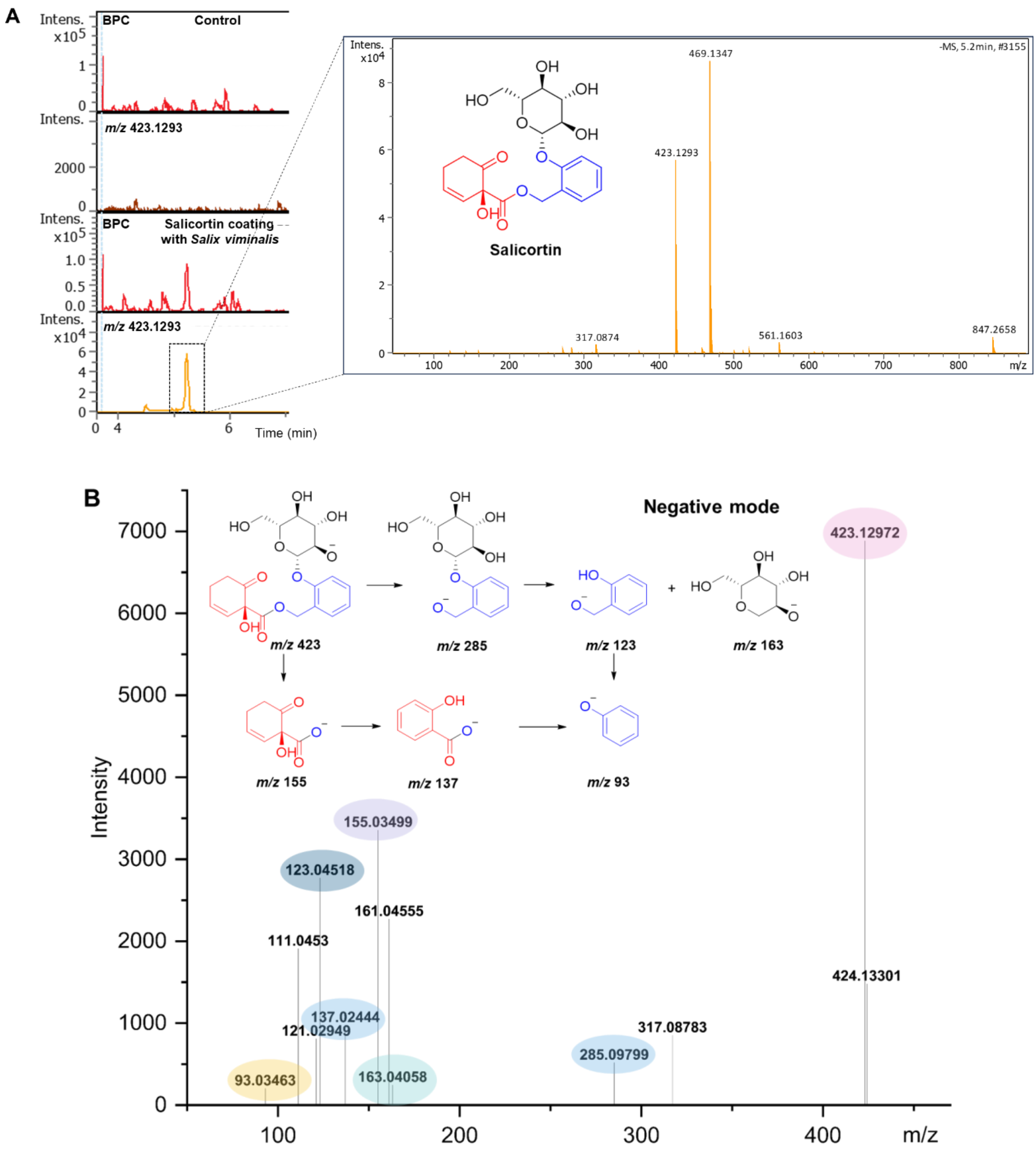

Figure S1.6 (A) Full scan HRMS spectrum (negative mode) of salicortin (**1**) in the feces extract of the salicortin coating group with *Salix viminalis*, observed peaks at *m/z* 423.1293 [M – H]⁻ and 469.1347 [M + HCOOH – H]⁻, respectively. The left panel shows base peak chromatograms (BPC) and extracted ion chromatograms at *m/z* 423.1293 for the feces extracts of the control group and for the salicortin coating group. (B) The measured MS2 spectrum and the proposed MS2 fragmentation pattern of salicrotin (**1**) in the negative mode.

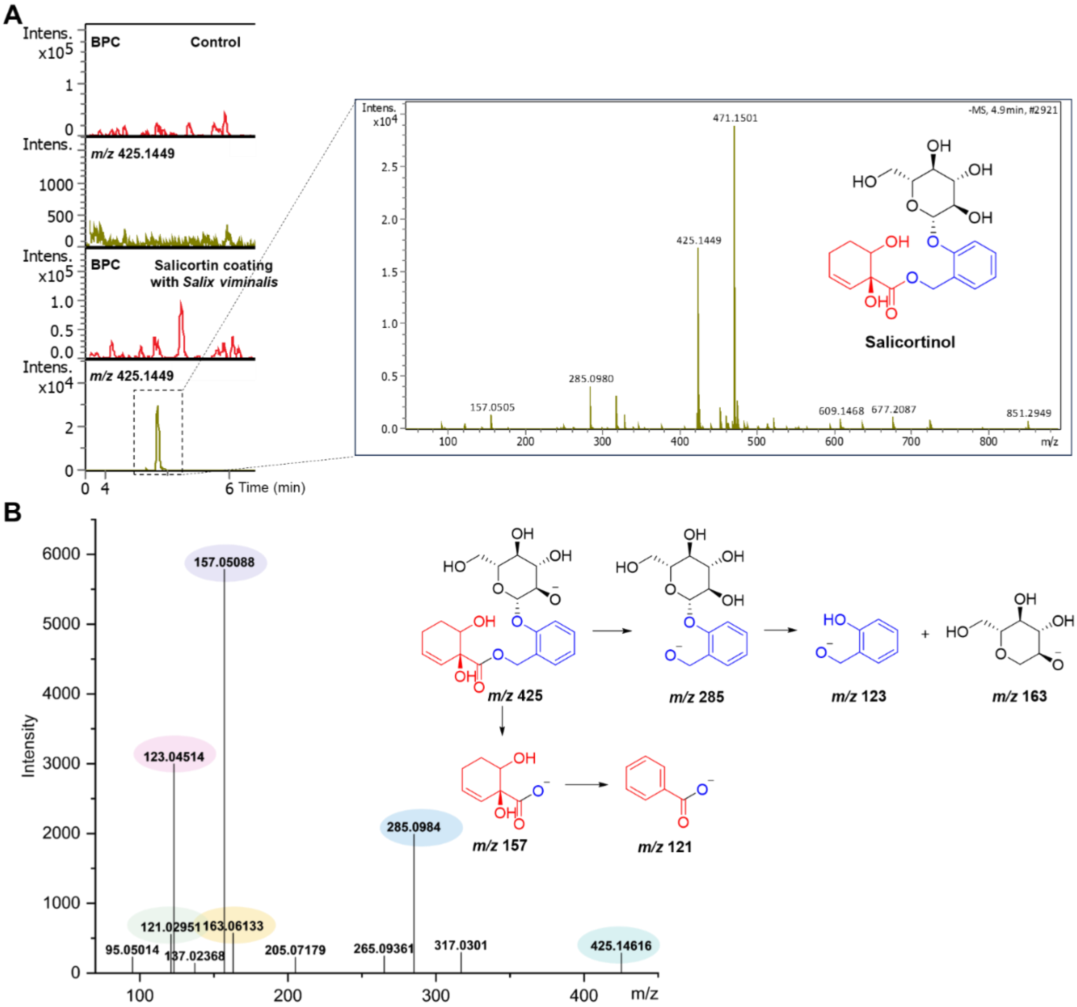

Figure S1.7 (A) Full scan HRMS spectrum (negative mode) of salicortinol (**2**) in the feces extract of the salicortin coating group with *Salix viminalis*, observed peaks at *m/z* 425.1449 [M – H]⁻ and 471.1501 [M + HCOOH – H]⁻, respectively. The left panel shows base peak chromatograms (BPC) and extracted ion chromatograms at *m/z* 425.1449 for the feces extracts of the control group and for the salicortin coating group. (B) The measured MS2 spectrum and the proposed MS2 fragmentation pattern of salicrotinol (**2**) in the negative mode.

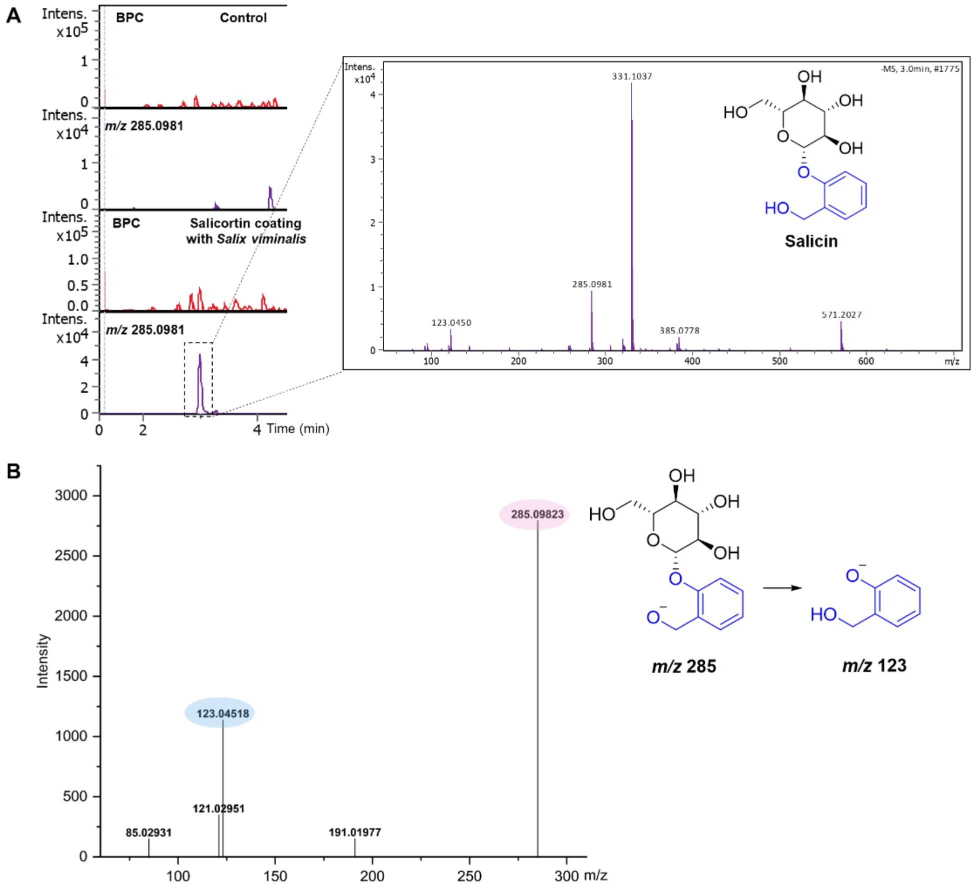

Figure S1.8 (A) Full scan HRMS spectrum (negative mode) of salicin (**3**) in the feces extract of the salicortin coating group with *Salix viminalis*, observed peaks at *m/z* 285.0981 $[M - H]^-$ and 331.1037 $[M + HCOOH - H]^-$, respectively. The left panel shows base peak chromatograms (BPC) and extracted ion chromatograms at *m/z* 285.0981 for the feces extracts of the control group and for the salicortin coating group. (B) The measured MS^2 spectrum and the proposed MS^2 fragmentation pattern of salicin (**3**) in the negative mode.

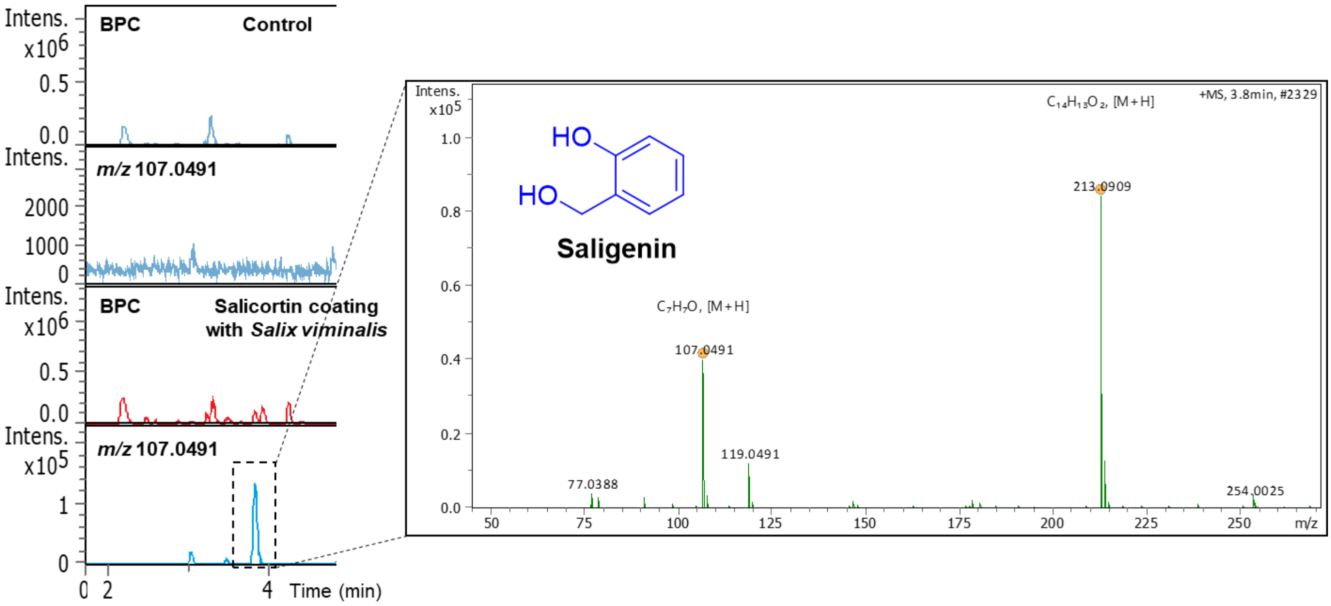

Figure S1.9 Full scan HRMS spectrum (positive mode) of saligenin (**4**) in the feces extract of the salicortin coating group with *Salix viminalis*, observed peaks at *m/z* 107.0491 $[M + H]^+$ and at *m/z* 213.0909 $[2M + H]^+$. The left panel shows base peak chromatograms (BPC) and extracted ion chromatograms at *m/z* 107.0491 for the feces extracts of the control group and for the salicortin coating group.

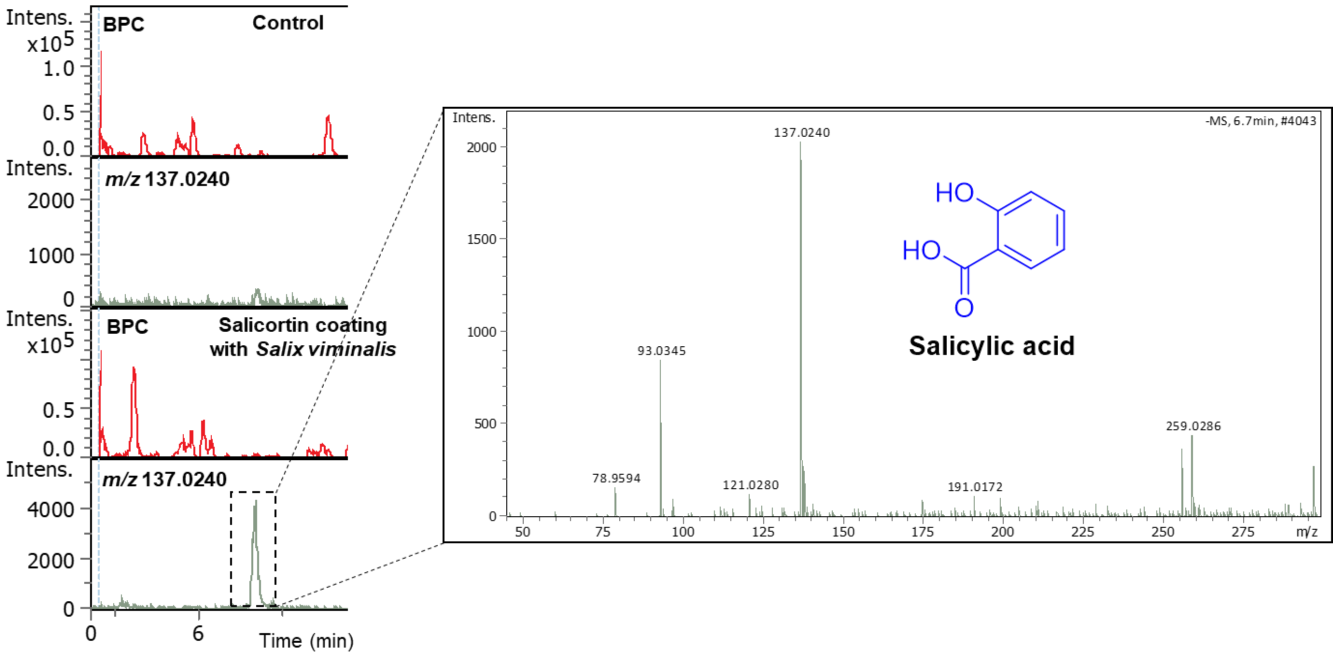

Figure S1.10 Full scan HRMS spectrum (negative mode) of salicylic acid (**5**) in the feces extract of the salicortin coating group with *Salix viminalis*, observed peak at *m/z* 137.0204 [M – H]⁻. The left panel shows base peak chromatograms (BPC) and extracted ion chromatograms at *m/z* 137.0240 for the feces extracts of the control group and for the salicortin coating group.

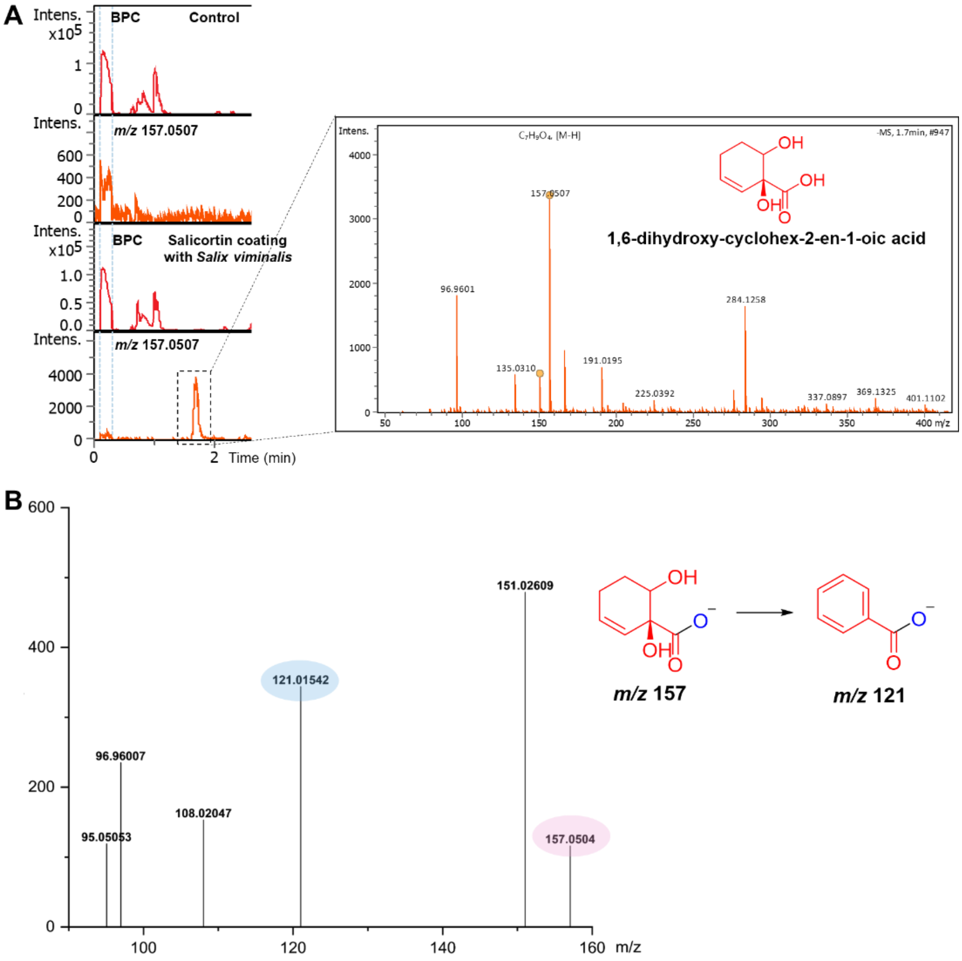

Figure S1.11 (A) Full scan HRMS spectrum (negative mode) of 1,2-dihydroxy-cyclohex-5-en-1-oic acid (**6**) in the feces extract of the salicortin coating group with *Salix viminalis*, observed peak at *m/z* 157.0507 [M – H]⁻. The left panel shows base peak chromatograms (BPC) and extracted ion chromatograms at *m/z* 157.0507 for the feces extracts of the control group and for the salicortin coating group. (B) The measured MS^2 spectrum and the proposed MS^2 fragmentation pattern of 1,2-dihydroxy-cyclohex-5-en-1-oic acid (**6**) in the negative mode.

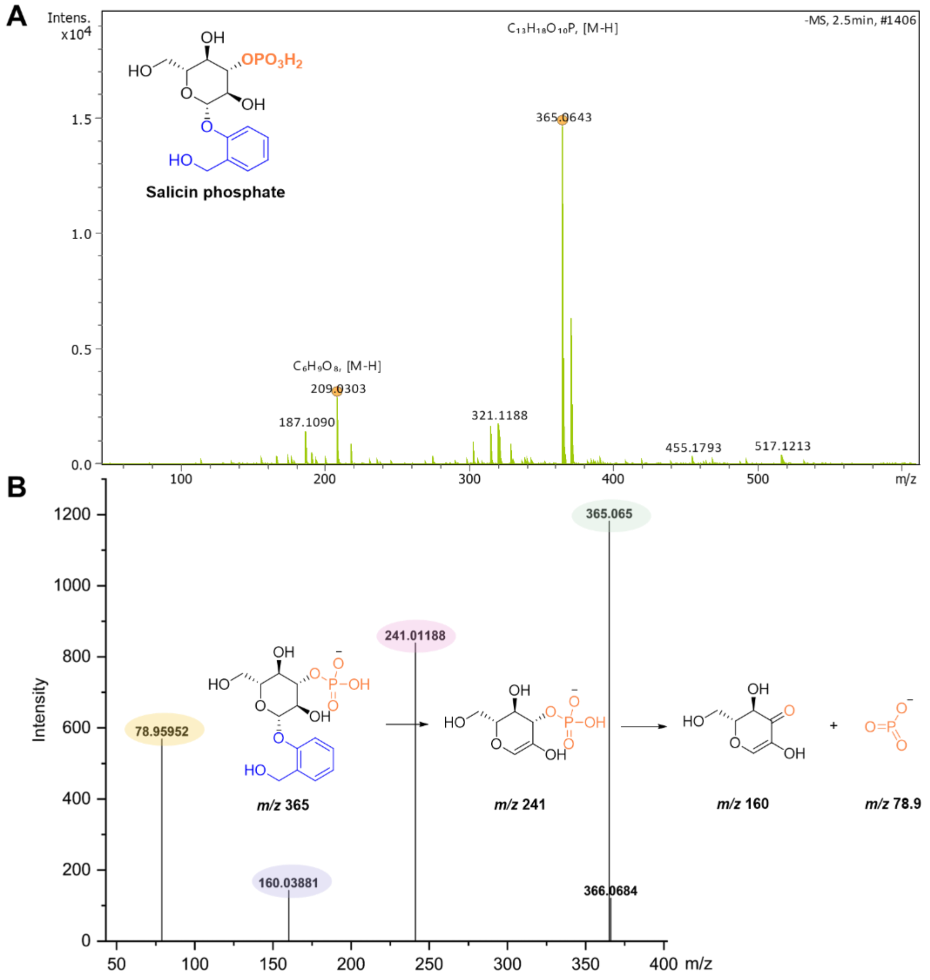

Figure S1.12 (A) Full scan HRMS spectrum (negative mode) of salicin phosphate (**8**) in the feces extract of the salicortin coating group with *Salix viminalis*, observed peak at *m/z* 365.0643 $[M-H]^-$ (Retention time: 2.5 min). (B) The measured MS^2 spectrum and the proposed MS^2 fragmentation pattern of salicin phosphate (**8**) in the negative mode.

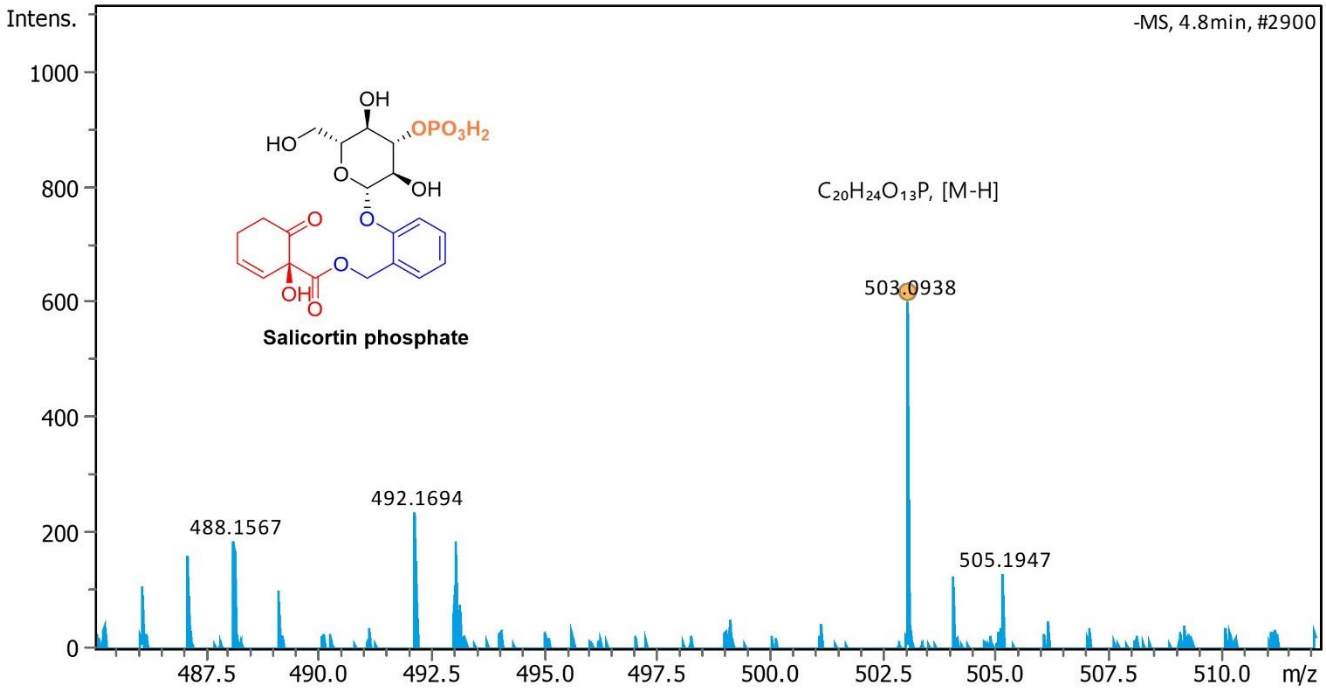

Figure S1.13 Full scan HRMS spectrum (negative mode) of salicortin phosphate (**7**) in the feces extract of the salicortin coating group with *Salix viminalis*, observed peak at *m/z* 503.0934 $[M – H]^{–}$.

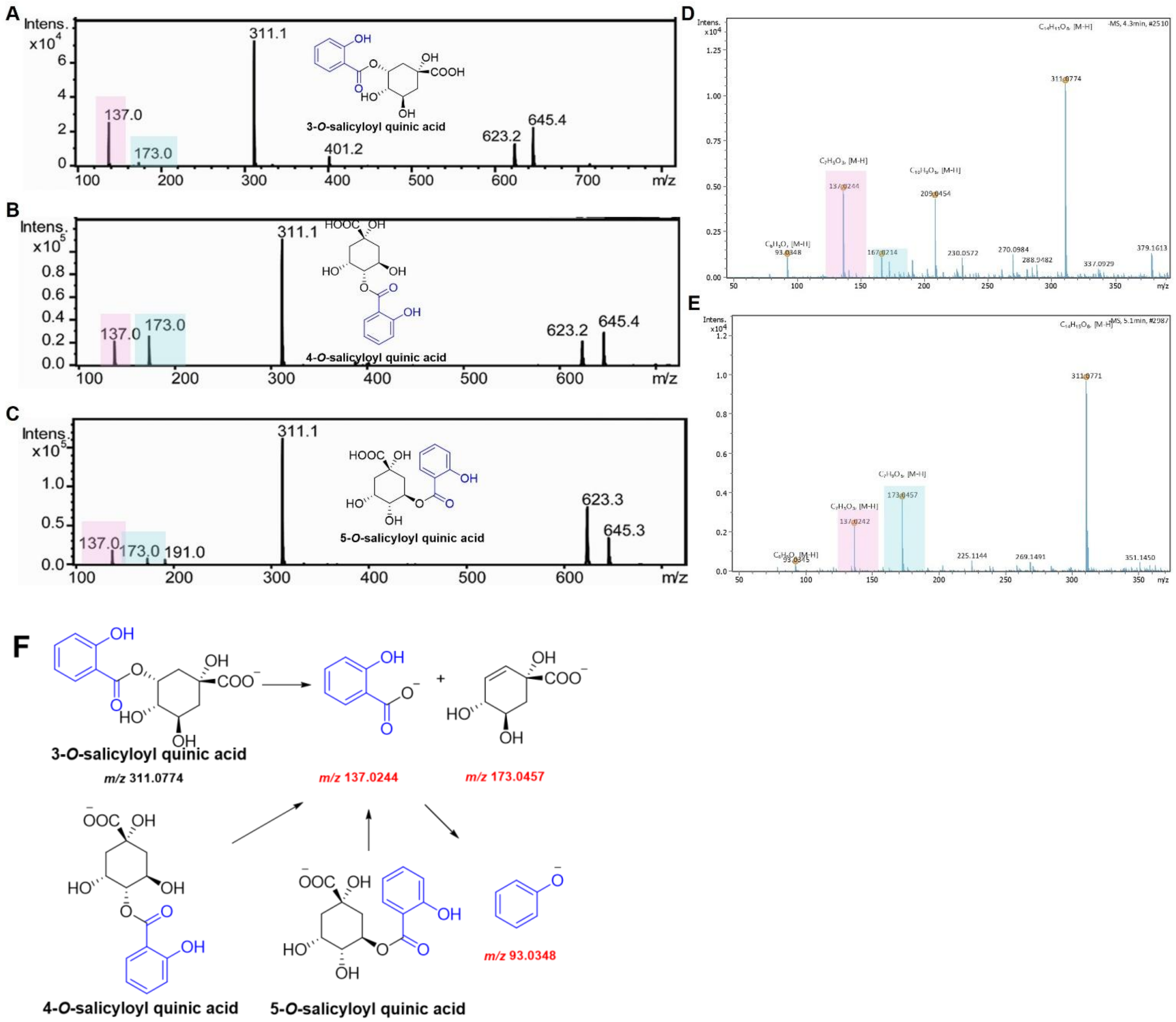

Figure S1.14 (A-C) Full scan MS spectra of 3-*O*-salicyloyl quinic acid, 4-*O*-salicyloyl quinic acid, and 5-*O*-salicyloyl quinic acid reported by Felix et al. (2). (D, E) Full scan HRMS spectra (negative mode) of two salicyloyl quinic acids (**9** and **10**) in the feces extract of the salicortin coating group with *Salix viminalis*, observed peaks at *m/z* 311.0774 [M – H]⁻ (Retention time: 4.3 min) and 311.0771 [M – H]⁻ (Retention time: 5.1 min). (F) The plausible fragmentation pattern for salicyloyl qunic acids. (The structures of the two salicyloyl quinic acids (**9** and **10**) were assigned as 3-*O*-salicyloyl quinic acid (**9**) and 4-*O*-salicyloyl quinic acid (**10**). This deduction was based on the characteristic relative abundance of the fragment ions at *m/z* 137.0244 and *m/z* 173.0457.)

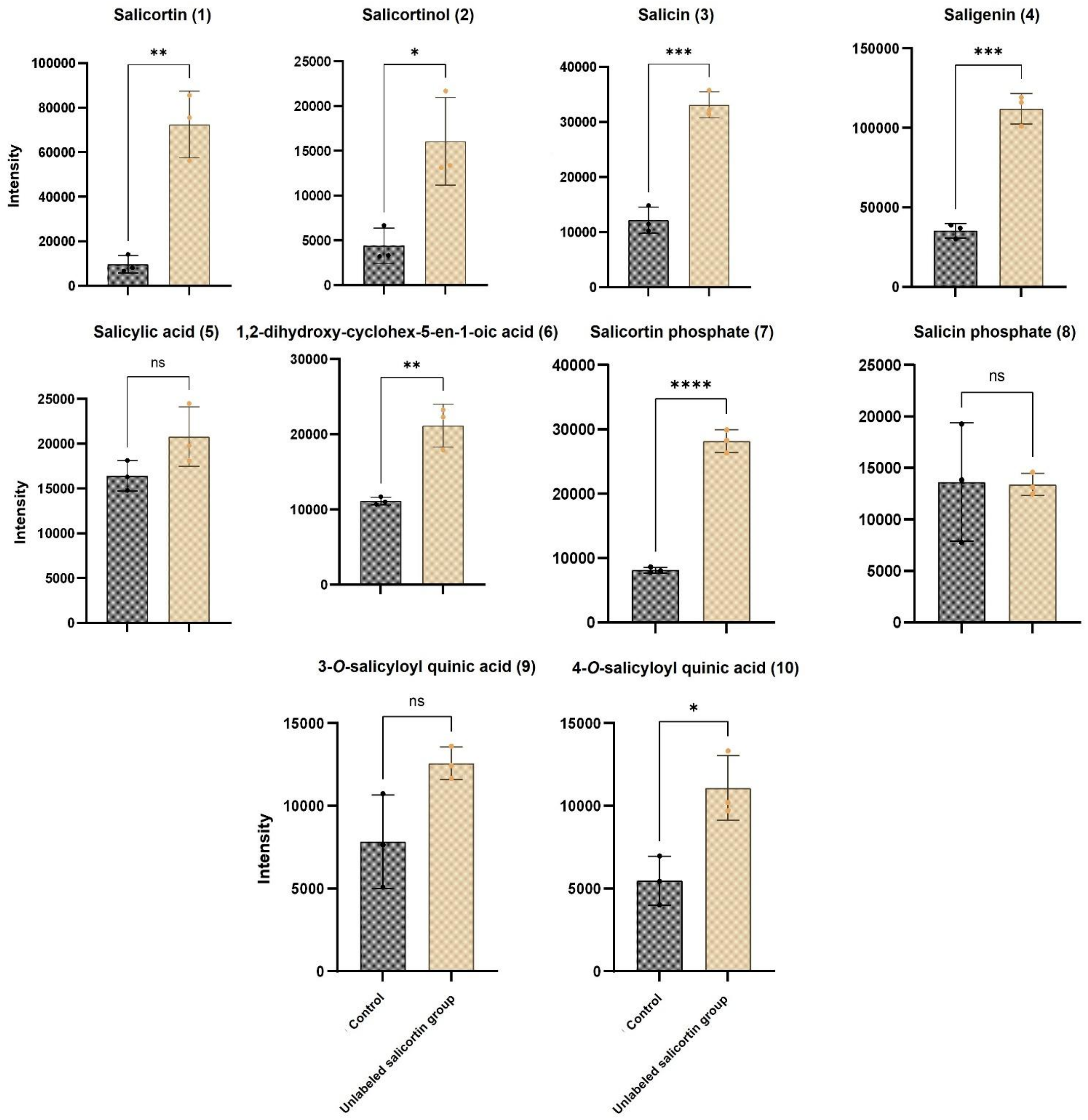

Figure S1.15 The relative contents of salicortin (**1**) and its metabolites (**2**–**10**) in the feces extract between unlabeled salicortin coating group and control group with *Populus nigra*. The student's t test with **P* < 0.05, ** *P* < 0.01, *** *P* < 0.005, *****P* < 0.001 vs Control. The data are presented as the mean ± SD (n = 3).

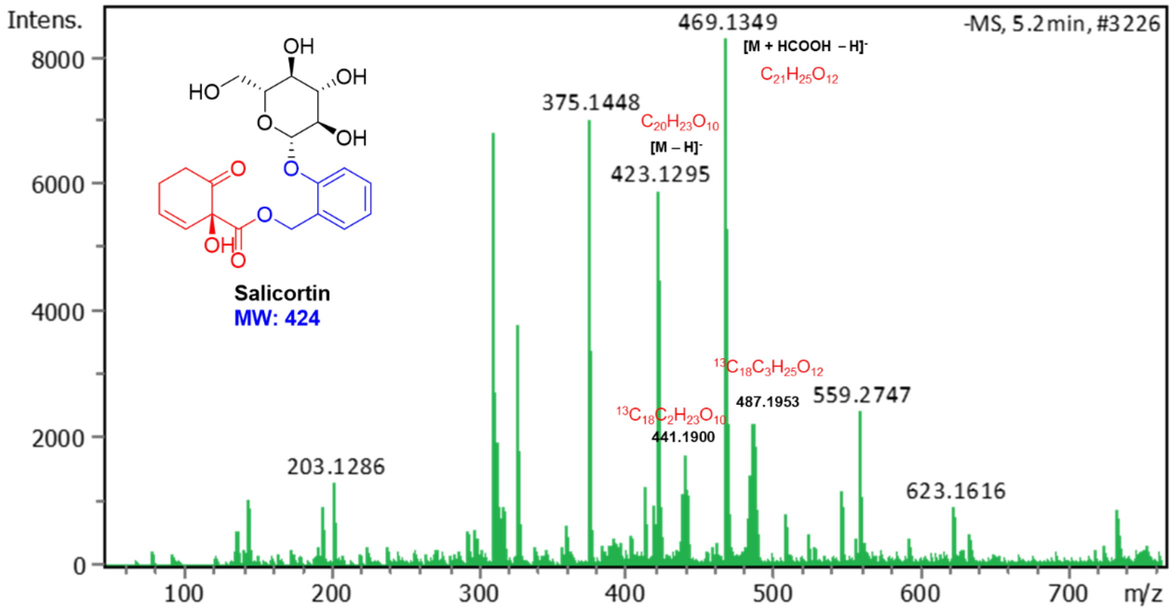

Figure S1.16 Full scan HRMS spectrum (negative mode) of salicortin (**1**) in the feces extracts of the ^{13}C salicortin coating group with *Populus nigra*. Observed the peaks of unlabeled salicortin at *m/z* 423.1295 [M – H]⁻ and 469.1349 [M + HCOOH – H]⁻, and peaks of ^{13}C salicortin at *m/z* 441.1900 [M + 18 – H]⁻ and 487.1953 [M + 18 + HCOOH – H]⁻.

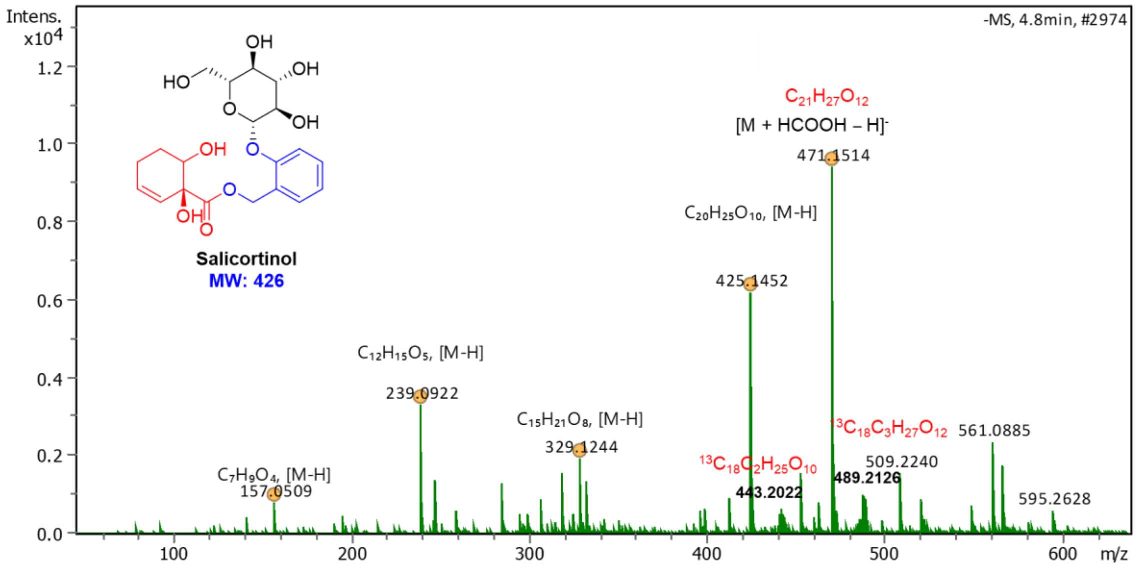

Figure S1.17 Full scan MS spectrum (negative mode) of salicortinol (**2**) in the feces extracts of the ^{13}C salicortin coating group with *Populus nigra*. Observed the peaks of unlabeled salicortinol at *m/z* 425.1452 [M – H]⁻ and 471.1514 [M + HCOOH – H]⁻, and peaks of ^{13}C salicortinol at *m/z* 443.2022 [M + 18 – H]⁻ and 489.2128 [M + 18 + HCOOH – H]⁻.

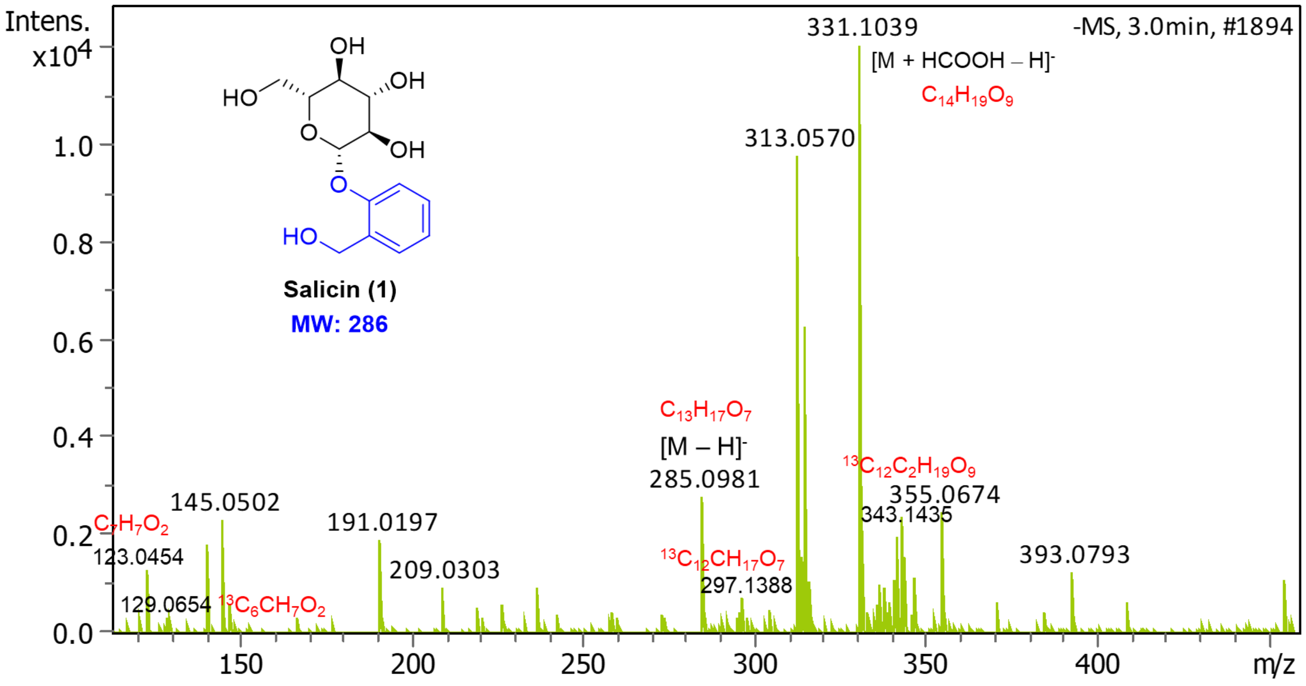

Figure S1.18 Full scan MS spectrum (negative mode) of salicin (**3**) in the feces extracts of the ^{13}C salicortin coating group with *Populus nigra*. Observed the peaks of unlabeled salicin at *m/z* 285.0981 [M – H]$^{–}$ and 331.1039 [M + HCOOH – H]$^{–}$, and peaks of ^{13}C salicin at *m/z* 297.1388 [M + 12 – H]$^{–}$ and 343.1435 [M + 12 + HCOOH – H]$^{–}$.

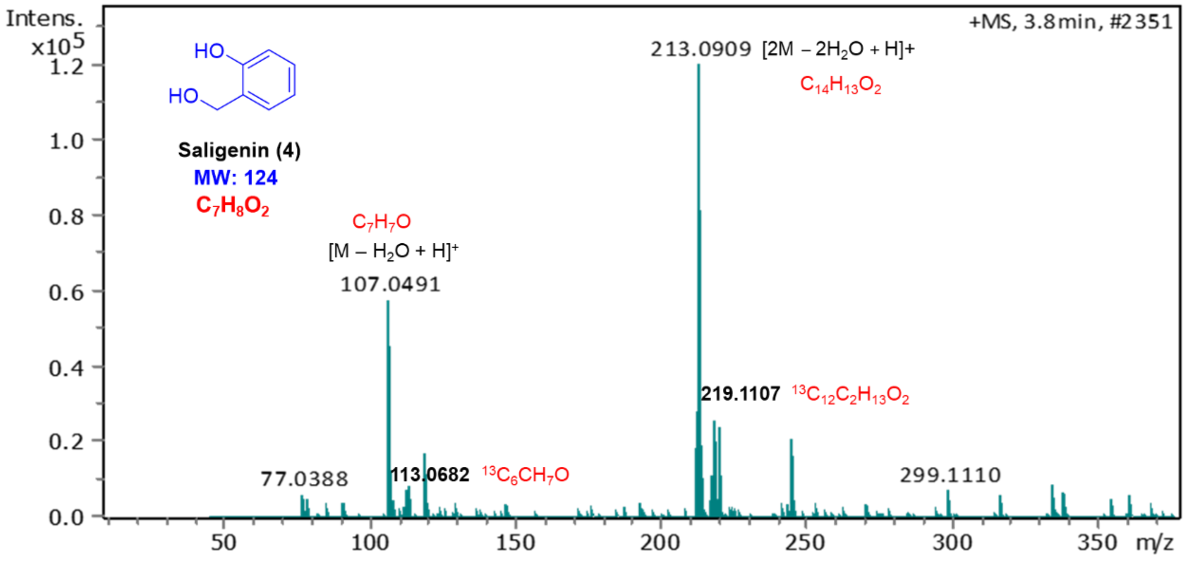

Figure S1.19 Full scan MS spectrum (positive mode) of saligenin (**4**) in the feces extracts of the ^{13}C salicortin coating group with *Populus nigra*. Observed the peaks of unlabeled saligenin at *m/z* 107.0491 [M – H_2O + H]$^{+}$ and 213.0909 [2M – 2H_2O + H]$^{+}$, and peaks of ^{13}C saligenin at *m/z* 113.0682 [M – H_2O + 6 + H]$^{+}$ and 219.1107 [2M – 2H_2O + H +12]$^{+}$.

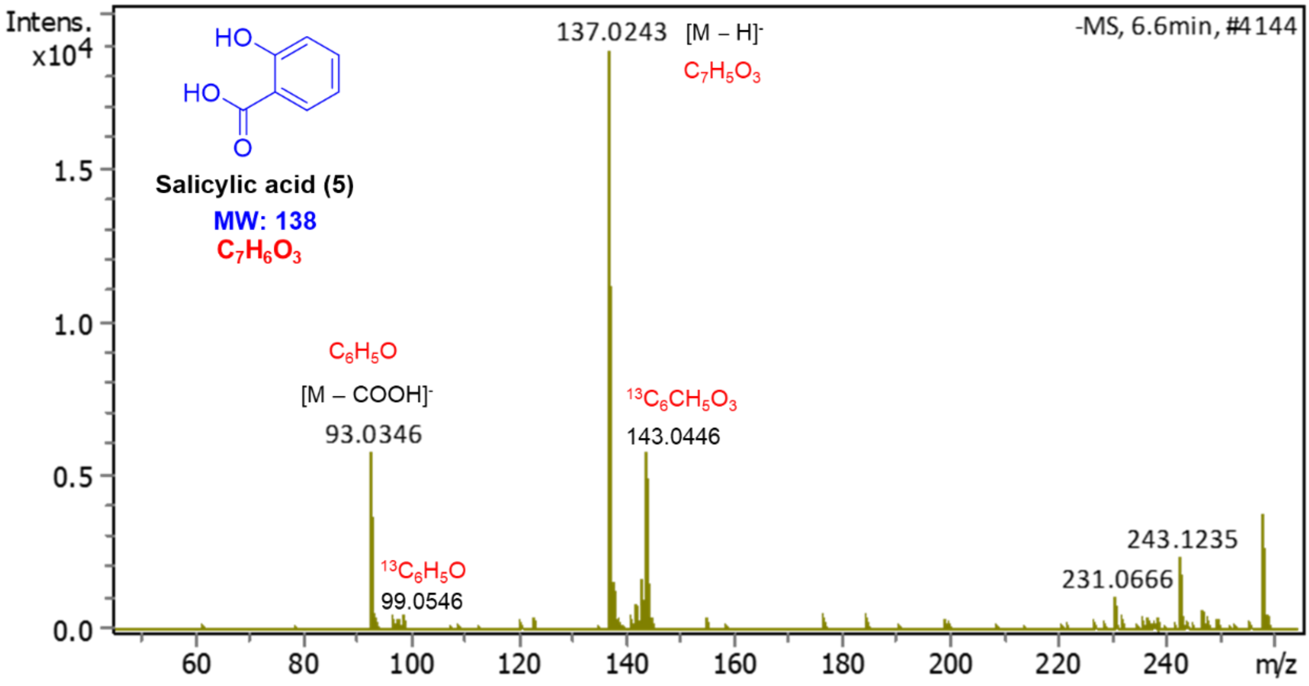

Figure S1.20 Full scan MS spectrum (negative mode) of salicylic acid (**5**) in the feces extracts of the ^{13}C salicortin coating group with *Populus nigra*. Observed the peaks of unlabeled saligenin at *m/z* 137.0243 [M – H]⁻ and 93.0346 [M – COOH]⁻, and peaks of ^{13}C salicylic acid at *m/z* 143.0446 [M + 6 – H]⁻ and 99.0546 [M + 6 – COOH]⁻.

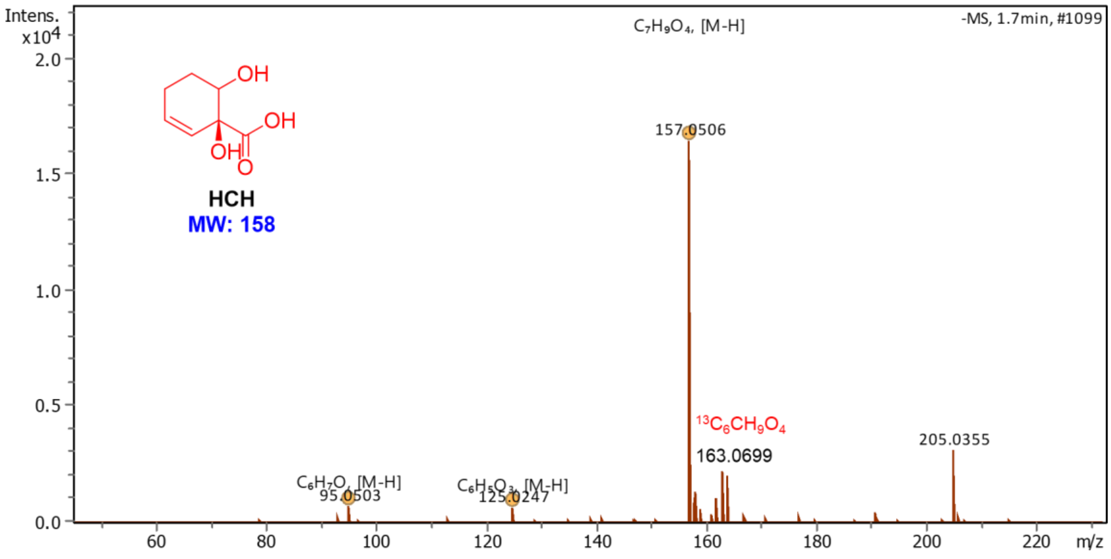

Figure S1.21 Full scan MS spectrum (negative mode) of 1,2-dihydroxy-cyclohex-5-en-1-oic acid (**6**) in the feces extracts of the ^{13}C salicortin coating group with *Populus nigra*. Observed the peak of unlabeled 1,2-dihydroxy-cyclohex-5-en-1-oic acid at *m/z* 157.0506 [M – H]⁻, and peak of ^{13}C 1,2-dihydroxy-cyclohex-5-en-1-oic acid at *m/z* 163.0699 [M + 6 – H]⁻.

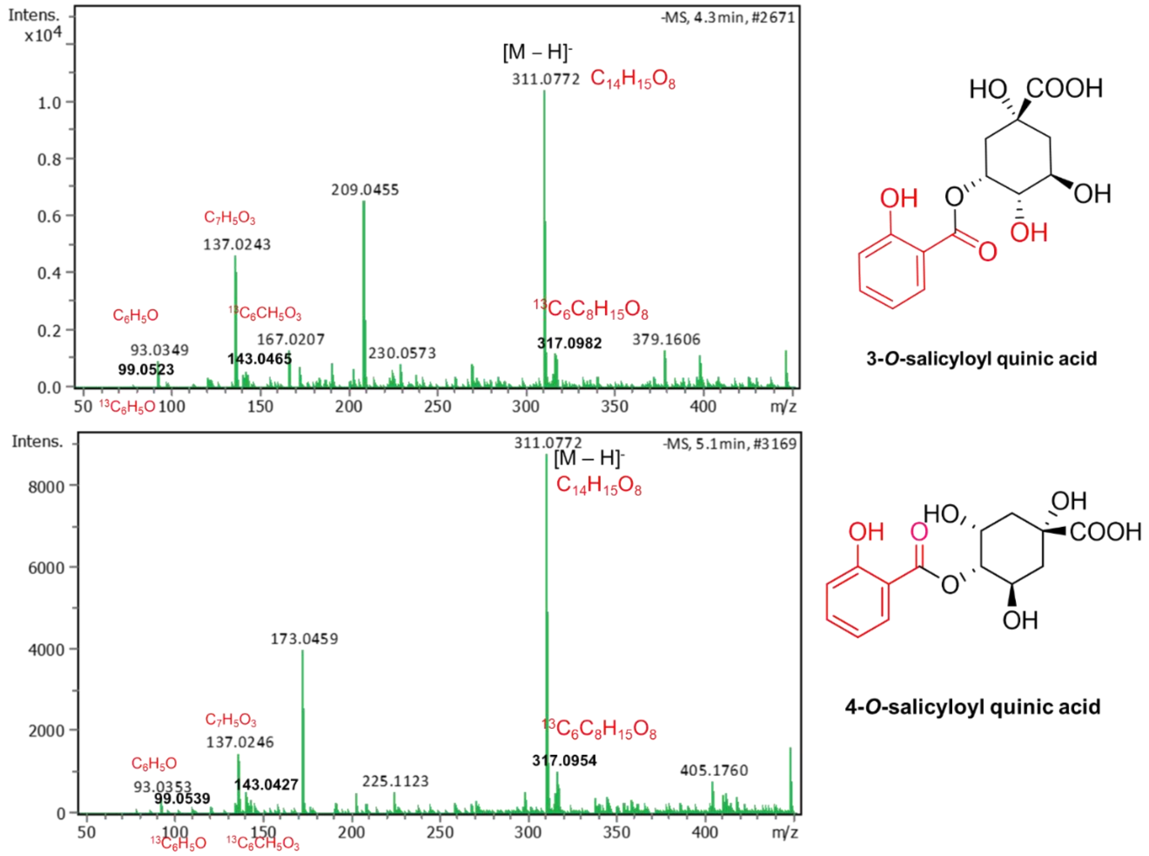

Figure S1.22 Full scan MS spectra (negative mode) of salicylolyl quinic acids in the feces extracts of the ^{13}C salicortin coating group with *Populus nigra*. Observed peaks of unlabeled salicylolyl quinic acids at *m/z* 311.0772 [M – H]⁻ (retention time: 4.3 min) and 311.0772 [M – H]⁻ (Retention time: 5.1 min), and peaks of ^{13}C salicylolyl quinic acids at *m/z* 317.0982 [M + 6 – H]⁻ (retention time: 4.3 min) and 317.0954 [M + 6 – H]⁻ (Retention time: 5.1 min).

Table S1.1 Molecular weight and molecular formula of labeled compounds.

Compounds	Retention time (min)	Unlabeled peak (*m/z*)	Molecular formula	Labeled peak (*m/z*)	Labeled molecular formula
Salicortin (**1**)	5.2	423.1293 [M – H]⁻<br>469.1347 [M + COOH – H]⁻	$C_{20}H_{23}O_{10}$<br>$C_{21}H_{23}O_{12}$	441.1900 [M – H + 18]⁻	$^{13}C_{18}C_2H_{24}O_{10}$
Salicortinol (**2**)	4.9	425.1449 [M – H]⁻<br>471.1501 [M + COOH – H]⁻	$C_{20}H_{25}O_{10}$<br>$C_{21}H_{25}O_{12}$	443.2022 [M – H + 18]⁻	$^{13}C_{18}C_2H_{25}O_{10}$
Salicin (**3**)	3.0	285.0981 [M – H]⁻<br>331.1037 [M + COOH – H]⁻	$C_{13}H_{17}O_7$<br>$C_{14}H_{17}O_9$	297.1388 [M – H + 12]⁻	$^{13}C_{12}CH_{17}O_7$
Saligenin (**4**)	3.8	107.0491 [M – H_2O + H]⁺<br>213.0909 [2M – $2H_2O$ + H]⁺	C_7H_7O<br>$C_{14}H_{13}O_2$	113.0682 [M – H_2O + H + 6]⁺	$^{13}C_6CH_7O$
salicylic acid (**5**)	6.7	137.0240 [M – H]⁻	$C_7H_5O_3$	143.0446 [M – H + 6]⁺	$^{13}C_6CH_5O_3$
1,2-dihydroxy-cyclohex-5-en-1-oic acid (**6**)	1.7	157.0507 [M – H]⁻	$C_7H_9O_4$	163 [M – H + 6]⁺	$^{13}C_6CH_9O_4$
Salicortin phosphate (**7**)	5.6	503.0938 [M – H]⁻	$C_{20}H_{25}O_{13}P$	521.2374 [M – H + 18]⁻	$^{13}C_{18}C_2H_{25}O_{13}P$
Salicin phosphate (**8**)	2.5	365.0643 [M – H]⁻	$C_{13}H_{17}O_{10}P$	377.1073 [M – H + 12]⁻	$^{13}C_{12}CH_{17}O_{10}P$
3-*O*-salicyloyl quinic acid (**9**)	4.3	311.0774 [M – H]⁻	$C_{14}H_{15}O_8$	317.0982 [M – H + 6]⁺	$^{13}C_6C_8H_{15}O_8$
4-*O*-salicyloyl quinic acid (**10**)	5.1	311.0771 [M – H]⁻	$C_{14}H_{15}O_8$	317.0954 [M – H + 6]⁺	$^{13}C_6C_8H_{15}O_8$
Chrysotremulin A (**11**)	6.0	315.1344 [M + H]⁺	$C_{17}H_{19}N_2O_4$	321.1494 [M + H + 6]⁺	$^{13}C_6{}^{12}C_{11}H_{18}N_2O_4$
Chrysotremulin B (**12**)	5.1	296.0922 [M + H]⁺	$C_{17}H_{14}NO_4$	302.0748 [M + H + 6]⁺	$^{13}C_6{}^{12}C_{11}H_{13}NO_4$
Chrysotremulin C (**13**)	6.6	252.1021 [M + H]⁺	$C_{16}H_{14}NO_2$	258.1202 [M + H + 6]⁺	$^{13}C_6{}^{12}C_{10}H_{13}NO_2$

SI-2: The discovery and structural elucidation of chrysotremulins A-C

1. The extraction and isolation of chrysotremulins A–C (11–13) from *C. tremulae* feces

Chrysomela tremulae larvae were hatched from eggs and reared on *Populus nigra* leaves in the laboratory. A total of 43.6 g air-dried feces of adult *C. tremulea* beetles was split into six 50 mL tubes and extracted by adding MeOH (45 mL for each tube) and 2 mm stainless steel balls (6 balls for each tube). All tubes were agitated using a shaker (Novotron AI82 K incubation shaker, Bottmingen, Suisse) and the extracts were then filtered (paper filters, MN 615 ¼, 125 mm, Macherey-Nagel). Extraction was repeated three times. All extracts were combined and evaporated under reduced pressure using a rotary evaporator R-114 (Buchi Labortechnik, Flawil, Switzerland) and a Genevac HT-4X vacuum centrifuge (Genevac, Ipswich, UK), resulting in 20 g dried crude extract. This dry matter was then dissolved in MeOH (20 mL) and filtered again by using filter paper (MN 615 ¼, 125 mm, Macherey-Nagel). The filtered solution was mixed with 30 g reverse phase C18 material (40-63 µm for liquid chromatography, Sigma Aldrich, Merck, Germany). After the evaporation of MeOH, the dry mixture was subjected to a glass column with 60 g reverse phase C18 material (40-63 µm for liquid chromatography, Sigma Aldrich, Merck, Germany). The column was eluted with different ratios of MeOH/H_2O, and 12 fractions (F-1→F-12) were obtained [F-1: 5% MeOH (v:v) (500 mL), F-2: 10% MeOH (500 mL), F-3: 15% MeOH (500 mL), F-4: 20% MeOH-1 (400 mL), F-5: 20% MeOH-2 (200 mL), F-6: 25% MeOH (1000 mL), F-7: 30% MeOH (1000 mL), F-8: 35% MeOH-1 (400 mL), F-9: 35% MeOH-2 (400 mL), F-10: 35% MeOH-3 (600 mL), F-11: 35% MeOH-4 (400 mL), and F-12: 100% MeOH (500 mL)].

The twelve fractions were analyzed by HPLC (series 1100, Agilent, USA) coupled to an Esquire 6000 ESI-ion trap mass spectrometer (Bruker Daltonics, Bremen, Germany) operated in alternating ionization mode and scanning a mass range from *m/z* = 60–1200. Nitrogen served as nebulizer (35 psi) and drying gas (11 1/min, 330 ºC). A reverse phase column (EC 250/4.6 Nucleodur Sphinx, RP 5 µm, Macherey-Nagel, Düren, Germany) was used for HPLC and gradient elution with 0.2 % formic acid (Fisher Scientific, Waltham, Massachusetts, USA) in MilliQ® water (Synthesis A10 Millipore Water, Merck, Darmstadt, Germany) (A) and acetonitrile (HPLC LC-MS grade, VWR International, Darmstadt, Germany) (B) was applied using the following parameters: 14% B (0 min), 14–58 % B (0–22 min), 100 % B (22.1–25 min), 14 % B (25.1–30 min). Column temperature was set to 25 °C and the solvent flow rate was 1 mL min^{-1}. Analysis of LC-MS showed that F-4 contained chrysotremulin B (**12**, *m/z*: 296 $[M + H]^+$, 190 $[M - C_7H_7O + H]^+$) and F-10 contained chrysotremulins A (**11**, *m/z*: 315 $[M + H]^+$, 209 $[M - C_7H_7O + H]^+$, 192 $[M - C_7H_7O - NH_3 + H]^+$) and C (**13**, *m/z*:

252 [M + H]$^+$, 146 [M – C_7H_7O + H]$^+$).

Then, F-4 (20 mg) was dissolved in 1.5 mL MeOH and fractionated on a reversed phase column (Supelcosil LC-18-DB semi-prep, 250 × 10 mm × 5 μm) isocratically eluted with 20% acetonitrile (CH_3CN) /H_2O containing 0.2% formic acid (total time: 11 min, flow rate: 3 mL/min, injection volume: 20 μL). This resulted in three collected sub-fractions (F-4-1→F-4-3). F-4-2 (12 mg) was further purified by a reverse phase column (EC 250/4.6 Nucleodur π^2, RP 5 μm, Macherey-Nagel, Düren, Germany) using an isocratic solvent of 27% CH_3CN (in water with 0.2% formic acid) at 1 ml/min (injection volume: 10 μL) to yield chrysotremulin B (**12**, t_R = 18.7 min, 0.51 mg).

In addition, F-10 (231 mg) was dissolved in 30 mL MeOH and 15 mL was taken out for the further isolation using the reversed phase column (Supelcosil LC-18-DB semi-prep, 250 × 10 mm × 5 μm). The elution was 22% CH_3CN (in water with 0.2% formic acid) (total time: 19 min, flow rate: 3 mL/min, injection volume: 20 μL). Fr. 10-1→Fr. 10-3 were obtained and Fr. 10-2 was further purified using a reverse phase column (EC 250/4.6 Nucleodur π^2, RP 5 μm, Macherey-Nagel, Düren, Germany) using an isocratic elution of 35% CH_3CN (in water with 0.2% formic acid) at 1 ml/min (injection volume: 50 μL) to yield chrysotremulin C (**13**, t_R = 10.47 min, 0.62 mg). Similarly, chrysotremulin A (**11**, t_R = 13.4 min, 9.2 mg) was obtained by Nucleodur π^2 column (EC 250/4.6 Nucleodur π^2, RP 5 μm, Macherey-Nagel, Düren, Germany) with the elution of 22% CH_3CN (in water with 0.2% formic acid) (flow rate: 1 mL/min, injection volume: 30 μL).

2. Identification of chrysotremulins A–C.

NMR spectra for the structure elucidation of chrysotremulins A–C (**11**–**13**) were recorded on a Bruker AVANCE NEO 400 M, 500 M, and Bruker AVANCE III HD 700 MHz spectrometer, equipped with a 1.7 mm TCI micro-cryo probe (Bruker Biospin, Rheinstetten, Germany) using NMR tubes of 1.7 mm outer diameter. NMR spectroscopic data of synthetic mediates and compound **11** were collected at Bruker AV-600 MHz (Bruker, Zurich, Switzerland) and tetramethyl chlorosilane (TMS) was as internal standard for chemical shifts. NMR spectra were recorded using MeOH-*d*$_3$ as a solvent. Chemical shifts were referenced to the residual solvent peaks at δ_H 3.31 and δ_C 49.15. Data acquisition and processing were accomplished using TopSpin 3.2 (Bruker, Germany). Standard pulse programs as implemented in TopSpin were used for data acquisition. A Jasco P-1020 polarimeter (Jasco, Tokyo, Japan) was employed to measure optical rotation. CD spectra were obtained using a Chirascan instrument. High-resolution MS and MS/MS spectra for the structure

elucidation were recorded on an ultra-high-performance liquid chromatography–electrospray ionization–quadrupole time-of-flight mass spectrometry system (UHPLC-ESI-MS/Q-TOF-MS, Ultimate 3000 series RSLC (Thermo Dionex, MA, USA coupled to a Bruker tims-TOF mass spectrometer, Bremen, Germany).

Chrysotremulin A (11): Yellow powder (MeOH); $[\alpha]^{21.1}_{D}$ –73.67 (c 0.357, MeOH); UV (MeOH) λ_{max} (logε): 201 (4.19), 228 (4.27), 263 (3.81), 386 (3.68) nm; ^1H and ^{13}C NMR data: see Table S2.1; HRESIMS *m/z* 315.1340 $[M + H]^+$ (calcd for $C_{17}H_{19}N_2O_4$, 315.1339).

Chrysotremulin B (12): Yellow oil (MeOH); $[\alpha]^{21.2}_{D}$ –3.74 (c 0.031, MeOH); UV (MeOH) λ_{max} (logε): 202 (2.85), 223 (2.47) nm; ^1H and ^{13}C NMR data: see Table S2.1; HRESIMS *m/z* 296.0915 $[M + H]^+$ (calcd for $C_{10}H_8NO_3$, 296.0917).

Chrysotremulin C (13): White powder (MeOH); $[\alpha]^{21.2}_{D}$ –1.69 (c 0.039, MeOH); UV (MeOH) λ_{max} (logε): 205 (3.56), 236 (3.31), 279 (2.71), 324 (3.12), 335 (3.13) nm; ^1H and ^{13}C NMR data: see Table S2.1; HRESIMS *m/z* 252.1017 $[M + H]^+$ (calcd for C_9H_8NO, 252.1019).

The positive MS/MS spectrum of compound **11** showed the protonated molecular ion at *m/z* 315.1344 $[M + H]^+$ (calcd. for $C_{17}H_{19}N_2O_4$) with another ion observed at *m/z* 298.0922 $[M – NH_3 + H]^+$ (**fragment 1**) and the characteristic saligenin fragment (**fragment 2**, *m/z*: 107.0493 $[M – H_2O + H – C_{10}H_{12}N_2O_3]^+$). In addition, another prominent ion at *m/z* 209.0922 with a loss of [$–C_7H_7O$] moiety, which was identified as a kynurenine fragment (**fragment 3**) (3). Moreover, the HRESIMS of **11** showed the molecular formula of $C_{17}H_{18}N_2O_4$, suggesting that **11** could be a conjugate consisting of a kynurenine and a saligenin fragment. We also concluded the MS/MS fragmentation of compound **11**. The ion peak at *m/z* 192.0655 with a loss of the [–$C_7H_7O – NH_3$] moiety was corresponding to the **fragment 4**. The ion peak (*m/z* 174.0551) of **fragment 5** was produced through losing a molecular H_2O from **fragment 4**, whereas the loss of [–COOH] of **fragment 4** led to the formation of **fragment 6** (*m/z* 146.0600). The **fragment 7** was a cleavage product of **fragment 6** by the loss of [$–C_3HO$] (Figure S2.1).

The analysis of the negative MS/MS spectrum of **11** exhibited a deprotonated molecular ion at *m/z* 313.1192 $[M – H]^–$ and a fragment ion peak at *m/z* 296.0927 $[M – NH_3 – H]^–$ (**fragment 8**), and *m/z* 252.1032 with a loss [$–COOH – NH_3$] (**fragment 9**). Moreover, the observation of other prominent fragment ion peaks at *m/z* 207.0775 with a loss of the [$–C_7H_7O$] (**fragment 10**), *m/z* 190.0510 $[M – C_7H_7O – NH_3]^–$ (**fragment 11**), and *m/z* 146.0611 $[M – C_7H_7O – NH_3 – CO_2]^–$ (**fragment 12**) further confirmed the structure of compound **11**

(Figure S2.1).

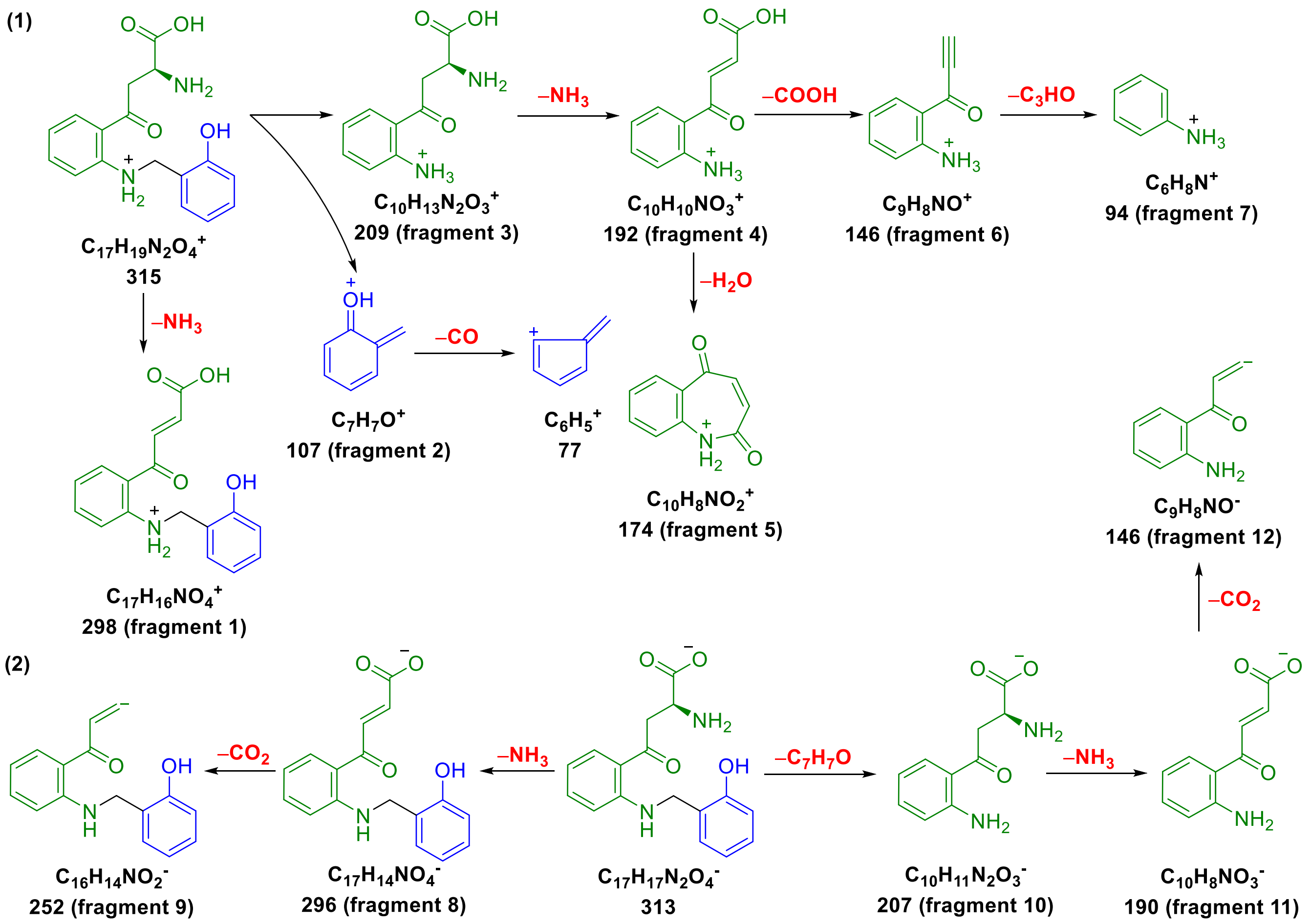

Figure S2.1 MS2 fragmentation of the novel compound chrysotremulin A (**11**) in positive (**1**) and negative (**2**) mode.

Table S2.1 ^{1}H and ^{13}C NMR spectroscopic data of compounds **11–13** (MeOH-d_3) extracted from feces of *C. tremulae* beetles.

Position	11[a]		11[b]		12[c]		13[c]	
	δ_H (J in Hz)	δ_C	δ_H (J in Hz)	δ_C	δ_H (J in Hz)	δ_C	δ_H (J in Hz)	δ_C
1		151.2 C		152.4 C				
2		116.4 C		117.0 C		140.1 C	8.21, d (7.5)	146.9 CH
3	7.82, d (7.8)	131.4 CH	7.83, d (7.7)	132.6 CH	6.42, s	106.0 CH	6.36, d (7.5)	109.2 CH
4	6.62, t (6.4)	114.1 CH	6.59, t (7.3)	115.4 CH		178.7 C		179.9 C
5	7.34, t (7.7)	135.3 CH	7.33, t (7.6)	136.7 CH	8.30, d (7.0)	124.9 CH	8.33, d (8.1)	126.6 CH
6	6.80, overlapped	112.1 CH	6.70, overlapped	113.1 CH	7.38, t (7.0)	123.5 CH	7.42, t (7.5)	125.1 CH
7		-		199.7 C	7.59, overlapped	132.0 CH	7.69, t (7.2)	133.4 CH
8	-	-	3.72, m	40.2 CH_2	7.61, overlapped	117.6 CH	7.76, d (8.7)	118.3 CH
9	-	-	4.02, br s	51.6 CH		126.3 C		127.2 C
10		-		174.3 C		156.5 C		141.1 C
11						162.0 C		
1′		155.0 C		156.3 C		154.2 C		156.1 C
2′		124.6 C		125.7 C		122.3 C		122.4 C
3′	7.18, d (7.0)	128.2 CH	7.11, d (7.3)	129.0 CH	6.84, d (7.2)	126.9 CH	6.92, d (7.6)	129.0 CH
4′	6.70, d (6.7)	119.1 CH	6.72, overlapped	119.9 CH	6.65, t (6.7)	118.9 CH	6.74, t (7.4)	120.5 CH
5′	7.09, t (7.5)	127.8 CH	7.06, t (7.3)	129.0 CH	7.03, t (6.7)	128.1 CH	7.12, t (7.6)	130.3 CH
6′	6.81, overlapped	114.6 CH	6.77, t (7.9)	115.7 CH	6.82, d (7.6)	114.9 CH	6.85, d (8.1)	116.0 CH
7′	4.43, s	41.0 CH_2	4.42, d (5.1)	41.9 CH_2	5.66, s	47.6 CH_2	5.49, s	52.7 CH_2
1′-OH			9.67, s					
7′-NH-1			9.27, s					
9-NH_2			8.09, br s					

[a] recorded at 400/100 MHz (298 K); [b] recorded at 500/125 MHz (234 K); [c] recorded at 700/175 MHz. "-": no detected

3. Synthesis of chrysotremulin A (11)

(1) Boc-protection of an aliphatic amine in L-kynurenine

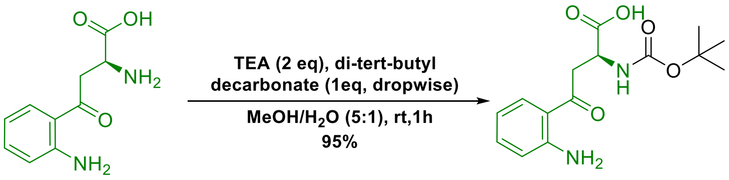

L-kynurenine 14

A solution of L-kynurenine (35 mg, 0.168 mmol) and triethylamine (TEA, 2 equivalent) in MeOH/H_2O (5:1) was stirred at 25ºC. Then, a solution of di-tert-butyl decarbonate (1 equiv) in MeOH/H_2O (5:1) was added dropwise to the mixture. The reaction was stirred at 25ºC for a further 2 h. After completing the reaction, preparative-TLC ($CHCl_3$:MeOH = 20:1 containing 0.1% formic acid, v/v) was used to purify product and 33 mg compound **14** was obtained.

Compound **14**: yellow oil; ^{1}H NMR (600 MHz, CD_3OD): δ_H 7.73 (d, J = 8.0 Hz, 1H), 7.22 (t, J = 7.6 Hz, 1H), 6.72 (d, J = 8.3 Hz, 1H), 6.57 (t, J = 7.6 Hz, 1H), 4.59 (s, 1H), 3.56-3.53 (m, 1H), 3.45-3.38 (m, 1H), 1.42 (s, 9H); ^{13}C NMR (150 MHz, CD_3OD): δ_C 200.4 (C-7), 176.1 (C-10), 157.1 (C-1″), 153.1 (C-1), 135.3 (C-5), 131.6 (C-3), 118.3 (C-4), 118.2 (C-2), 116.2 (C-6), 80.6 (C-2″), 51.3 (C-9), 42.0 (C-8), 28.6 (C-3″,4″,5″); ESI-MS: m/z 309 $[M + H]^+$.

(2) The acetalization of 14 with salicylaldehyde and reduction of aromatic amine

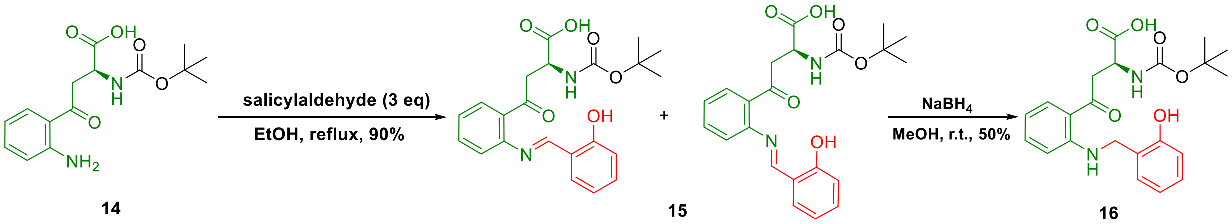

14 15 16

Salicylaldehyde (3 equiv) was added slowly to a solution of **14** (1 equiv) in EtOH (1.5 mL), and the mixture was stirred at 87 ºC for 10 h. The EtOH in the solution was evaporated and the MeOH (2 mL) was used to resolve above samples. After that, $NaBH_4$ (5 equiv) was added in small portions while stirring. When the reduction was complete (2 h), the mixture was directly purified using semi-preparative HPLC (Agilent SB C18, 250 × 9.6 mm, 5 μm; CH_3CN:H_2O = 35% → 74% containing 0.1% formic acid, 20 min, flow rate: 3 mL/min) and 10 mg **16** was yielded (t_R = 16.74 min).

Compound **16**: yellow oil; ^{1}H NMR (600 MHz, CD_3OD): δ_H 7.82 (d, J = 7.8 Hz, 1H), 7.29 (t, J = 7.6 Hz,

1H), 7.15 (d, J = 7.3 Hz, 1H), 7.06 (t, J = 7.3 Hz, 1H), 6.78 (d, J = 7.8 Hz, 1H), 6.74 (t, J = 7.4 Hz, 1H), 6.57 (t, J = 7.4 Hz, 1H), 4.56 (s, 1H), 4.41 (s, 2H), 3.60-3.56 (m, 1H), 3.48-3.42 (m, 1H), 1.41 (s, 9H); ^{13}C NMR (150 MHz, CD_3OD): δ_C 200.7 (C-7), 176.4 (C-10), 157.8 (C-1″), 156.3 (C-1'), 152.5 (C-1), 136.2 (C-5), 132.9 (C-3), 129.4 (C-3'), 129.1 (C-5'), 126.1 (C-2'), 120.5 (C-4'), 118.4 (C-2), 115.9 (C-6'), 115.3 (C-4), 113.3 (C-6), 80.5 (C-2"), 51.3 (C-9), 42.3 (C-8), 42.2 (C-7'), 28.7 (C-3",4",5"); ESI-MS: m/z 415 $[M + H]^+$.

(3) Boc-deprotection of compound 16

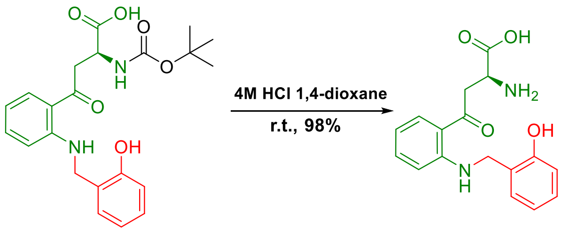

16 **Syn-11**

Compound **16** (10 mg) was dissolved in a mixture of 4M HCl dioxane and stirred at room temperature for 2 h. After reaction, the mixture was neutralized with $NaHCO_3$ (aq) and the solvent was removed. Subsequently, the solid was washed using MeOH and further purified by HPLC (Agilent SB C18, 250 × 9.6 mm, 5 μm; CH_3CN:H_2O = 32% containing 0.1% formic acid, 8 min, flow rate: 3 mL/min) to get syn-**11** (5.8 mg, t_R = 6.2 min).

Syn-**11**: yellow solid; $[\alpha]^{25}_D$ –11.27° (c 0.11, MeOH); CD (MeOH) Δε213 0.32, Δε231 +1.57, Δε265 0.02, Δε381 – 0.22; ^{1}H NMR (600 MHz, CD_3OD): δ_H 7.81 (d, J = 7.8 Hz, 1H), 7.34 (t, J = 7.6 Hz, 1H), 7.16 (d, J = 7.3 Hz, 1H), 7.07 (t, J = 7.3 Hz, 1H), 6.80 (d, J = 7.8 Hz, 1H), 6.74 (t, J = 7.4 Hz, 1H), 6.60 (t, J = 7.4 Hz, 1H), 4.42 (s, 2H), 4.00 (s, 1H), 3.75-3.73 (m, 1H), 3.64-3.60 (m, 1H); ^{13}C NMR (150 MHz, CD_3OD): δ_C 200.2 (C-7), 174.3 (C-10), 156.4 (C-1'), 152.6 (C-1), 136.6 (C-5), 132.7 (C-3), 129.6 (C-3'), 129.3 (C-5'), 125.9 (C-2'), 120.4 (C-4'), 117.4 (C-2), 116.0 (C-6'), 115.4 (C-4), 113.5 (C-6), 51.9 (C-9), 42.3 (C-8), 42.3 (C-7'); HR-ESI-MS: m/z 315.1341 $[M + H]^+$, calcd for $C_{17}H_{19}N_2O_4$, m/z 315.1339 $[M + H]^+$.

4. Structural elucidation of compounds 11–13

The molecular formula of compound **11** was determined to be $C_{17}H_{18}N_2O_4$ on the basis of HRESIMS spectrum (*m/z* 315.1344 $[M + H]^+$, calcd. 315. 1346 $[M + H]^+$). Its 1D NMR spectroscopic data (Table S2.1) showed two di-substituted benzyl groups, which were assigned as eight aromatic methine signals at δ_H 7.82 (d, *J* = 7.8 Hz, H-3), δ_C 131.4, C-3; δ_H 6.62 (t, *J* = 6.4 Hz, H-4), δ_C 114.1, C-4; δ_H 7.34 (t, *J* = 7.7 Hz, H-5), δ_C 135.2, C-5; δ_H 6.80 (overlapped, H-6), δ_C 112.1, C-6; δ_H 6.81 (overlapped, H-3′), δ_C 114.6, C-3′; δ_H 7.09 (t, *J* = 7.5 Hz, H-4′), δ_C 127.8, C-4′; δ_H 6.70 (t, *J* = 6.7 Hz, H-5′), δ_C 119.1, C-5′ and δ_H 7.18 (d, *J* = 7.0 Hz, H-6′), δ_C 128.2, C-6′, as well as four sp^2 quaternary carbon signals at δ_C 151.2 (C-1), δ_C 116.4 (C-2), δ_C 124.6 (C-1′), and δ_C 155.0 (C-2′). In addition, only a methylene [δ_H 4.43 (s, H-7′), δ_C 41.0 (C-7′)] was observed in 1D NMR spectra. The observed NMR spectral features exhibited discrepancies with the structural assignment of compound **11** derived from LC-HRMS data. We posited that dynamic broadening or signal loss in the C-7/C-8/C-9/C-10 fragment arose from unrestricted rotation around the single bond (4). To validate this hypothesis, variable-temperature NMR experiments were performed to constrain bond rotation. Strikingly, under low-temperature conditions (500/125 MHz; 234 K), the diagnostic signals corresponding to this fragment became resolvable in the 1D NMR spectra (Figures S2.18–S2.19). Under these conditions, two methylene signals at δ_H 4.42 (d, *J* =5.1 Hz, H-7′), δ_C 41.9 (C-7′), and δ_H 3.60 (t, *J* = 6.4 Hz, H-8), δ_C 38.9 (C-8), one methine containing nitrogen (δ_H 4.02, br s, H-9; δ_C 51.6, C-9), one ketone (δ_C 199.7, C-7), and one ester group (δ_C 174.3, C-10) were observed.

Furthermore, the Heteronuclear Multiple Bond Correlation (HMBC) spectrum of **11** showed the correlations of H-8 with C-7, C-9, and C-10; of H-3 with C-1, C-2, C-5, and C-7; of 9-NH_2 (δ_H 8.09, br s) with C-9; of H-7′ with C-1, C-1′, C-2′, and C-3′; of 1′-OH with C-1′, C-2′, and C-6′; of H-6′ with C-1′, C-2′, and C-4′; of 7′-NH-1 with C-1, C-2′, C-7′, and C-6 (**Figure S2.2**). The ^1H-^1H Correlation spectroscopy (COSY) correlations (**Figure S2.2**) of H-3/H-4/H-5/H-6, of H-8/H-9/9-NH_2, of H-3′/H-4′/H-5′/H-6′, and of H-7′/7′-NH were also observed. These measurements confirmed the basic structure of **11** without stereochemistry. However, the extended aliphatic chain precluded unambiguous determination of the C-9 absolute configuration *via* computational ECD methods due to conformational flexibility interfering with chiroptical predictions.

The structural architecture of compound **11** comprises two distinct moieties: a saligenin unit and a

kynurenine-derived fragment, with the latter biosynthetically originating from L-tryptophan via the kynurenine pathway (5, 6). Given the natural occurrence of L-tryptophan, we hypothesized an *S*-configuration at C-9 in **11**. To definitively resolve the absolute stereochemistry, we designed a stereocontrolled synthesis of **11** using L-kynurenine (retaining its native configuration) and salicylaldehyde as building blocks. Exploiting the heightened nucleophilicity of the aliphatic amine at C-9 relative to the aromatic amine at C-1 in L-kynurenine, we selectively installed a Boc (tert-butyloxycarbonyl) protecting group on the C-9 amine under standard coupling conditions (7), yielding intermediate **14**. Subsequent Schiff base formation via acetalization between the C-1 aromatic amine of **14** and salicylaldehyde generated imine **15**. Without isolation, **15** was subjected to in situ $NaBH_4$ reduction in a one-pot procedure to afford secondary amine **6**.(8) Acidic cleavage of the Boc group in **16** furnished **syn-11** (9), whose stereochemical identity was corroborated through comparative CD curves and spectroscopic analyses with **iso-11** (Figure S2.41–S2.48). Thus, the absolute configuration of C-9 in compound **11** was established to be *S*.

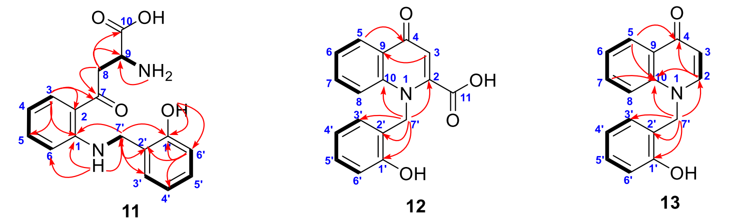

11 **12** **13**

Figure S2.2. Selected HMBC and ^{1}H-^{1}H COSY correlations of compounds **11-13**.

Compound **12** has a molecular formula $C_{17}H_{13}NO_4$ deduced by HRESIMS spectrum (*m/z* 296.0915 $[M + H]^+$, calcd. 296.0917 $[M + H]^+$), indicating 12 degrees of unsaturation. The ^{1}H NMR spectroscopic data (Table S2.1) of **12** showed two group aromatic signals at δ_H 8.30 (d, *J* = 7.0 Hz, H-5), 7.38 (d, *J* = 7.0 Hz, H-6), 7.59 (overlapped, H-7), 7.61 (overlapped, H-8); δ_H 6.84 (d, *J* = 7.2 Hz, H-3′), 6.65 (d, *J* = 6.7 Hz, H-4′), 7.03 (d, *J* = 6.7 Hz, H-5′), 6.85 (d, *J* = 7.6 Hz, H-6′), which were contributable to two characteristic di-substituted phenyls with AB pattern based on HSQC and ^{1}H-^{1}H COSY spectra. In addition, one methylene proton at δ_H 5.66 (s, H-7′) and one singlet *sp*^{2} proton at δ_H 6.42 (s, H-3) were observed in its ^{1}H NMR spectrum. Furthermore, analysis of HMBC spectrum displayed the correlations of H-7′ with C-1′ (δ_C 154.2), C-2′ (δ_C 122.3), and C-3′ (δ_C 126.9), of H-3′ and H-5′ with C-1′, and of H-4′ and H-6′ with C-2′, indicating the presence of *o*-hydroxybenzyl.

Additionally, there were HMBC correlations (Figure S2.2) of H-3 (δ_H 6.42, s) with C-9 (δ_C 126.3) and C-

11 (δ_C 162.0), meanwhile, H-5 showed HMBC correlations with a ketone (δ_C 178.7, C-4) and C-10 (δ_C 156.5). Moreover, H-6 and H-8 correlated with C-9 (δ_C 126.3), which illustrated that a 4-oxo-1,4-dihydroquinoline-2-carboxylic acid fraction is existed in **12**. Notably, H-7′ also showed HMBC correlations with two sp^2 quaternary carbons [C-2 (δ_C 140.1) and C-10 (δ_C 156.5)], which proved that *o*-hydroxybenzyl was connected to kynurenic acid *via* a C-N bond (C-1 and C-2′). Finally, the structure of **12** was determined and named chrysotremulin B (**12**).

Based on the HRESIMS ion at *m/z* 252.1017 [M + H]$^+$ (calcd. 252.1019 [M + H]$^+$), the molecular formula of **13** was established to be $C_{16}H_{13}NO_2$. The 1D NMR spectroscopic data (Table S2.1) of **13** showed high similarities with those of **12** with only differences on the absence of the carboxyl and the replacement of the sp^2 quaternary carbon by a sp^2 methine (δ_H 8.21, d, J = 7.5 Hz, H-2; δ_C 146.9, C-2) in **13**. The further HMBC correlations (Figure S2.2) of H-2 with C-10 (δ_C 141.1), C-7′ (δ_C 52.7), and C-4 (δ_C 179.9), as well as ^{1}H-^{1}H COSY correlation of H-2/H-3 suggesting the presence of quinolin-4(1*H*)-one moiety in **13** because of decarboxylation at C-2 in **12**. Therefore, the structure of **13** was established and named as chrysotremulin C (**13**).

5. Analysis of L-tryptophan, L-kynurenine, kynurenic acid, and 4-hydroxy-quinoline in *C. tremulae* feces

For the preparation of the standard solution, 1 mg L-tryptophan (ROTH, 135201610), L-kynurenine (Aldrich, K8625), kynurenic acid (Fluka, 61260), and 4-hydroxy-quinoline (SIGMA-ALDRICH, H58005-1G) were put into 1.5 mL tubes, and 1 mL MeOH was added to prepare a 1 mg/mL solution. Then, standard solution was diluted to 10 µg/mL, which was subjected to UHPLC-HRMS (Bruker timsTOF mass spectrometer, Bremen, Germany) analysis, using the chromatographic system described in supporting information-1.

6. Analysis of salicortin and its metabolites in other 23 poplar herbivore species

To determine whether salicortin and its metabolites also occur in other poplar herbivores, feces of 23 different insect species (Table S2.2) were homogenized and extracted as described above. For the feces collection in different insect herbivore species, individuals were collected from the field and fed with *P. nigra* leaves in the lab. The feces were then stored at –20 ºC until extraction. 10 mg feces from each species were extracted with 500 µL aqueous MeOH (50%, v:v) and qualitatively analyzed for salicortin, salicortinol, salicin, saligenin, salicylic acid, salicortin phosphate, salicin phosphate, quinic acid esters by UHPLC-HRMS using

the methods described above.

7. Quantification of kynuerenine, kynurenic acid, 4-hydroxyquinoline, chrysotremulin A by LC-MS/MS

For quantitative analysis of kynurenine, kynurenic acid, 4-hydroxyquinoline, chrysotremulin A (**11**) in feces from 23 poplar herbivores (Table S2.3), above feces extracts (20 mg/mL) were diluted to 2 mg/mL using extraction solvent. A series of concentrations (10, 2, 1, 0.5, 0.25, and 0.125 μg/mL) of a standards mixture were prepared, in which the concentrations of kynurenine, kynurenic acid, and 4-hydroxyquinoline were 2.35, 0.47, 0.235, 0.118, 0.059, and 0.029 μg/mL, while the concentrations of chrysotremulin A (**11**) were 2.89, 0.578, 0.289, 0.145, 0.072, and 0.036 μg/mL.

Analytes were chromatographed on an Agilent 1200 series HPLC-MS/MS system equipped with a reverse phase (RP) ZORBAX Eclipse XDB-C-18 column (4.6 × 50 mm, 1.8 μm, Agilent Technologies, Santa Clara, USA). Gradient elution with 0.05% aqueous formic acid (A) and acetonitrile (B) was applied using the following parameters: 5% B (0-6 min), increased to 37.4% (6-6.02 min) and further to 100% (6.02-7 min), held shortly at 100% (7-7.02 min) and was then decreased again to the 5% B (7.02-9.5 min). The injection volume and mobile eluent flowrate amounted to 1 μL and 1.1 mL/min, respectively. Eluted compounds were ionized by electrospray (negative mode) and detected on an QTRAP 6500 mass spectrometer (AB Sciex, Darmstadt, Germany) using multiple reaction monitoring (MRM). MRM parameters for each analyte were optimized using standard compounds, either purchased or isolated from beetle feces as described above under "Isolation and identification". Quantification by external standard curves was carried out. MRM-parameters: (precursor ion *m/z* → product ion *m/z*; decluttering potential [V], collision energy [V]): chrysotremulin A (313 → 146; –55, –24), kynurenine (207 → 190; –35, –12), kynurenic acid (188 → 144; –5, –16), and 4-hydroxyquinoline (144 → 102; –75, –30). Peak integration was carried out using the Analyst 1.6.3 software (AB Sciex, Darmstadt, Germany).

Supplementary figures

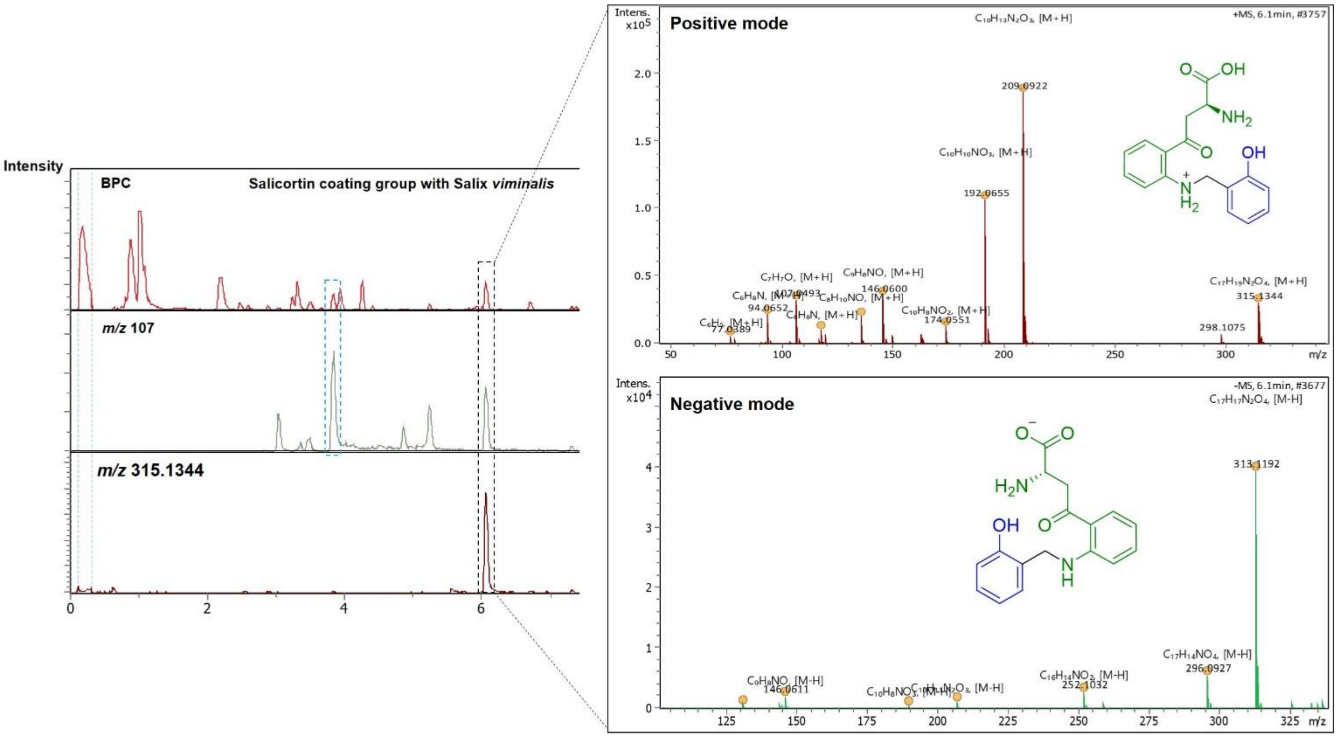

Figure S2.3 Full scan HRMS spectrum of chrysotremulin A (**11**) in the feces extract of the salicortin coating group with *Salix viminalis*. Observed peaks of chrysotremulin A (**11**) at *m/z* 315.1344 $[M + H]^+$ (positive mode) and *m/z* 313.1192 $[M – H]^–$ (negative mode).

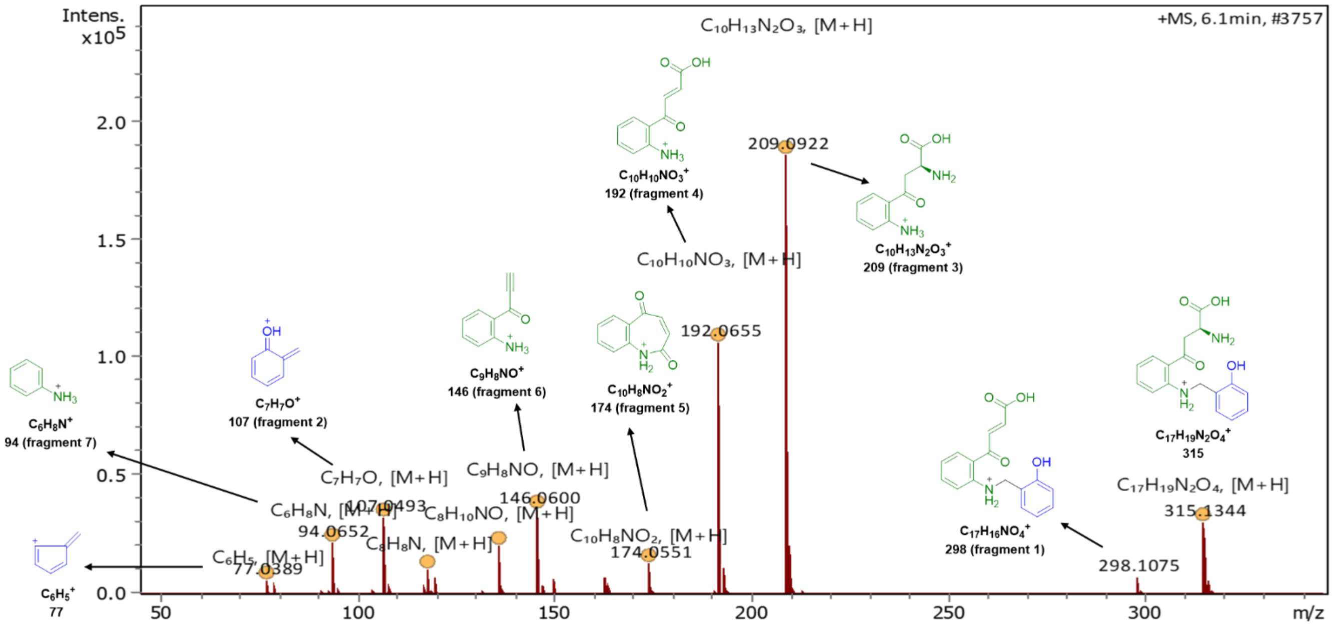

Figure S2.4 MS2 fragmentation of chrysotremulin A (**11**) in the MS spectrum (positive mode) (salicortin coating experiment with *Salix viminalis*).

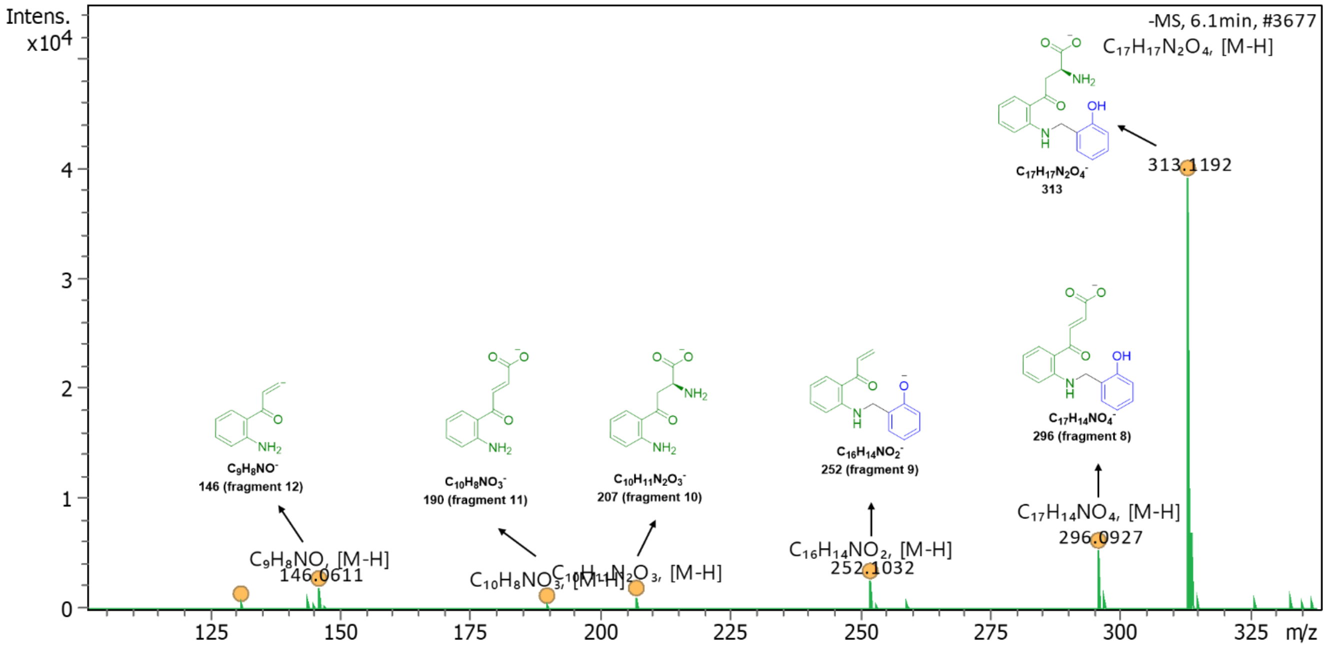

Figure S2.5 MS2 fragmentation of chrysotremulin A (**11**) in the MS spectrum (negative mode) (salicortin coating experiment with *Salix viminalis*).

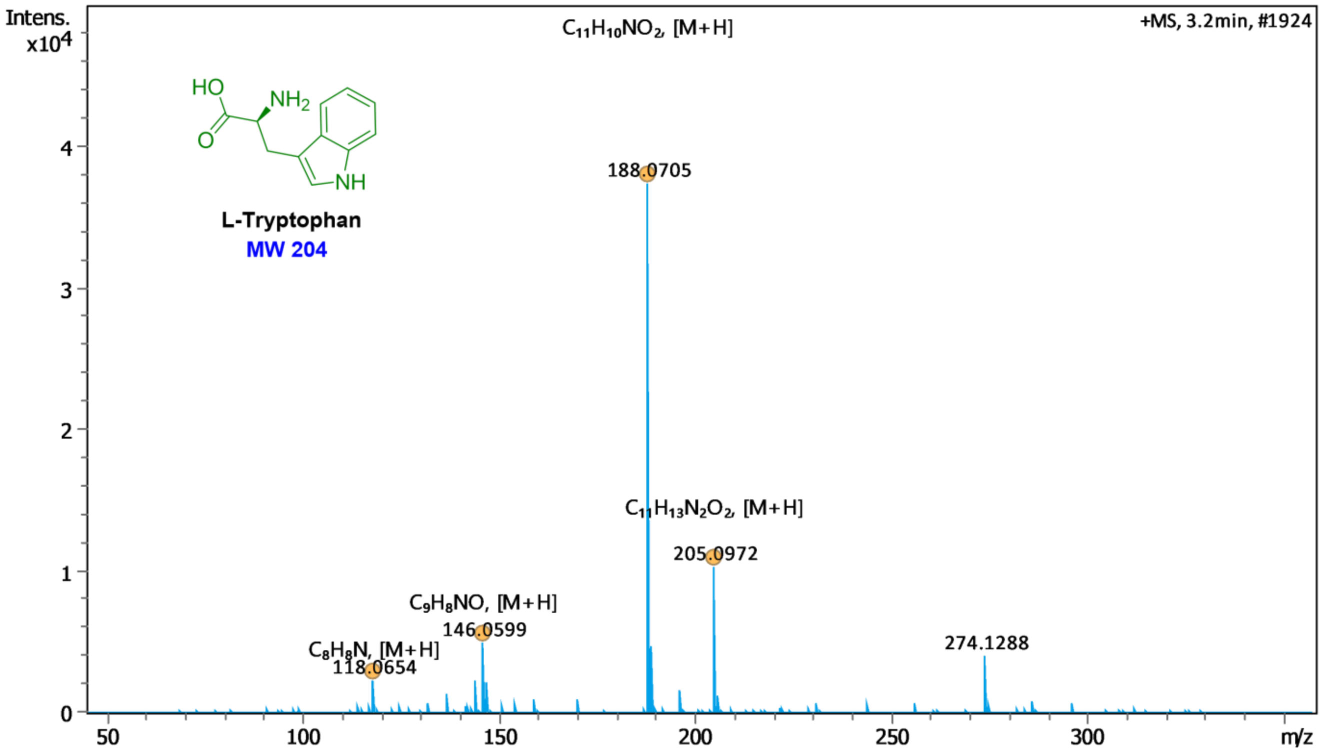

Figure S2.6 Full scan HRMS spectrum (positive mode) of tryptophan in the feces extracts of the salicortin coating group with *Salix viminalis*. Observed peaks of tryptophan at *m/z* 205.0972 $[M + H]^+$ and *m/z* 188.0705 $[M – NH_2 + H]^+$.

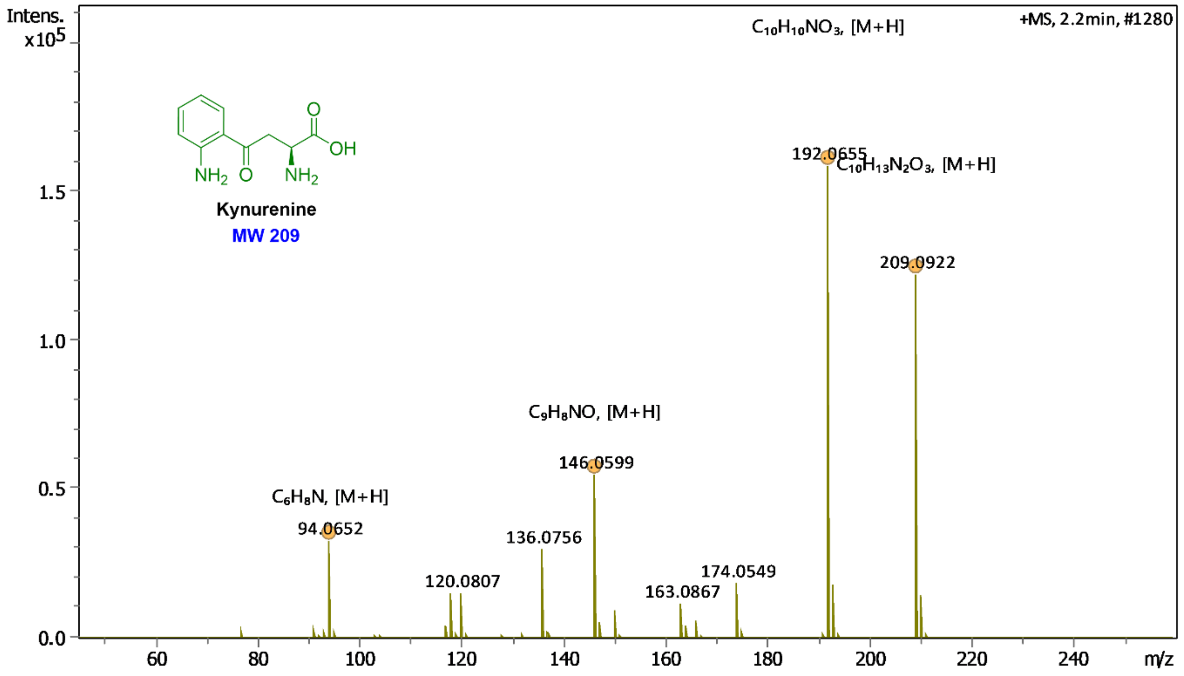

Figure S2.7 Full scan HRMS spectrum (positive mode) of kynurenine in the salicortin coating group with *Salix viminalis*. Observed peaks of kynurenine at *m/z* 209.0922 $[M + H]^+$ and *m/z* 192.0655 $[M – NH_2 + H]^+$.

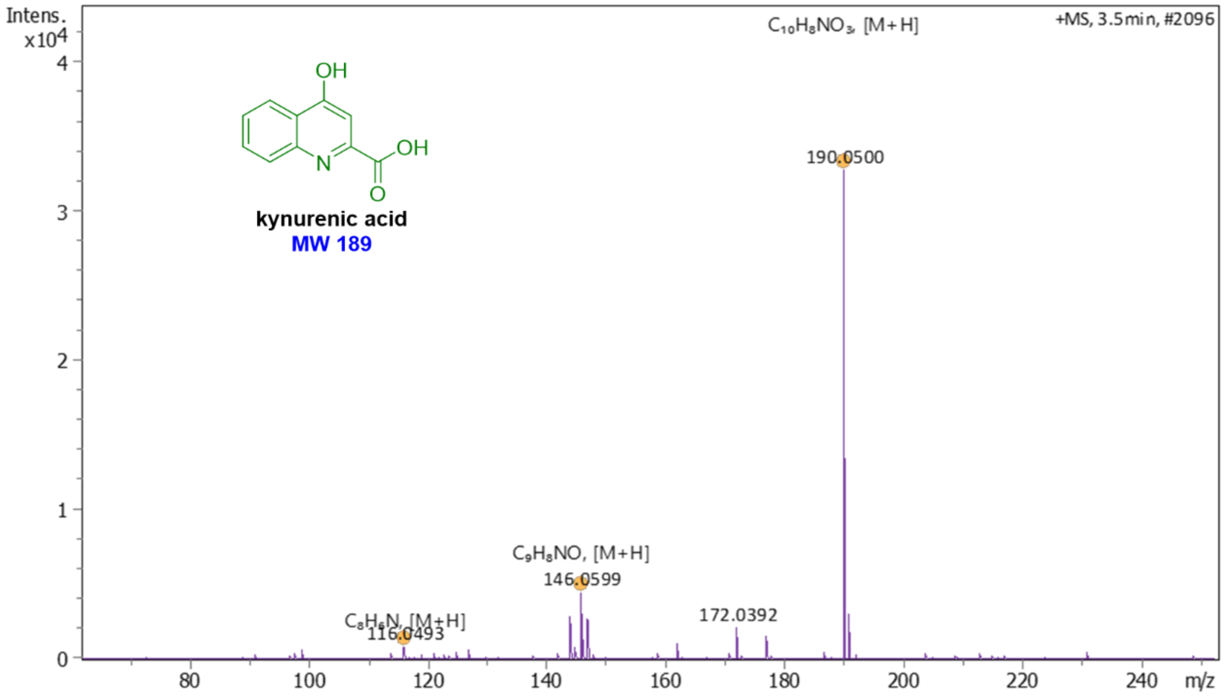

Figure S2.8 Full scan HRMS spectrum (positive mode) of kynurenic acid in the salicortin coating group with *Salix viminalis*. Observed peaks of kynurenic acid at *m/z* 190.0500 $[M + H]^+$ and *m/z* 146.0599 $[M – COOH + H]^+$.

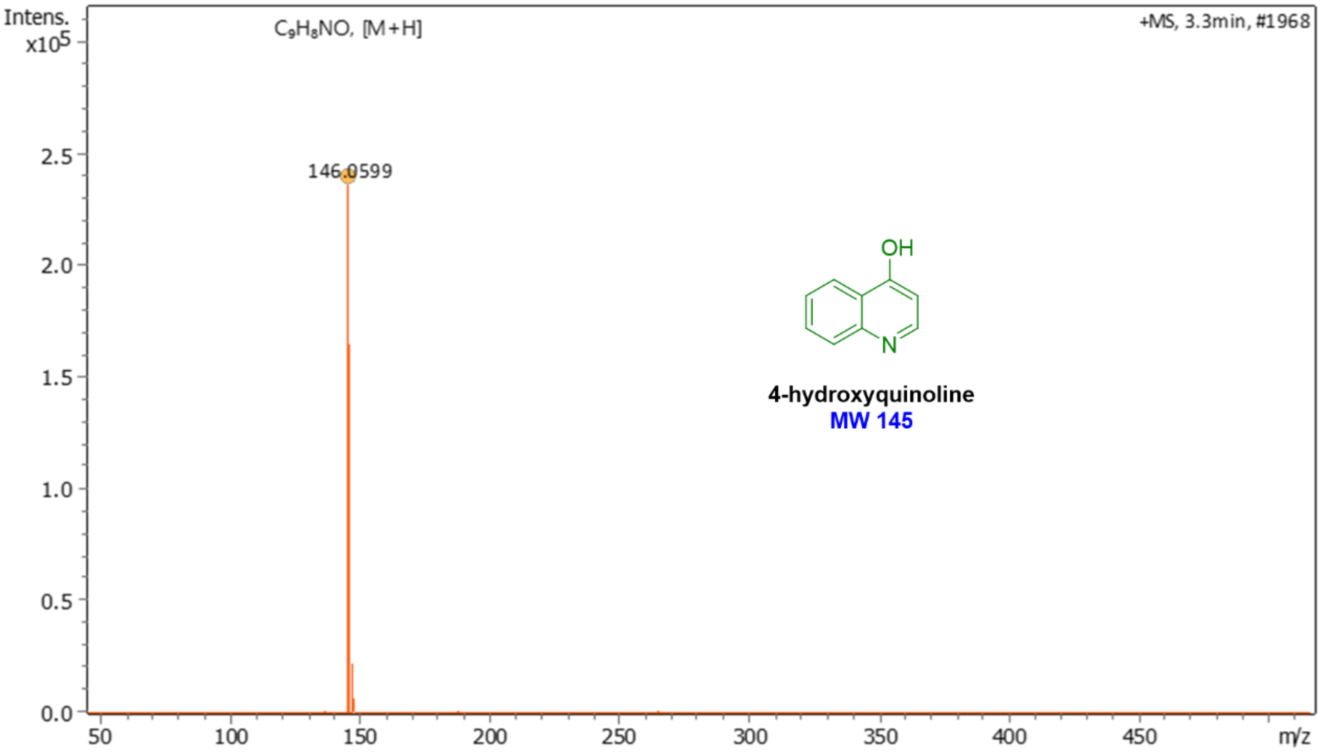

Figure S2.9 Full scan HRMS spectrum (positive mode) of 4-hydroxyquinoline in the salicortin coating group with *Salix viminalis*. Observed peak of 4-hydroxyquinoline at *m/z* 146.0599 $[M + H]^+$.

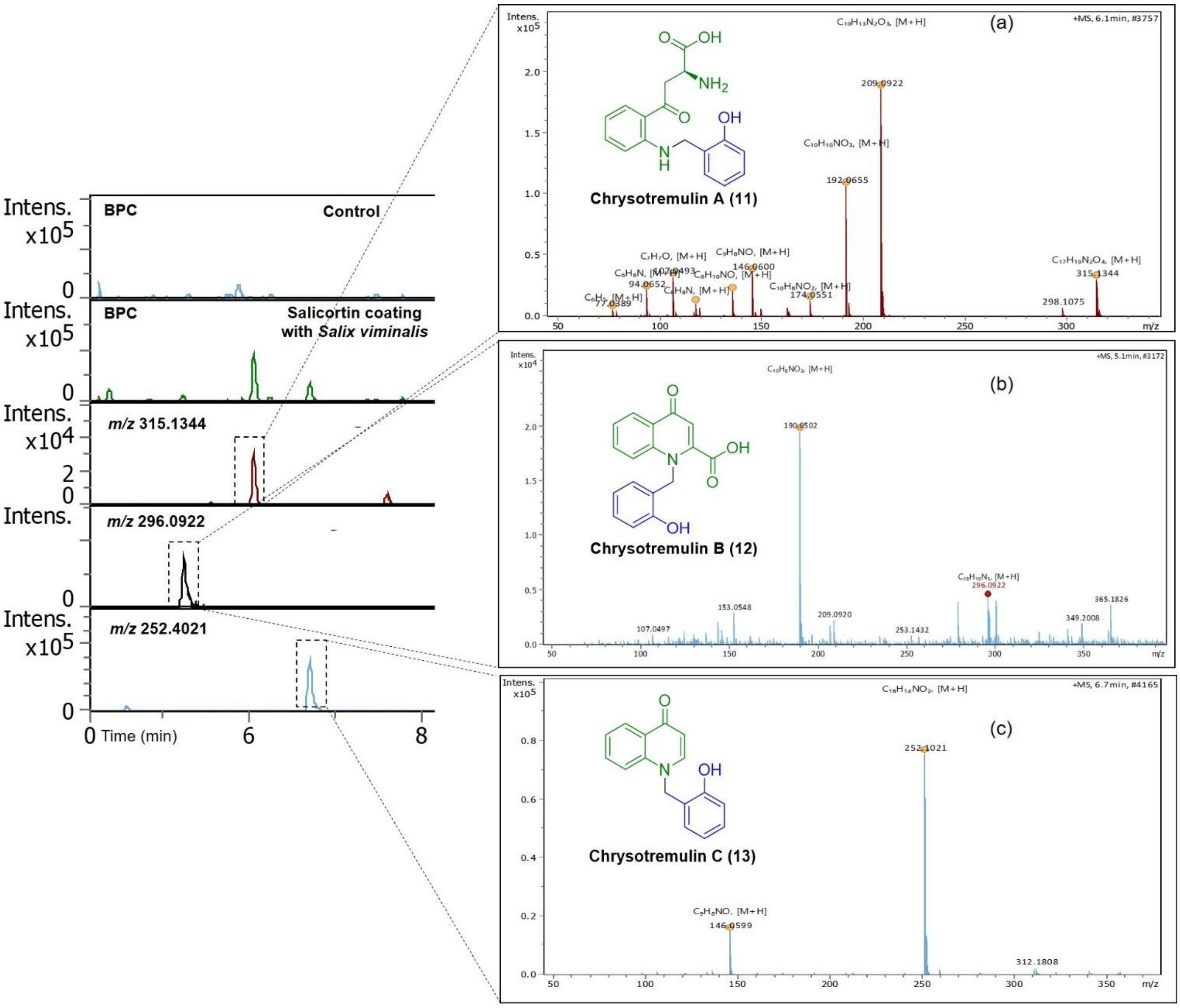

Figure S2.10 Full scan HRMS spectra (positive mode) of (a) chrysotremulin A (**11**), (b) chrysotremulin B (**12**), and (c) chrysotremulin C (**13**) in the feces extract of the salicortin coating group with *Salix viminalis*. Observed peaks of chrysotremulin A (**11**) at *m/z* 315.1344 $[M + H]^+$, chrysotremulin B (**12**) at *m/z* 296.0922 $[M + H]^+$, and chrysotremulin C (**13**) at *m/z* 252.1021 $[M + H]^+$. The left panel shows base peak chromatograms (BPC) and extracted ion chromatograms at *m/z* 315.1344, *m/z* 296.0922, and *m/z* 252.1021 for the feces extracts of the control group and for the salicortin coating group

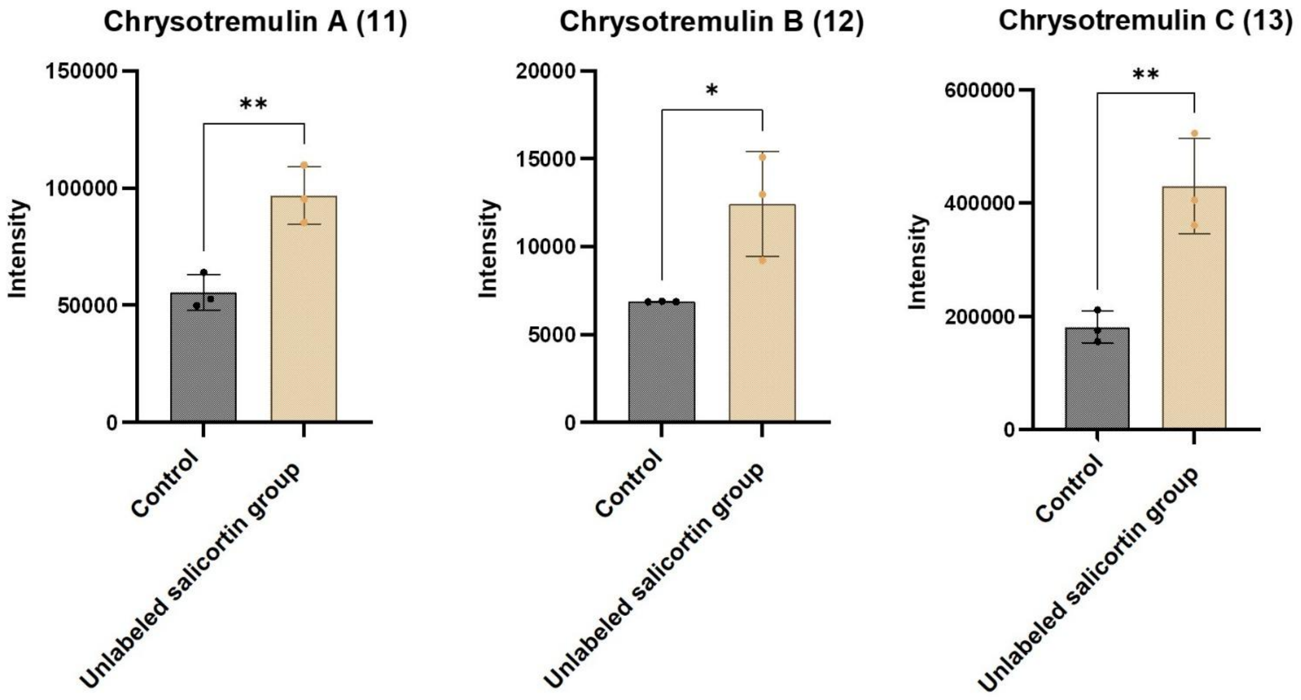

Figure S2.11 The relative contents of chrysotremulins A–C (**11**–**13**) between unlabeled salicortin coating group and control group with *Populus nigra*. The student's t test with *P < 0.05, ** P < 0.01, *** P < 0.005, ****P < 0.001 vs Control. The data are presented as the mean ± SD (n = 3).

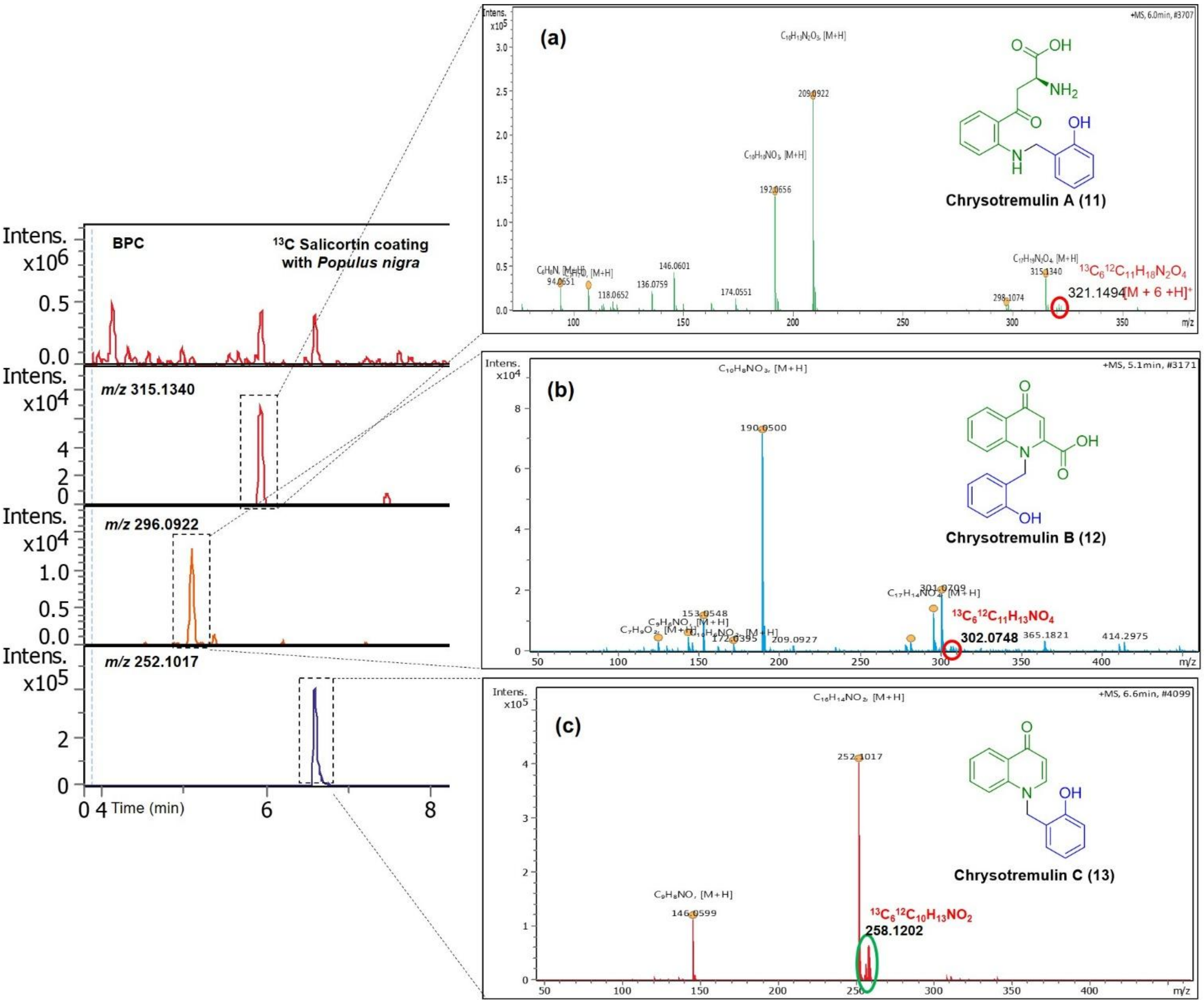

Figure S2.12 Full scan HRMS spectra (positive mode) of (a) chrysotremulin A (**11**), (b) chrysotremulin B (**12**), and (c) chrysotremulin C (**13**) in the feces extracts of the ^{13}C salicortin coating group with *Populus nigra.* Observed peaks of unlabeled chrysotremulin A (**11**) at *m/z* 315.1340 $[M + H]^+$, unlabeled chrysotremulin B (**12**) at *m/z* 296.0922 $[M + H]^+$, unlabeled chrysotremulin C (**13**) at *m/z* 252.1021 $[M + H]^+$, ^{13}C chrysotremulin A (**11**) at *m/z* 321.1494 $[M + 6 + H]^+$, ^{13}C chrysotremulin B (**12**) at *m/z* 302.0748 $[M + 6 + H]^+$, and ^{13}C chrysotremulin C (**13**) at *m/z* 258.1202 $[M + 6 + H]^+$. The left panel shows base peak chromatograms (BPC) and extracted ion chromatograms at *m/z* 315.1192, *m/z* 296.0922, and *m/z* 252.1021 for the feces extracts of the ^{13}C salicortin coating group.

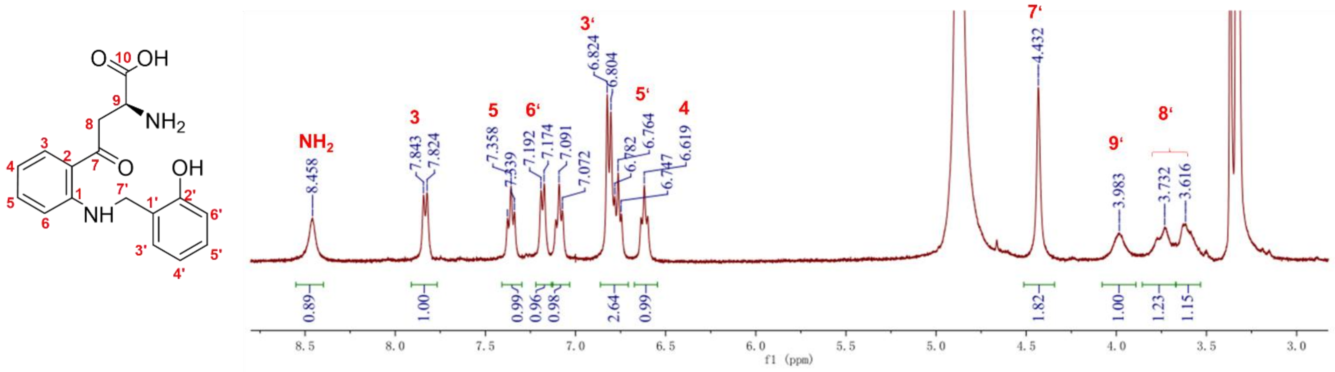

Figure S2.13 ^{1}H NMR (400 MHz, CD_3OH, 298K) spectrum of chrysotremulin A (**11**) from *C. tremulae* feces.

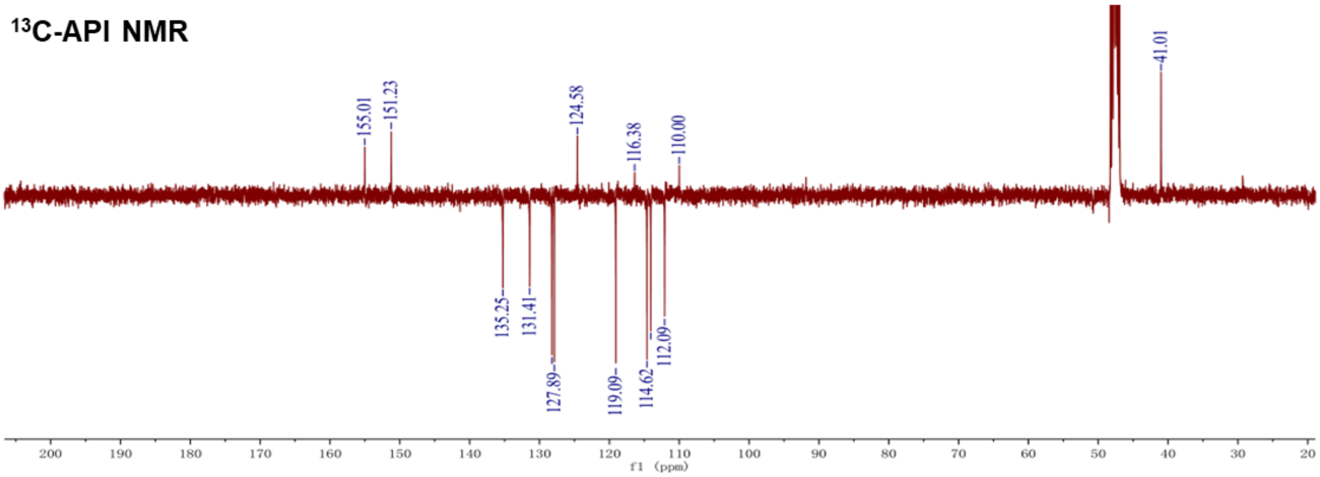

Figure S2.14 ^{13}C-API NMR (100 MHz, CD_3OH, 298K) spectrum of chrysotremulin A (**11**) from *C. tremulae* feces.

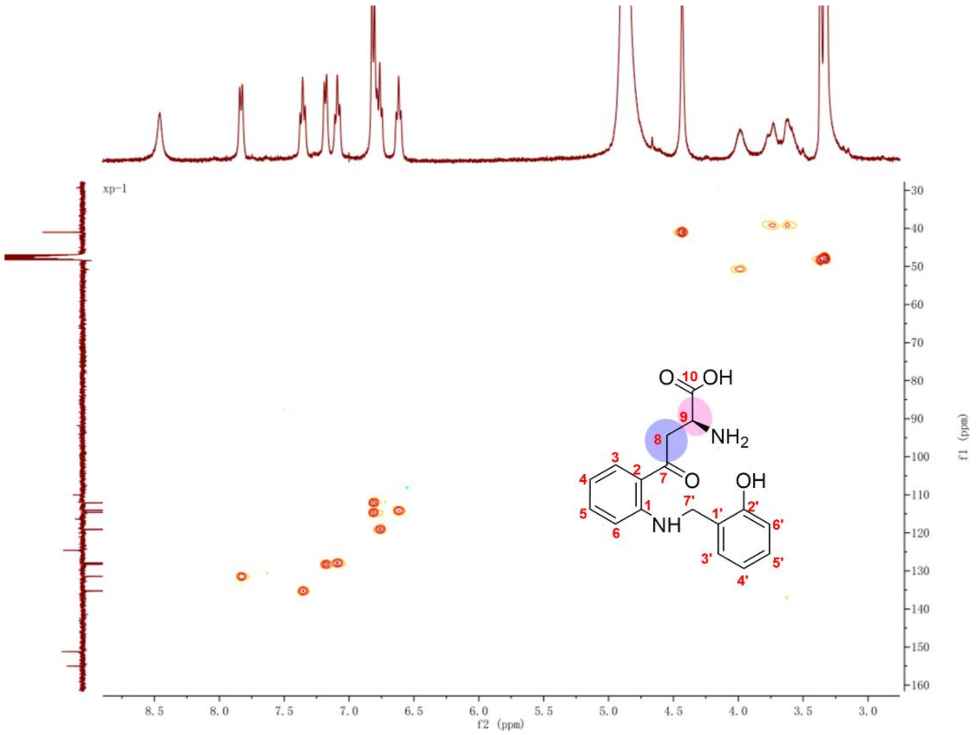

Figure S2.15 HSQC (400/100 MHz, CD_3OH, 298K) spectrum of chrysotremulin A (**11**) from *C. tremulae* feces.

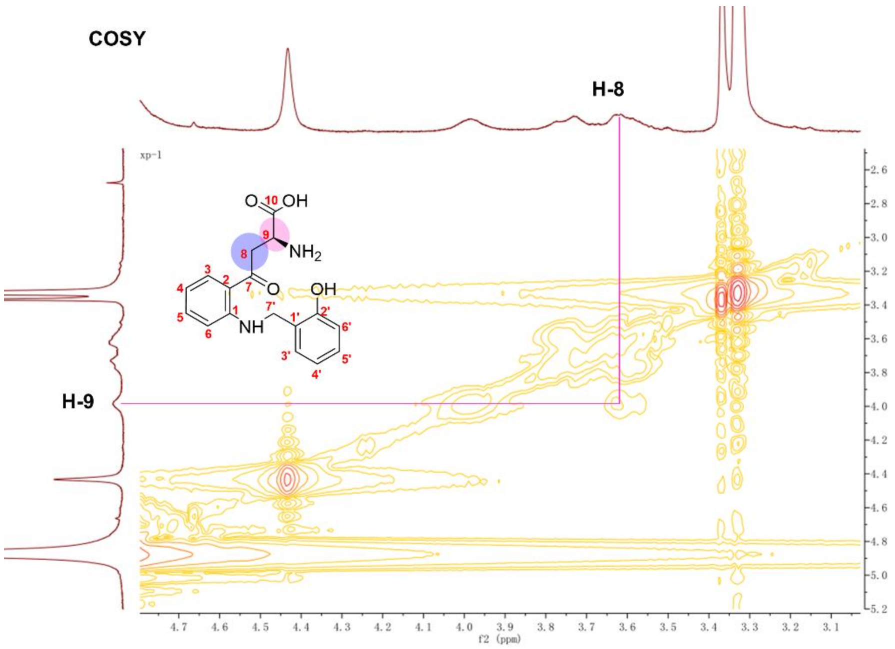

Figure S2.16 ^{1}H-^{1}H COSY (400/100 MHz, CD_3OH, 298K) spectrum of chrysotremulin A (**11**) from *C. tremulae* feces.

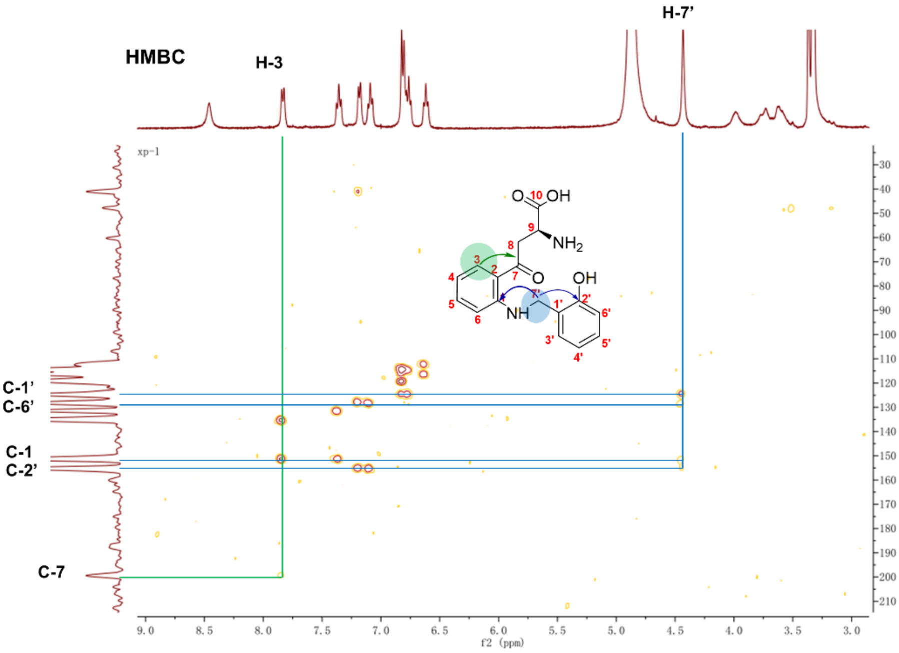

Figure S2.17 HMBC (400/100 MHz, CD_3OH, 298K) spectrum of chrysotremulin A (**11**) from *C. tremulae* feces.

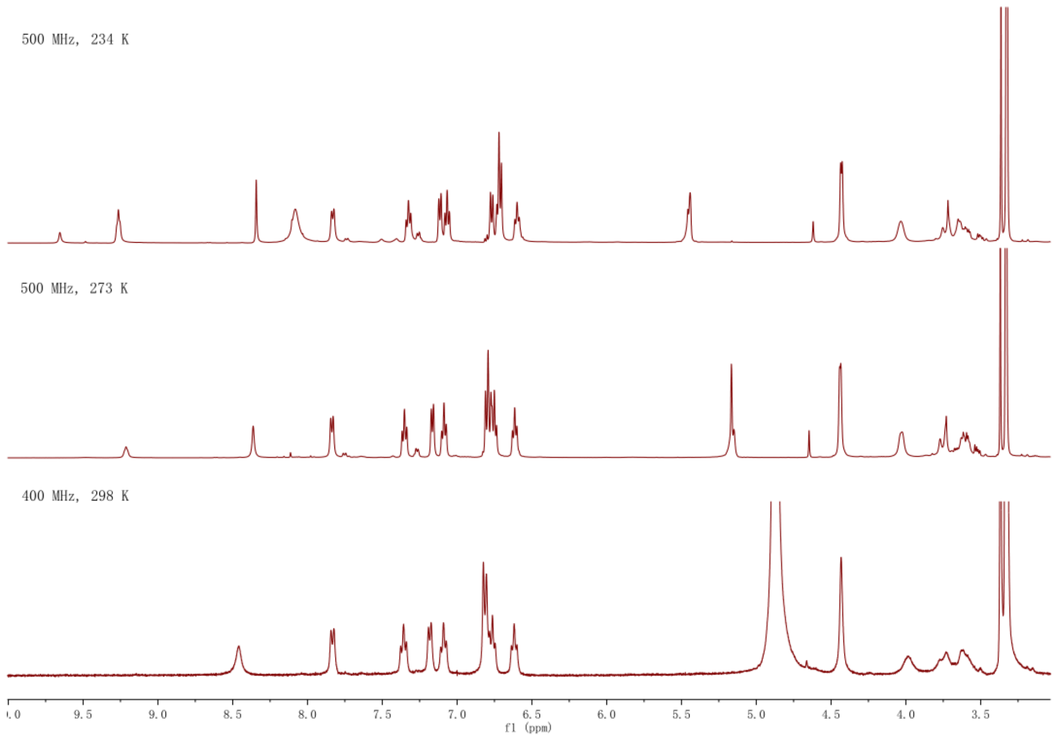

Figure S2.18 Comparison of ^{1}H NMR spectra between 298, 273 and 234 K of chrysotremulin A (**11**) from *C. tremulae* feces.

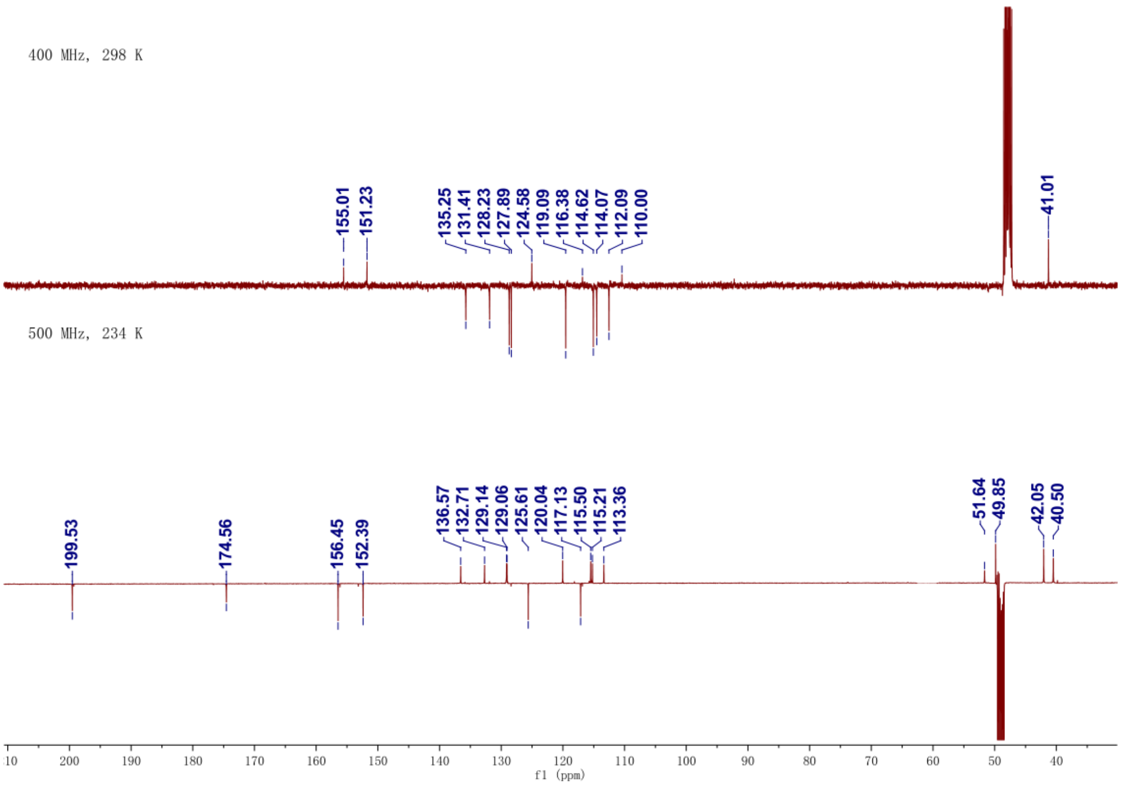

Figure S2.19 Comparison of ^{13}C NMR spectra between 298 K and 234 K chrysotremulin A (**11**) from *C. tremulae* feces.

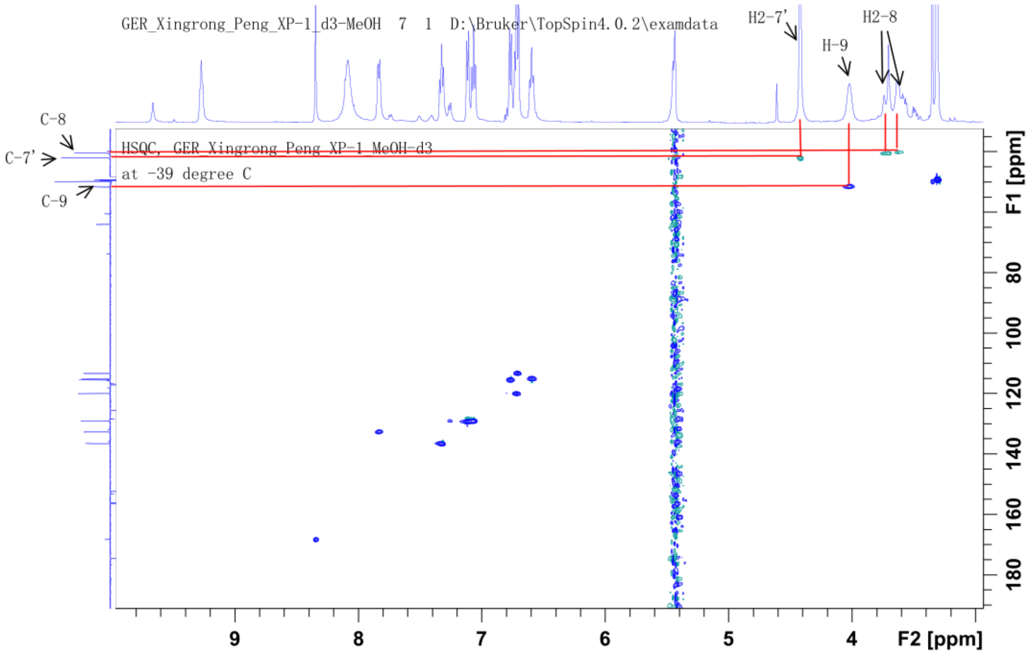

Figure S2.20 HSQC spectrum (234 K) of chrysotremulin A (**11**) from *C. tremulae* feces.

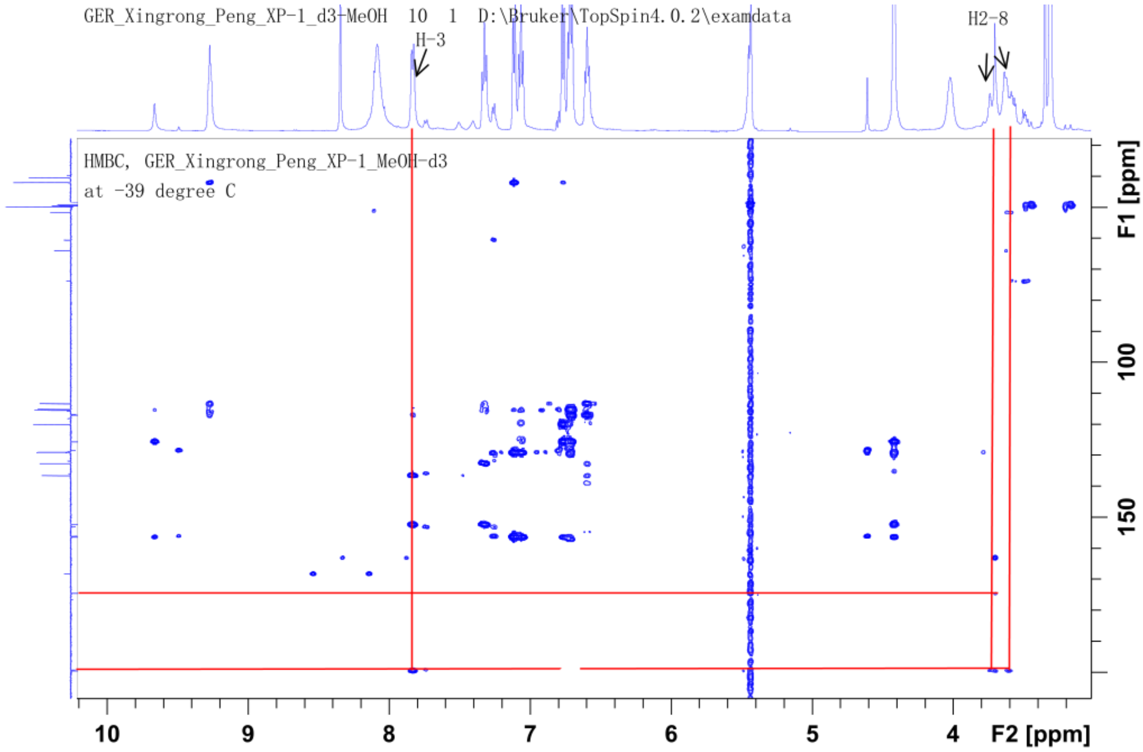

Figure S2.21 HMBC spectrum (234 K) of chrysotremulin A (**11**) from *C. tremulae* feces.

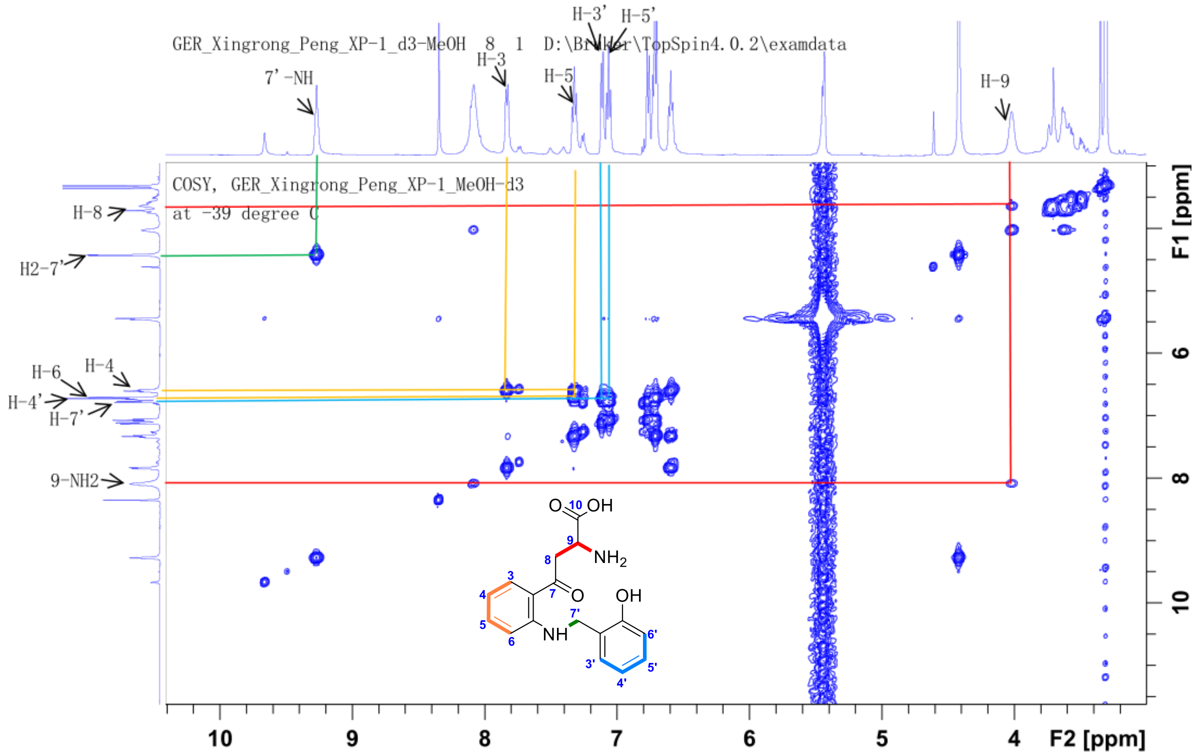

Figure S2.22 ^{1}H-^{1}H COSY spectrum (234 K) of chrysotremulin A (**11**) from *C. tremulae* feces.

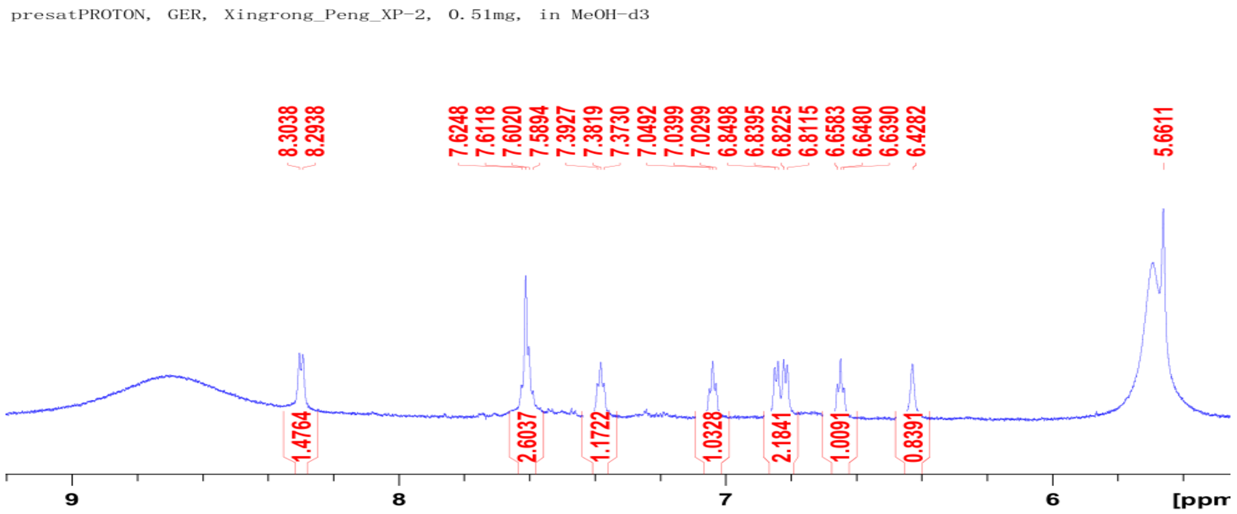

Figure S2.23 ^{1}H NMR spectrum (700 MHz) of chrysotremulin B (**12**) from *C. tremulae* feces.

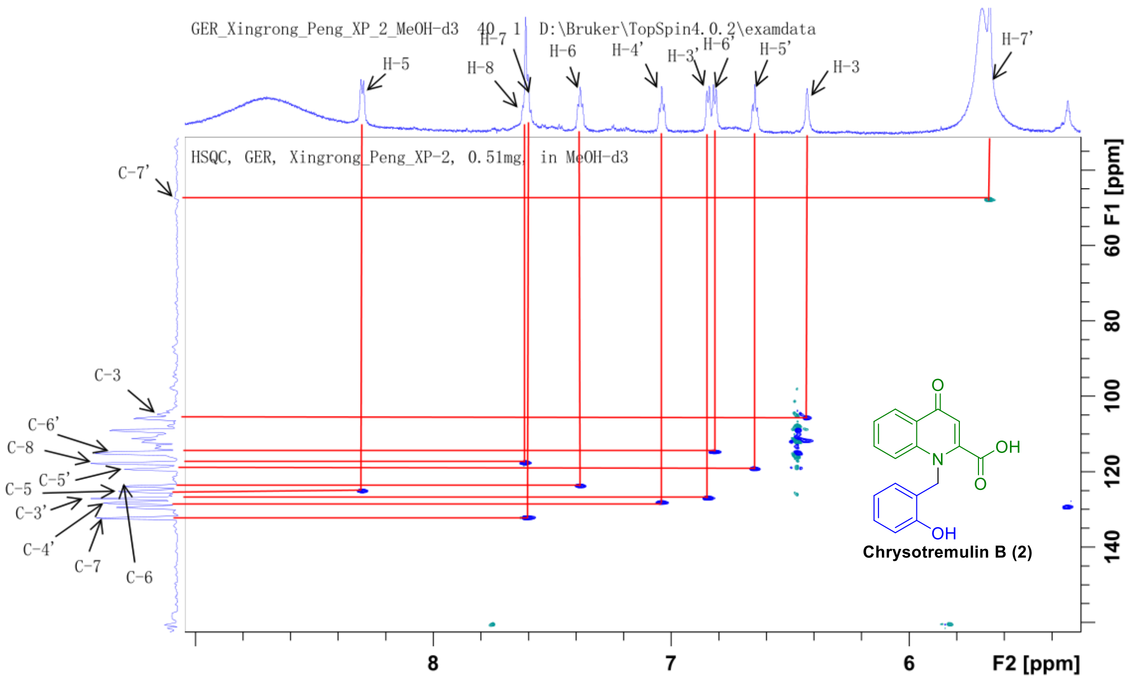

Figure S2.24 HSQC spectrum (700/175 MHz) of chrysotremulin B (**12**) from *C. tremulae* feces.

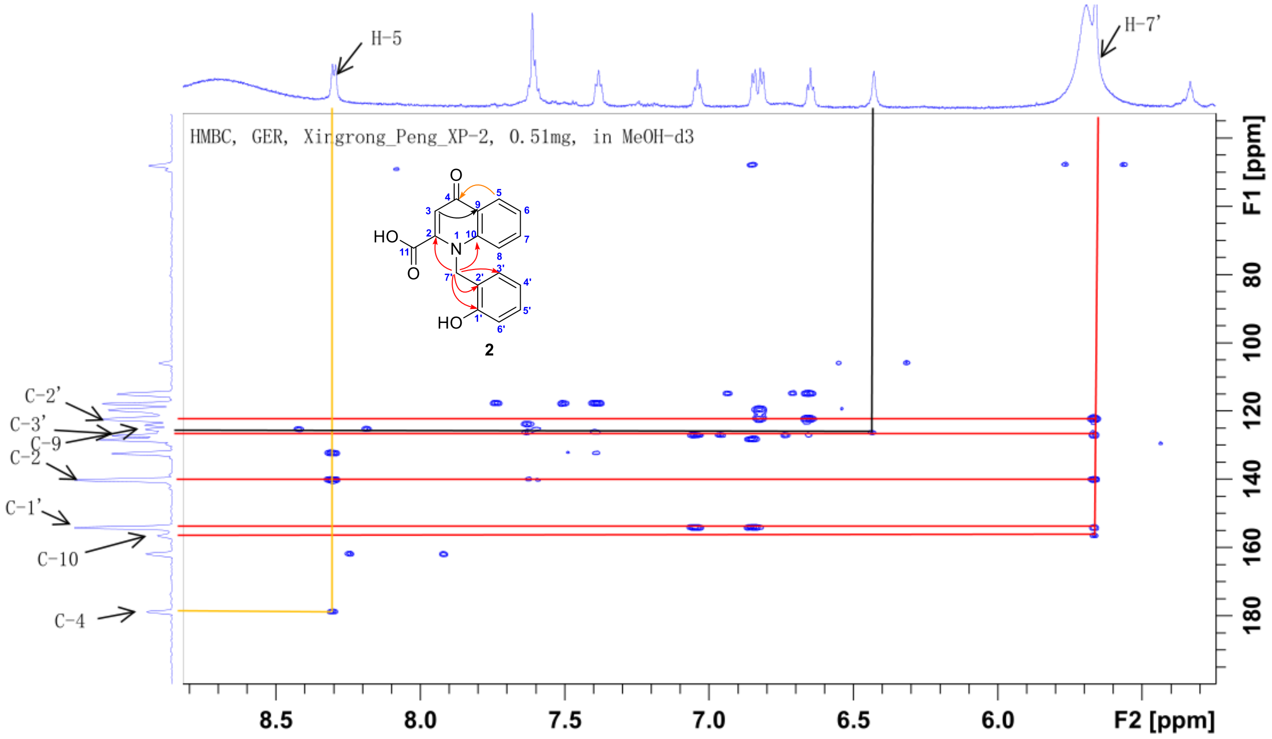

Figure S2.25 HMBC spectrum (700/175 MHz) of chrysotremulin B (**12**) from *C. tremulae* feces.

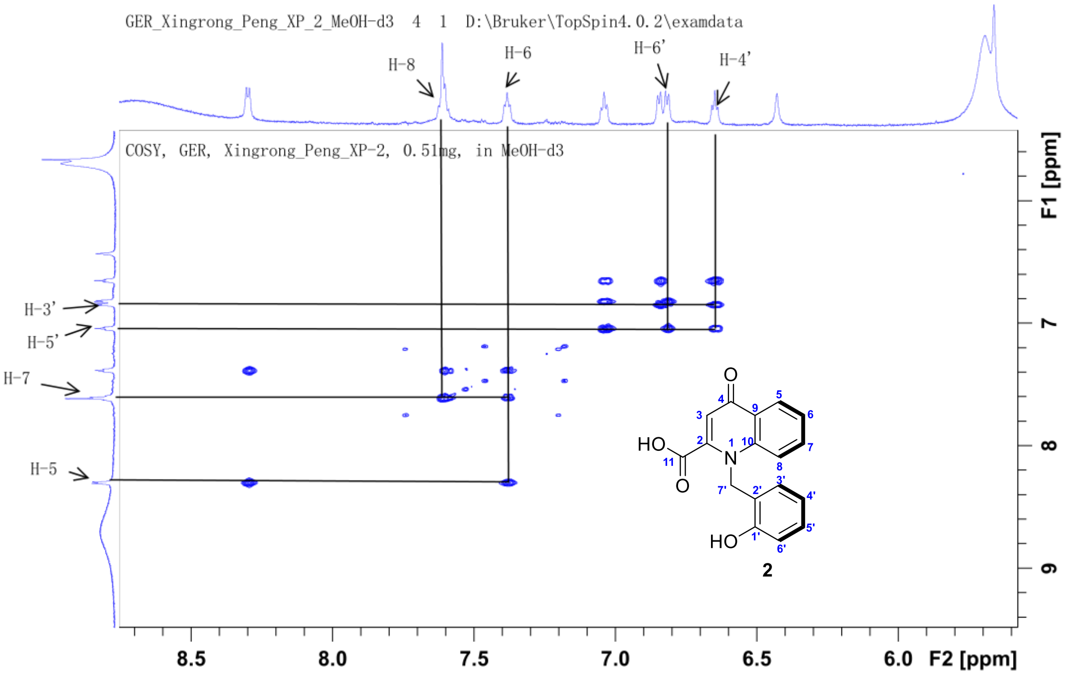

Figure S2.26 ^{1}H-^{1}H COSY spectrum (700 MHz) of chrysotremulin B (**12**) from *C. tremulae* feces.

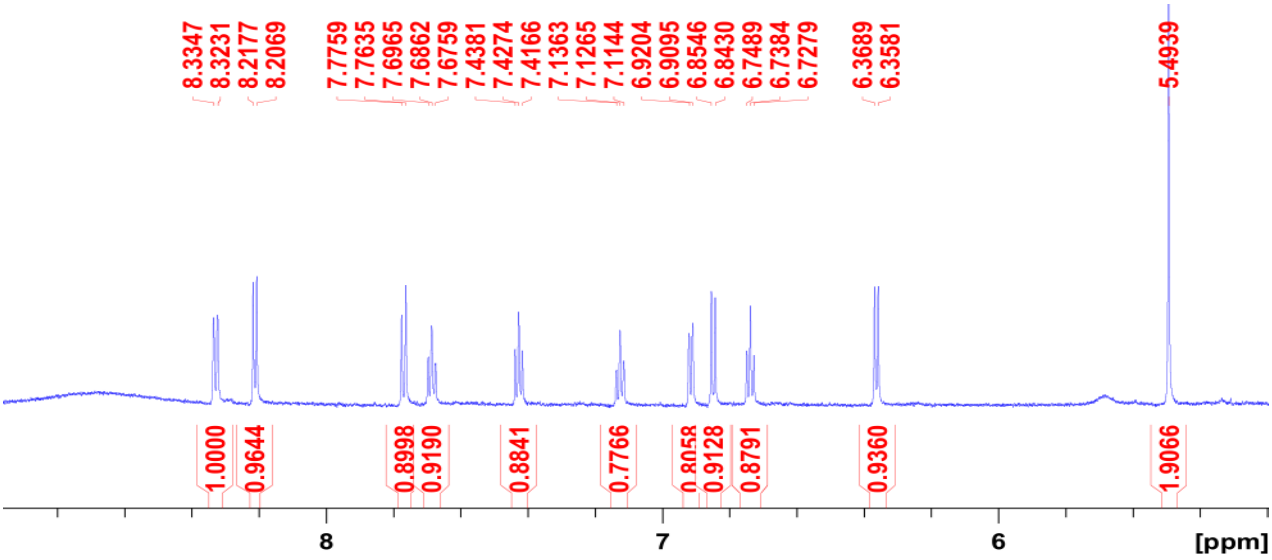

Figure S2.27 ^{1}H NMR spectrum (700 MHz) of chrysotremulin C (**13**) from *C. tremulae* feces.

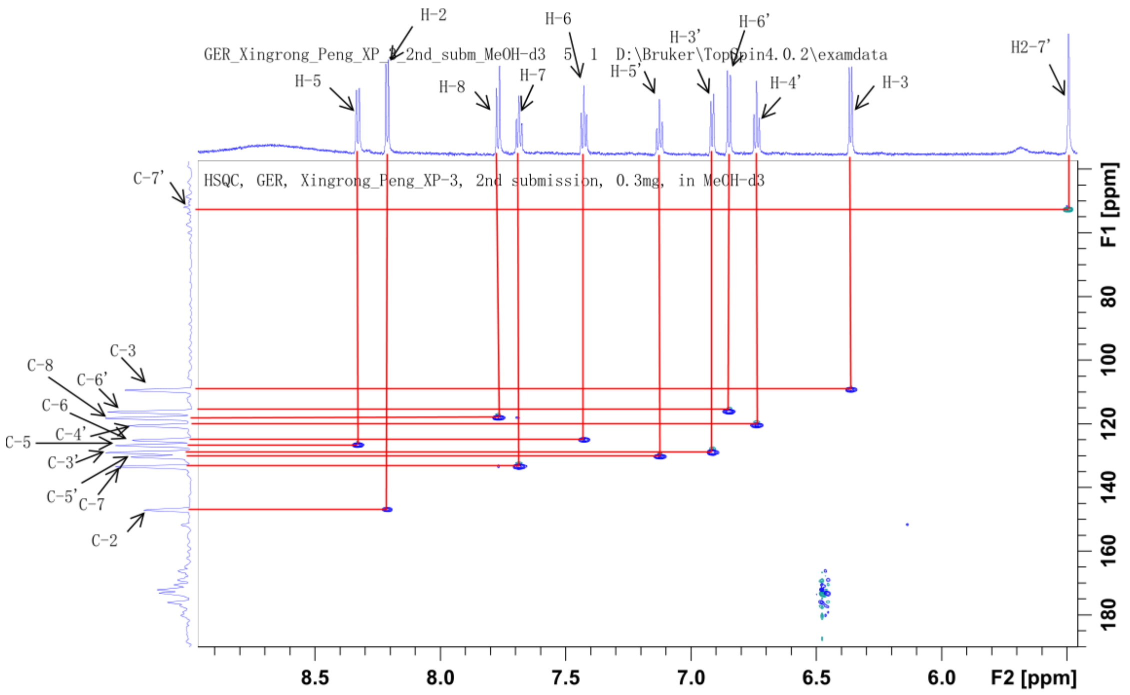

Figure S2.28 HSQC spectrum (700/175 MHz) of chrysotremulin C (**13**) from *C. tremulae* feces.

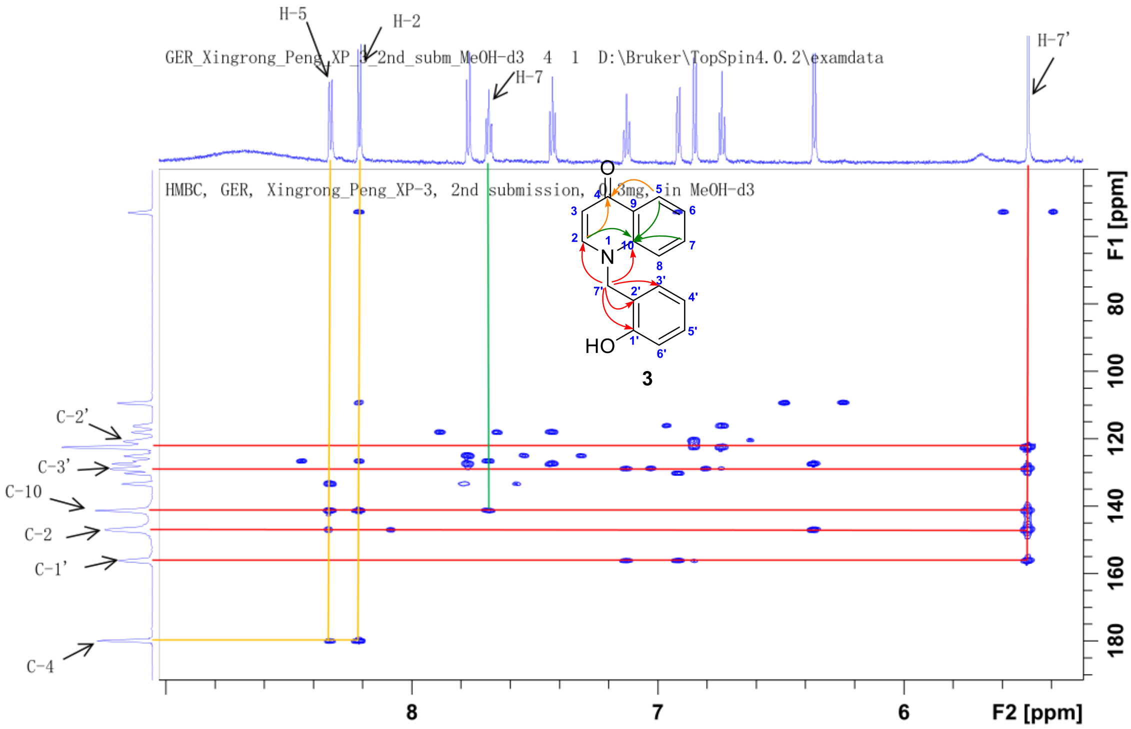

Figure S2.29 HMBC spectrum (700/175 MHz) of chrysotremulin C (**13**) from *C. tremulae* feces.

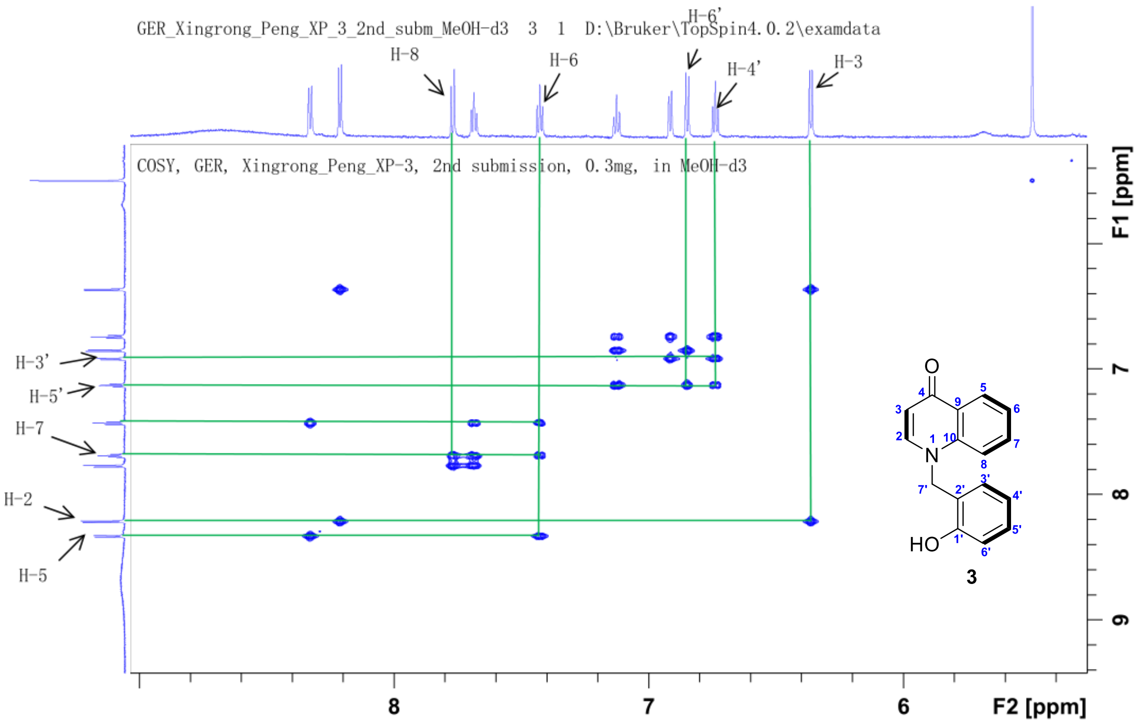

Figure S2.30 ^{1}H-^{1}H COSY spectrum (700/175 MHz) of chrysotremulin C (**13**) from *C. tremulae* feces.

NMR spectra of intermediates and syn-1 during chemical synthesis

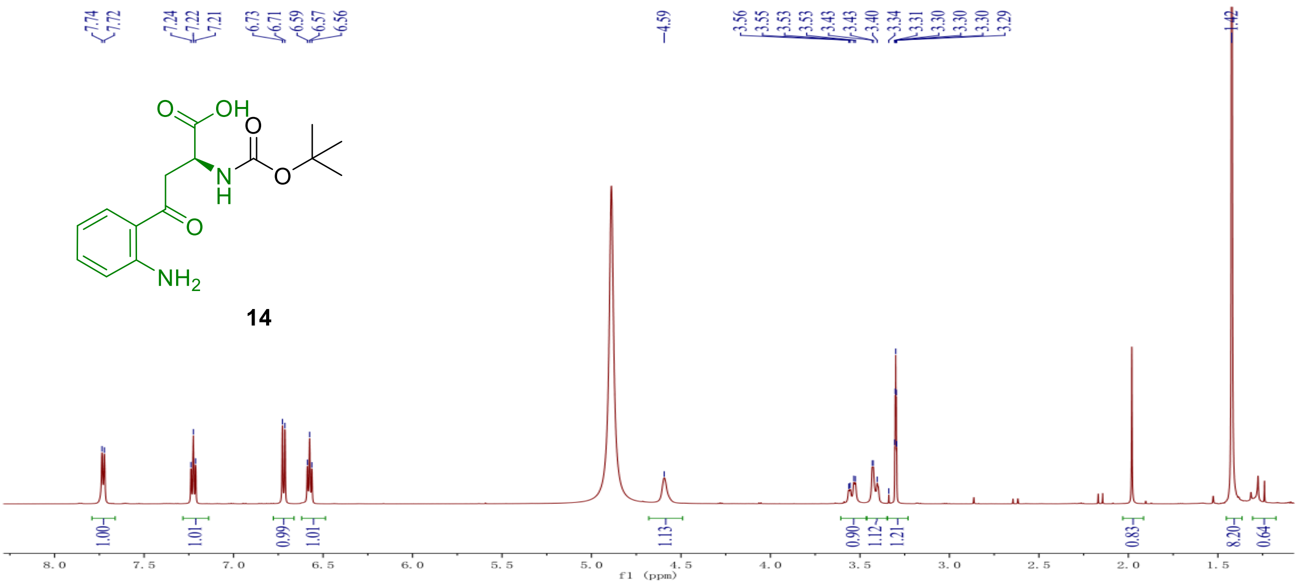

Figure S2.31 ^{1}H NMR spectrum (600 MH, CD_3OD) of compound **14** (chemical synthesis).

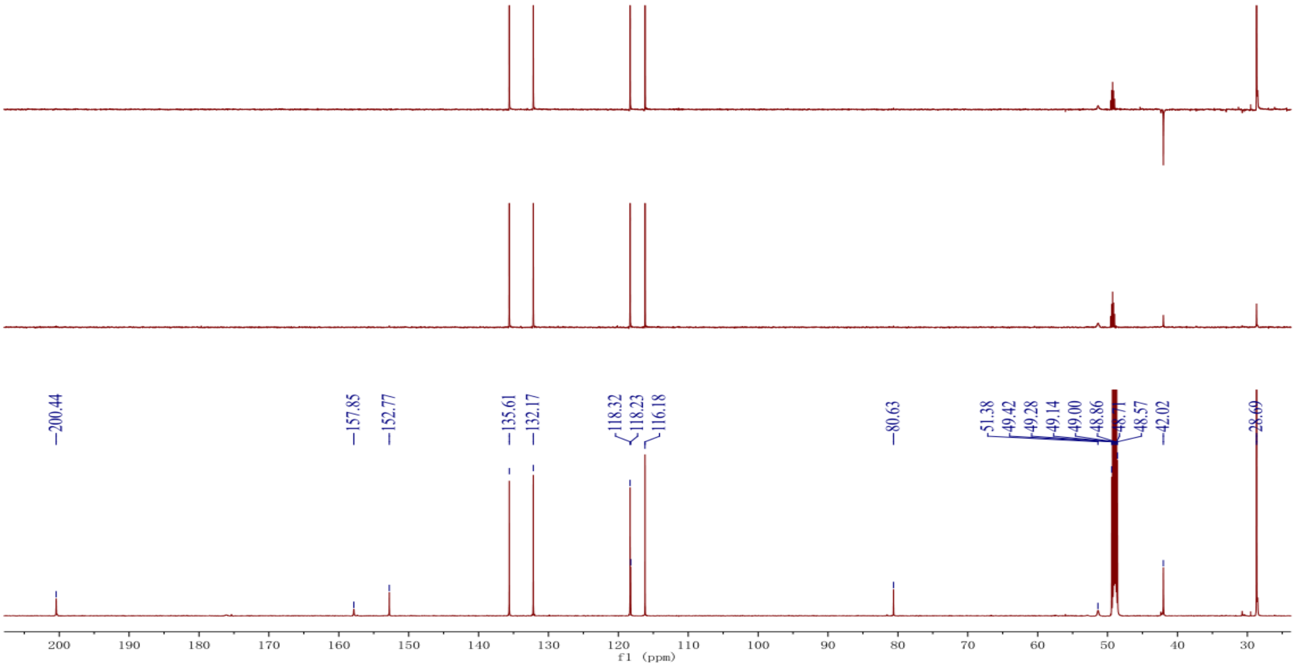

Figure S2.32 ^{13}C-DEPT NMR spectrum (150 MH, CD_3OD) of compound **14** (chemical synthesis).

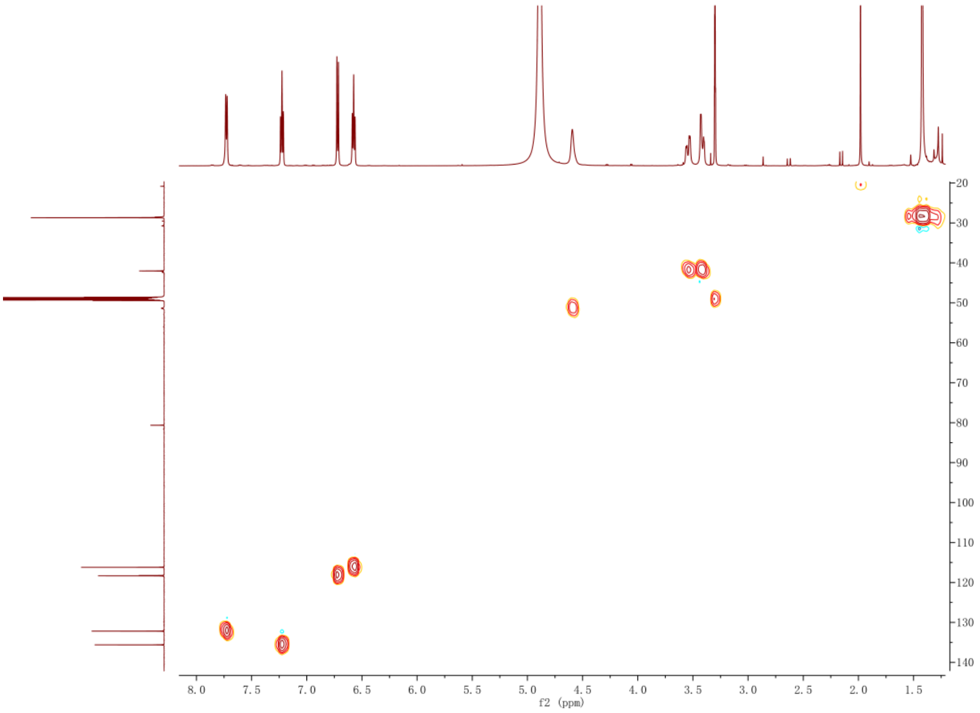

Figure S2.33 HSQC spectrum (600/150 MHz, CD_3OD) of compound **14** (chemical synthesis).

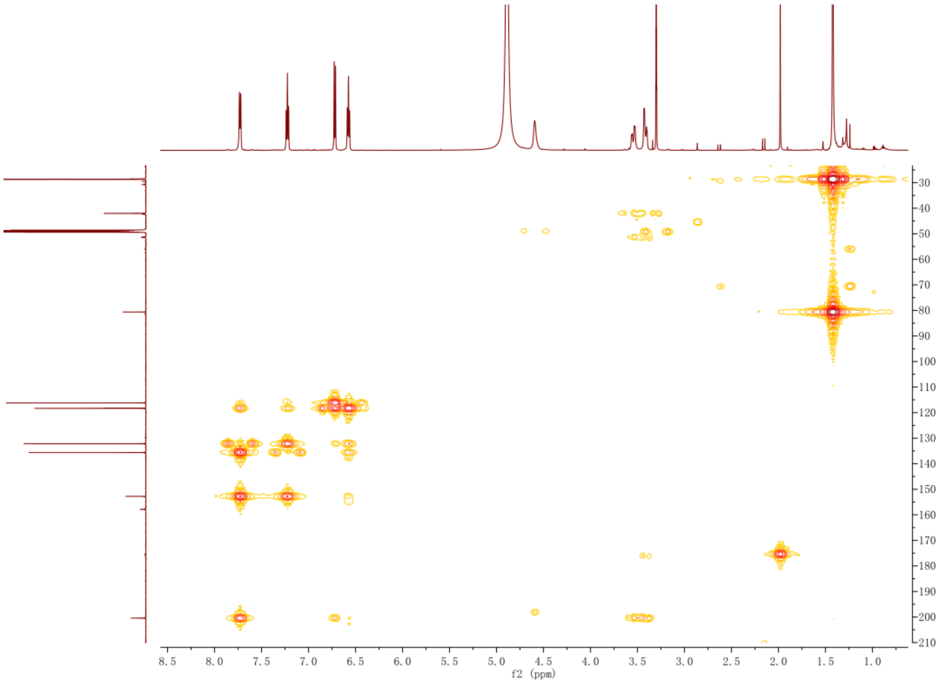

Figure S2.34 HMBC spectrum (600/150 MHz, CD_3OD) of compound **14** (chemical synthesis).

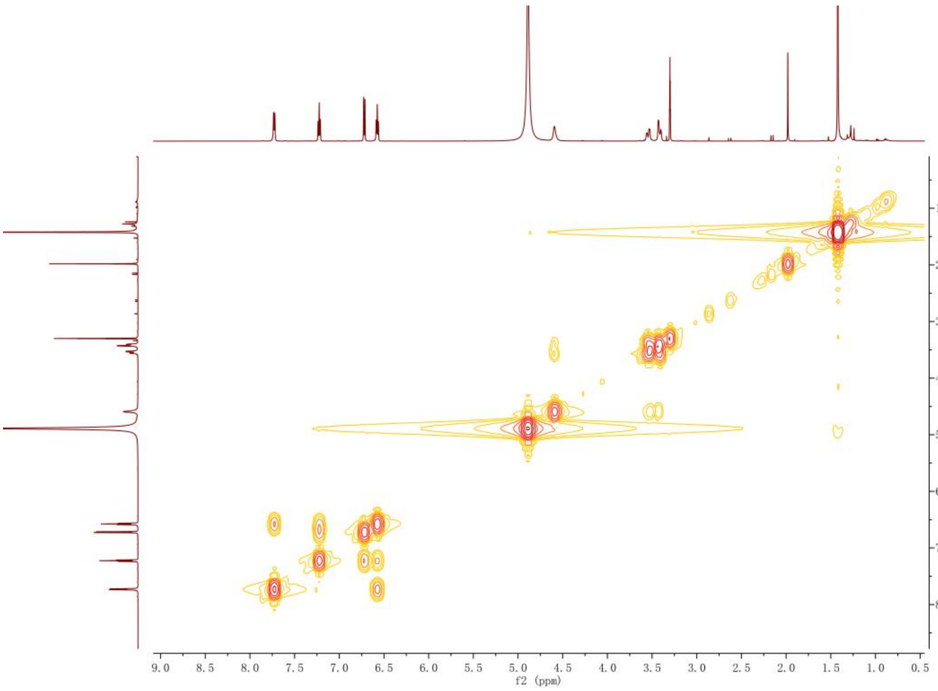

Figure S2.35 ^{1}H-^{1}H COSY spectrum (600 MHz, CD_3OD) of compound **14** (chemical synthesis).

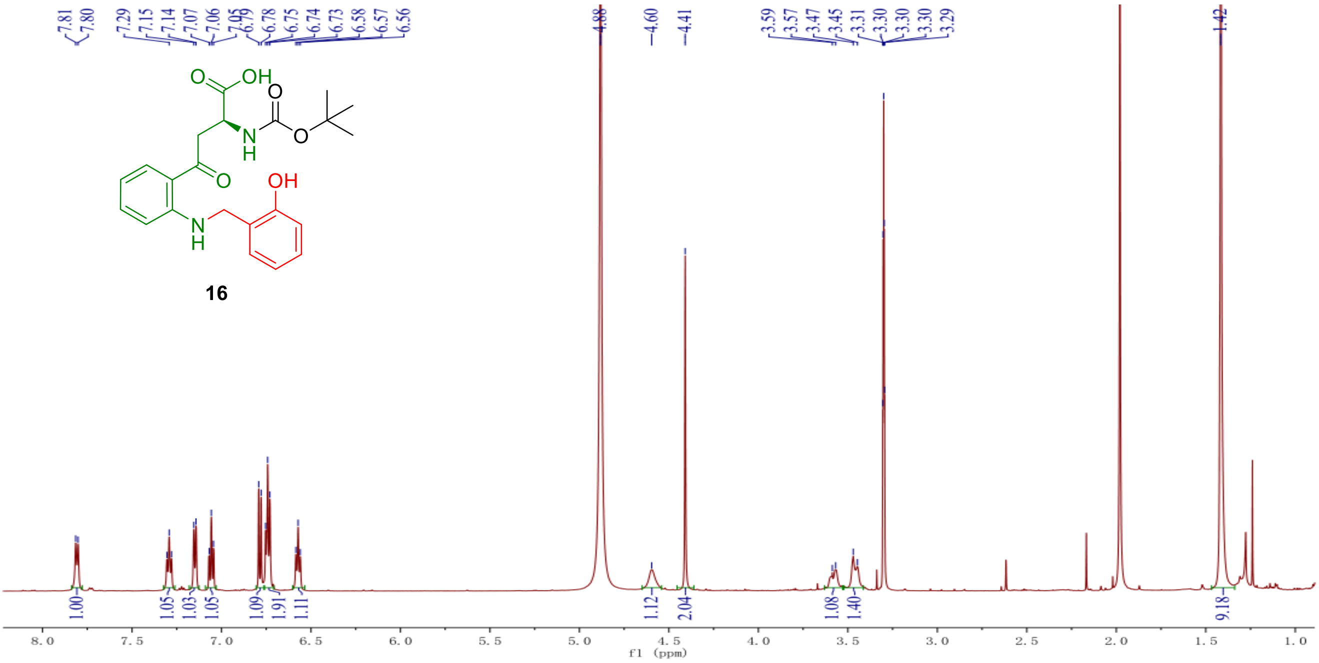

Figure S2.36 ^{1}H NMR spectrum (600 MH, CD_3OD) of compound **16** (chemical synthesis).

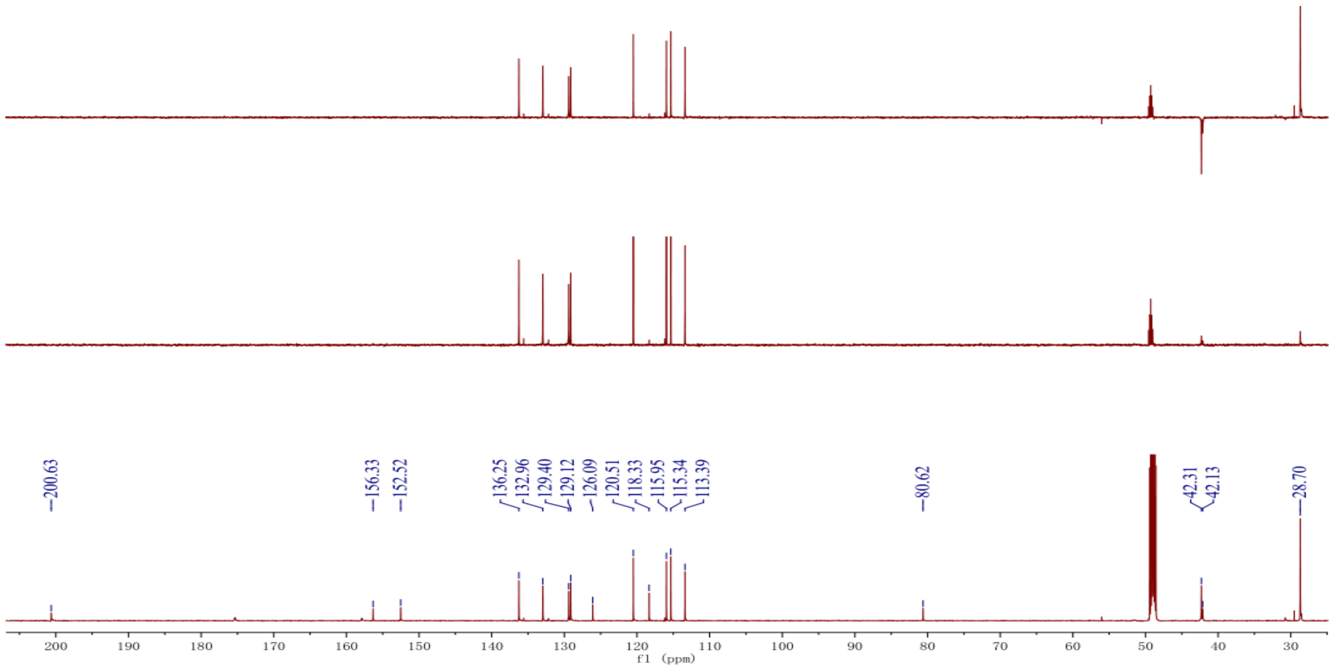

Figure S2.37 ^{13}C NMR spectrum (150 MH, CD_3OD) of compound **16** (chemical synthesis).

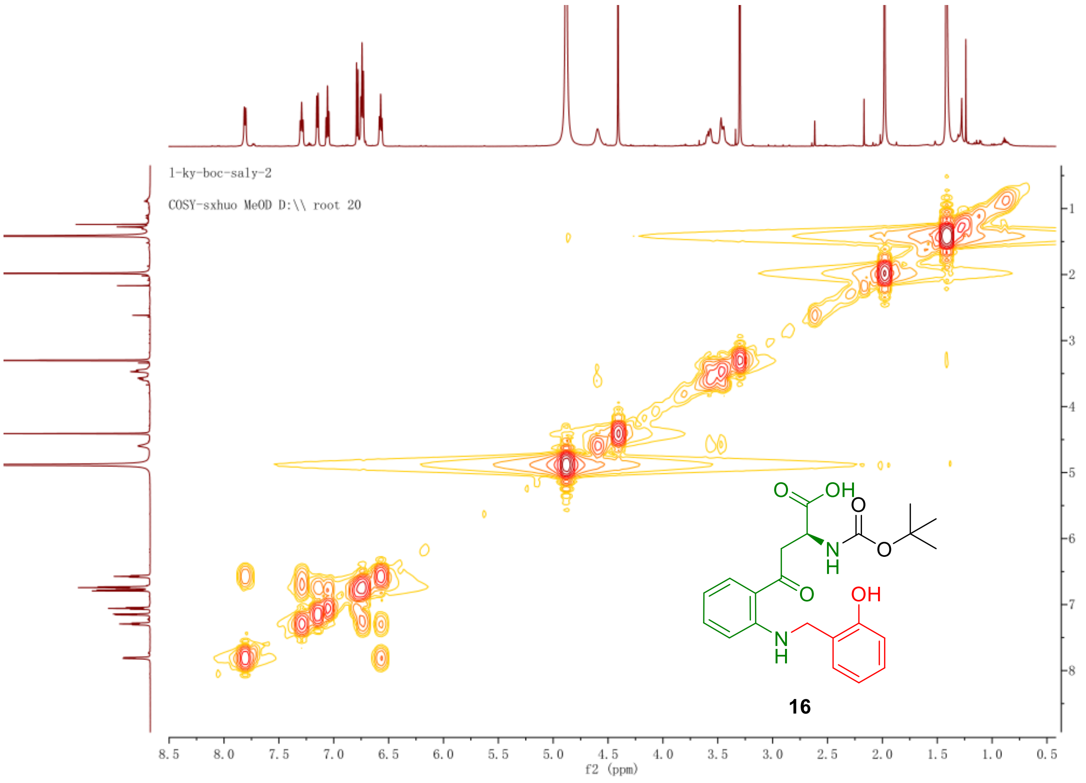

Figure S2.38 ^{1}H-^{1}H COSY spectrum (600 MH, CD_3OD) of compound **16** (chemical synthesis).

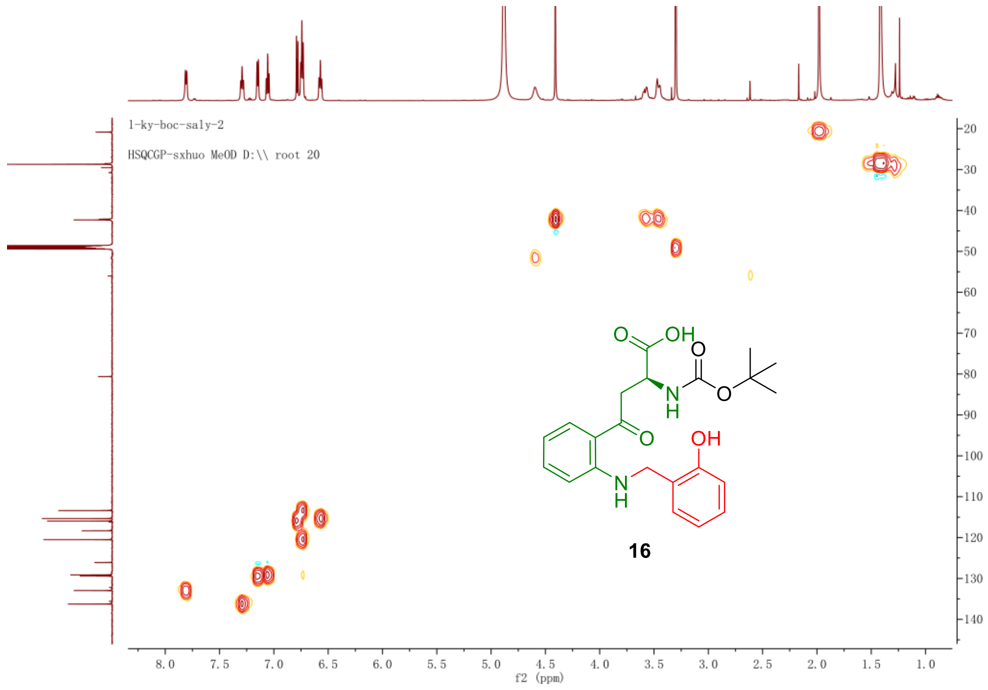

Figure S2.39 HSQC spectrum (600/150 MH, CD_3OD) of compound **16** (chemical synthesis).

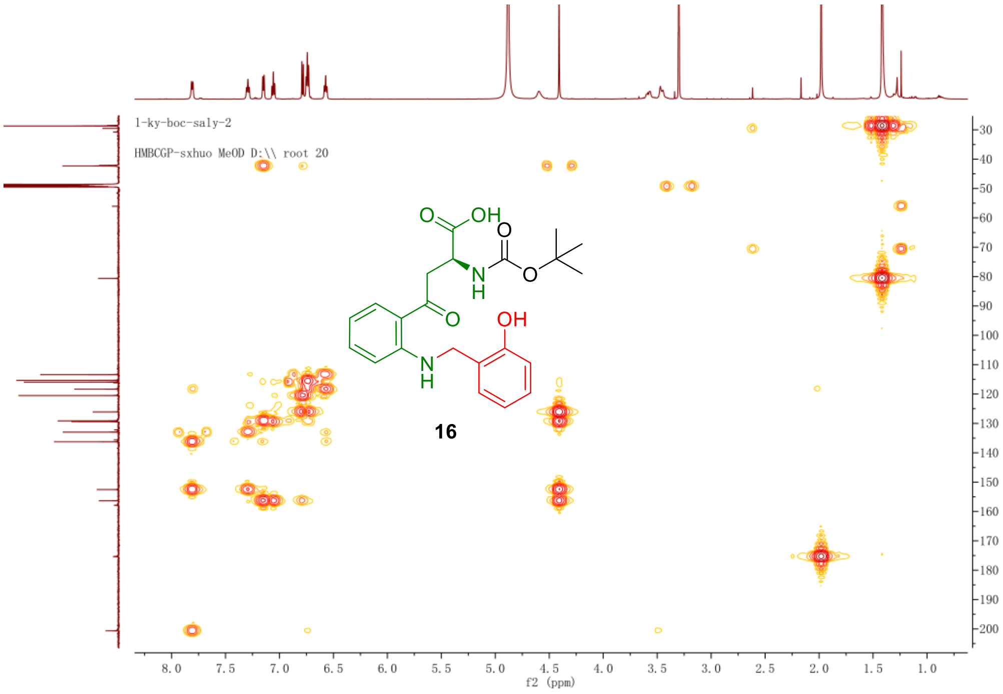

Figure S2.40 HMBC spectrum (600/150 MH, CD_3OD) of compound **16** (chemical synthesis).

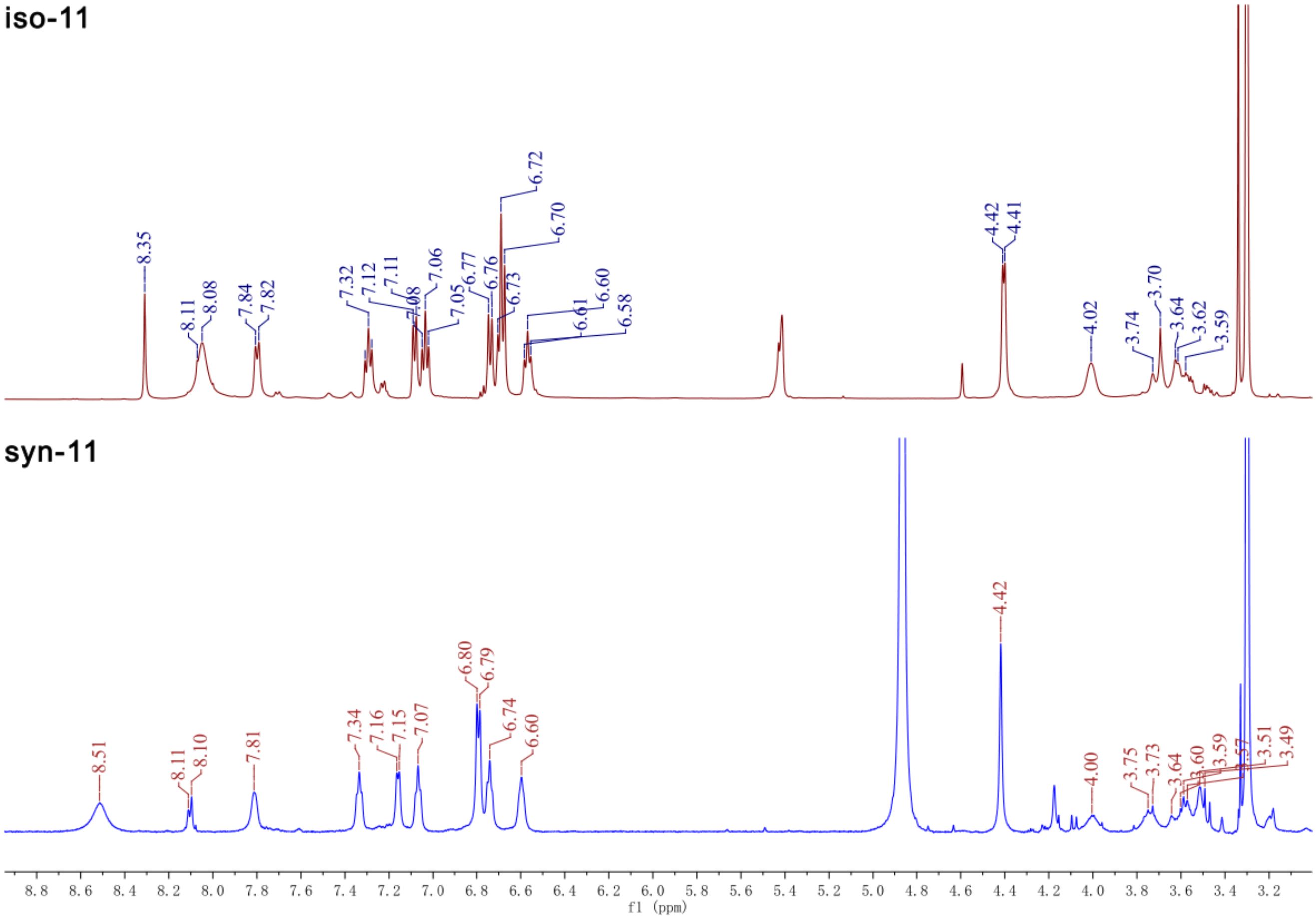

Figure S2.41 The comparison of ^{1}H NMR spectra between **iso-11** (700 MH, CD_3OH) and **syn-11** (600 MH, CD_3OD).

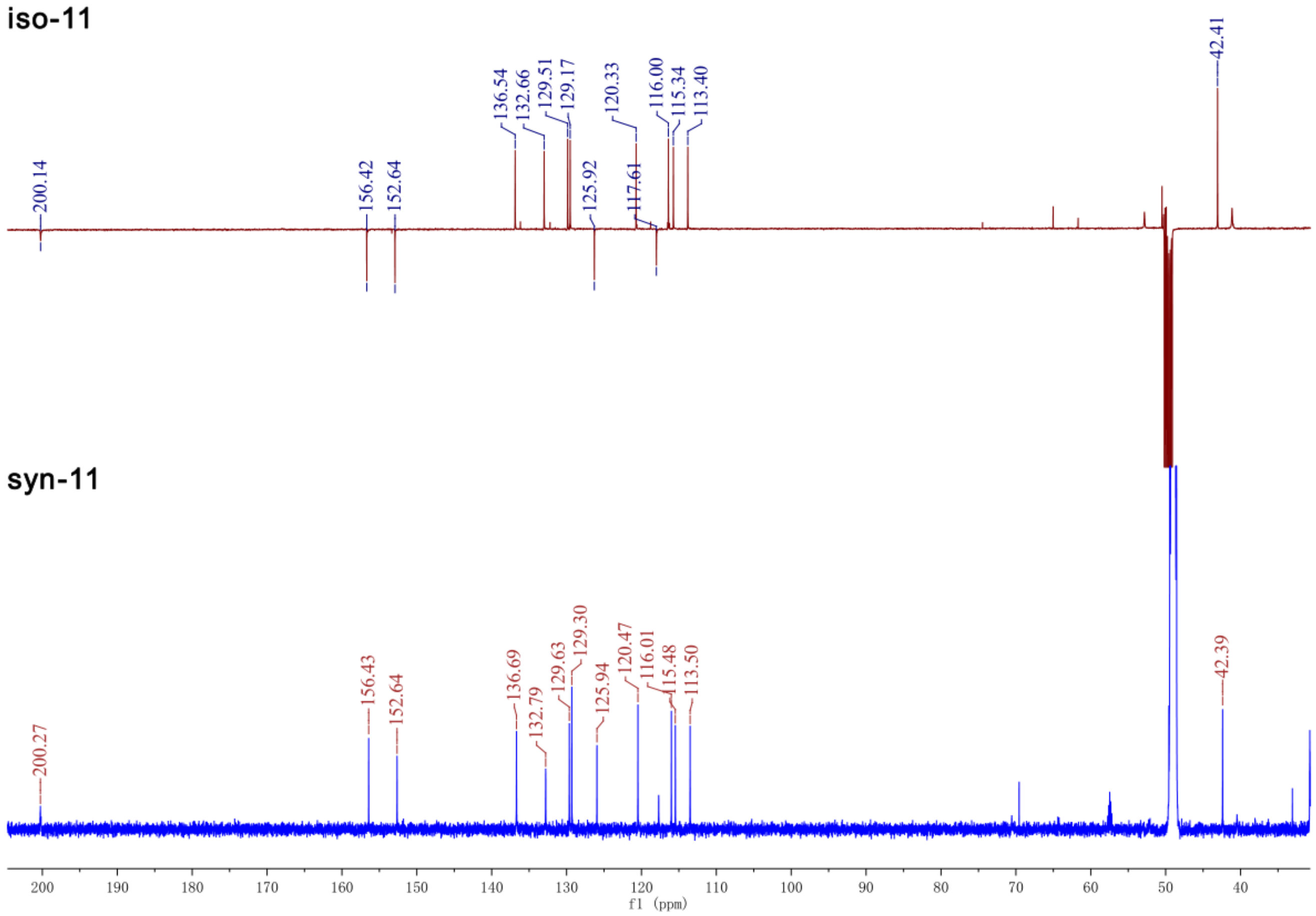

Figure S2.42 The comparison of ^{13}C NMR spectra between **iso-11** (175 MH, CD_3OH) and **syn-11** (150 MH, CD_3OD).

Qualitative Analysis Report

**Data Filename**	L-ky-saly.d	**Sample Name**	L-ky-saly
**Sample Type**	Sample	**Position**	P1-A6
**Instrument Name**	Instrument 1	**User Name**	
**Acq Method**	s.m	**Acquired Time**	5/25/2023 9:40:54 AM
**IRM Calibration Status**	Success	**DA Method**	PCDL.m
**Comment**			

Sample Group **Info.**
Acquisition SW 6200 series TOF/6500 series
Version Q-TOF B.05.01 (B5125.2)

User Spectra

Fragmentor Voltage	Collision Energy	Ionization Mode
135	0	ESI

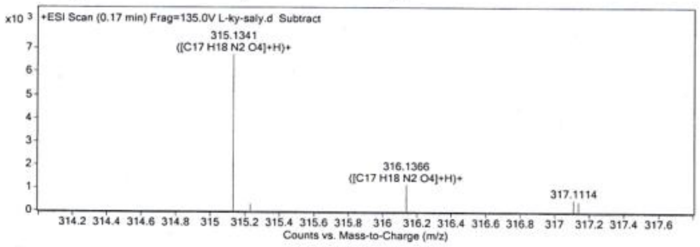

Peak List

m/z	z	Abund	Formula	Ion
84.9599	1	3331.88		
107.0504	1	1652.96		
125.9859	1	2543.85		
151.0353	1	1544.34		
158.0029	1	2227.12		
167.0128	1	1517.03		
209.0923	1	8213.99		
212.119	1	2839.76		
242.2843	1	1578.47		
315.1341	1	6744.89	C17 H18 N2 O4	(M+H)+

Formula Calculator Element Limits

Element	Min	Max
C	3	60
H	0	120
O	0	20
N	0	5

Formula Calculator Results

Formula	CalculatedMass	CalculatedMz	Mz	Diff. (mDa)	Diff. (ppm)	DBE
C17 H18 N2 O4	314.1267	315.1339	315.1341	-0.20	-0.63	10.0000

--- End Of Report ---

Agilent Technologies Page 1 of 1 Printed at: 4:13 PM on: 5/25/2023

Figure S2.43 HRESIMS spectrum of **syn-11**.

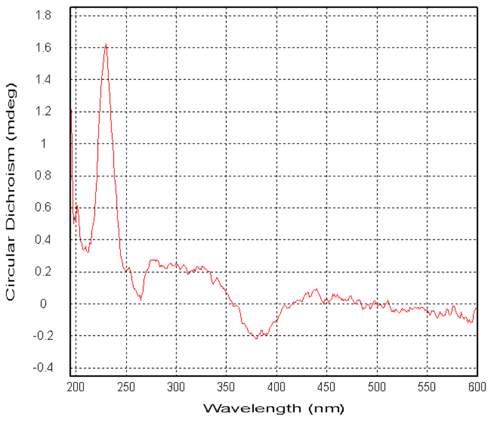

Figure S2.44 Experimental CD spectrum of **syn-11**.

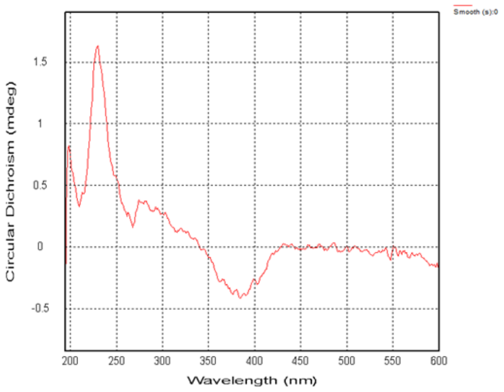

Figure S2.45 Experimental CD spectrum of chrysotremulae A (**11**) from *C. tramulae* feces**.**

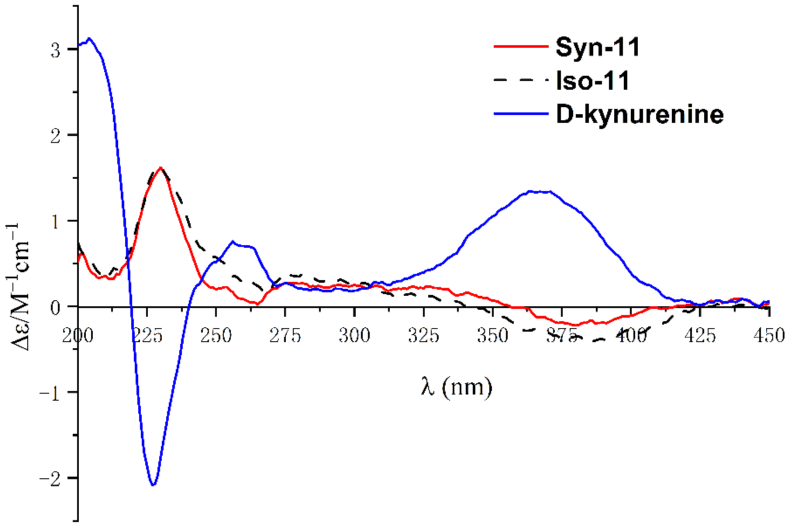

Figure S2.46 Comparison of experimental ECD spectra for chrysotremulae A (**11**) (Iso-**11**) from *C. tramulae* feces, synthetic compound **11** (Syn-**11**) and D-kynurenine**.**

Rudolph Research Analytical

This sample was measured on an Autopol VI, Serial #91058
Manufactured by Rudolph Research Analytical, Hackettstown, NJ, USA.

Measurement Date : Monday, 06-MAR-2023

Set Temperature : OFF

Time Delay : Disabled

Delay between Measurement : Disabled

n	Average	Std.Dev.	% RSD	Maximum	Minimum
5	-73.67	0.71	-0.96	-72.55	-74.51

S.No	Sample ID	Time	Result	Scale	OR °Arc	WLG.nm	Lg.mm	Conc.g/100ml	Temp.
1	XP-1	04:07:33 PM	-73.67	SR	-0.263	589	100.00	0.357	21.1
2	XP-1	04:07:39 PM	-73.67	SR	-0.263	589	100.00	0.357	21.1
3	XP-1	04:07:45 PM	-73.95	SR	-0.264	589	100.00	0.357	21.1
4	XP-1	04:07:52 PM	-74.51	SR	-0.266	589	100.00	0.357	21.1
5	XP-1	04:07:58 PM	-72.55	SR	-0.259	589	100.00	0.357	21.1

Figure S2.47 The optical rotation value of chrysotremulin A (**11**).

Rudolph Research Analytical

This sample was measured on an Autopol VI, Serial #91058
Manufactured by Rudolph Research Analytical, Hackettstown, NJ, USA.

Measurement Date : Thursday, 01-JUN-2023

Set Temperature : 25.0

Time Delay : Disabled

Delay between Measurement : Disabled

n	Average	Std.Dev.	% RSD	Maximum	Minimum
5	-11.27	0.50	-4.43	-10.91	-11.82

S.No	Sample ID	Time	Result	Scale	OR °Arc	WLG.nm	Lg.mm	Conc.g/100ml	Temp.
1	L-KY-SALY	01:09:33 PM	-11.82	SR	-0.013	589	100.00	0.110	25.0
2	L-KY-SALY	01:09:39 PM	-10.91	SR	-0.012	589	100.00	0.110	25.0
3	L-KY-SALY	01:09:45 PM	-11.82	SR	-0.013	589	100.00	0.110	25.0
4	L-KY-SALY	01:09:52 PM	-10.91	SR	-0.012	589	100.00	0.110	25.0
5	L-KY-SALY	01:09:58 PM	-10.91	SR	-0.012	589	100.00	0.110	25.0

Figure S2.48 The optical rotation value of synthetic compound **11**.

Table S2.2 Qualitative analysis of salicortin and its metabolites in other insect herbivores species that feed on leaves of *Populus nigra* trees. 10 mg feces were dissolved into 500 µL MeOH/H_2O (50%, v/v). 23 feces extracts (20 mg/mL) were analyzed by non-targeted LC-MS/MS.

Species	Family	Salicortin	Salicortinol	Salicin	Saligenin	Salicylic acid	1,2-dihydroxy-cyclohex-5-en-1-oic acid	Salicortin phosphate	Salicin phosphate	Quinic acid ester-4.16	Quinic acid ester-5.10
Saperda carcharias	Cerambycidae	x	√	√	√	√	√	x	x	x	x
Chrysomela populi	Chrysomelidae	√	√	√	√	√	√	x	√	√	x
Chrysomela saliceti	Chrysomelidae	√	√	√	√	x	√	x	x	√	√
Chrysomela tremulae	Chrysomelidae	√	√	√	√	√	√	√	√	√	√
Clytra quatripunctata	Chrysomelidae	√	√	√	x	x	√	x	x	√	√
Plagiodera versicolora	Chrysomelidae	√	√	√	√	√	√	x	√	√	√
Amata mogadorensis	Erebidae	√	x	√	√	√	√	x	x	√	√
Lymantria dispar	Erebidae	√	√	√	√	√	√	x	√	√	√
Orgya antiqua	Erebidae	√	√	√	√	√	√	x	√	√	√
Gastropacha populifolia	Lasiocampidae	√	√	√	√	√	√	x	√	√	√
Acronicta alni	Noctuidae	√	√	√	√	√	√	x	√	√	√
Acronicta leporina	Noctuidae	√	√	√	√	√	√	x	x	√	x
Catocala nupta	Noctuidae	√	√	√	√	√	√	x	√	√	√
Furcula bifida	Notodintodae	√	√	√	√	√	√	x	x	√	√
Cerura vinula	Notodontidae	√	√	√	√	√	√	x	√	√	√
Clostera curtula	Notodontidae	√	√	√	√	√	√	x	√	√	√
Notodonta	Notodontidae	√	√	√	√	√	√	x	x	√	√

ziczac											
Pterostoma palpinum	Notodontidae	x	x	√	√	√	√	x	x	x	√
Laothoe populi	Sphingidae	√	√	√	√	√	√	x	√	√	√
Pachysphinx occidentalis	Sphingidae	√	√	√	√	√	√	x	√	√	x
Smerinthus ocellata	Sphingidae	√	√	√	√	√	√	x	√	√	x
Smerinthus planus	Sphingidae	√	√	√	√	√	√	x	√	√	√
Cladius grandis	Tenthredinidae	√	√	√	√	√	√	x	x	√	x
Stauronematus platycerus	Tenthredinidae	x	x	√	√	√	√	x	x	√	x

Note: X means no detected; √ means detected

Table S2.3 Quantitative analysis of tryptophan metabolites and compound **11** in other insect herbivore species that feed on *Populus nigra* trees. 10 mg feces were dissolved into 500 μL MeOH/H_2O (50%, v/v) and diluted to 2 mg/mL. 23 feces extracts (2 mg/mL) were analyzed by targeted LC-MS/MS. – not detected.

Species	Family	Kynurenine (mg/g)	Kynurenic acid (mg/g)	4-Hydroxyquinoline (mg/g)	Compound 11 (mg/g)
Saperda carcharias	Cerambycidae	1.150	1.732	0.034	0.014
Chrysomela populi	Chrysomelidae	0.653	0.069	0.089	0.842
Chrysomela saliceti	Chrysomelidae	1.455	0.157	0.081	2.079
Chrysomela tremulae	Chrysomelidae	0.719	0.100	0.065	0.713
Clytra quatripunctata	Chrysomelidae	0.003	8.270	–	–
Plagiodera versicolora	Chrysomelidae	1.257	0.136	0.120	4.362
Amata mogadorensis	Erebidae	–	–	–	–
Lymantria dispar	Erebidae	–	0.011	–	–
Orgya antiqua	Erebidae	–	0.021	–	–
Gastropacha populifolia	Lasiocampidae	–	0.014	–	–
Acronicta alni	Noctuidae	–	0.041	0.008	–
Acronicta leporina	Noctuidae	–	–	–	–
Catocala nupta	Noctuidae	–	–	–	–
Furcula bifida	Notodintodae	0.017	0.014	0.018	–
Cerura vinula	Notodontidae	–	0.015	–	–
Clostera curtula	Notodontidae	–	0.005	–	–
Notodonta ziczac	Notodontidae	–	0.007	–	–
Pterostoma palpinum	Notodontidae	–	–	0.007	–
Laothoe populi	Sphingidae	0.039	0.296	0.011	0.007
Pachysphinx occidentalis	Sphingidae	0.050	0.007	–	–
Smerinthus ocellata	Sphingidae	0.008	0.038	0.074	0.035
Smerinthus planus	Sphingidae	–	0.061	–	–

Cladius grandis	Tenthredinidae	–	0.167	–	–
Stauronematus platycerus	Tenthredinidae	–	0.005	–	–

SI-3: Effects of saligenin on the larval development of *Chrysomela tremulae*

Materials and Methods

To investigate the effect of saligenin for the specialist poplar leaf beetle, *C. tremulae*, we designed a saligenin coating experiment. In this bioassay experiment, four groups including control, low concentration, medium concentration, and high concentration groups were set. The natural concentration of saligenin in poplar leaves was calculated as 6.45 mg/g (fresh weight) based on the assumption of salicin, salicortin, homaloside D, and salicortin-6-benzoate converting into saligenin using published concentrations of those salicinoids in *Populus nigra* leaves (1). One leaf was about 1.25 g and the natural content of saligenin was 8.06 mg for one leaf. The low concentration, medium concentration, and high concentration were prepared as 3.02, 6.06, and 20.2 mg/mL (saligenin/water, m/v; Merck, Germany) and then adding 400 µL of this solution (saligenin in water), respectively, thus mimicking 0.15, 0.3, and 1.0-fold natural content of saligenin equivalents.

120 *C. tremulae* larvae hatched (instar 1) from eggs and reared on *P. nigra* leaves in the laboratory were randomly divided into four groups. Each group has three Petri-dish replicates (with 10 larvae in each Petri-dish). 12 poplar leaves were cut from three young trees reared in the greenhouse under the above conditions. After cutting, leaf petioles were immediately inserted into 5 mL tubes filled with tap water. Three leaves (average weight: 1.25 g per leaf) were always assigned to each of four treatment groups that were either coated with 400 µL water, or low, medium, and high concentration of saligenin in water solution, respectively, using a 200 µL pipette. After the evaporation of solvent, each leaf was placed into a Petridish (90 mm) together with ten larvae. Before that, the total weight of then larvae were recorded. Then, every 2 or 3 days, the weight of the larval groups in each of the Petri dishes and the leaf area loss due to larval feeding was recorded. With each leaf change, the old leaves of each treatment group were collected and flash-frozen in liquid nitrogen. Then, these leaves were stored in -80 ºC. Once leaf beetles produced enough feces, we also collected them and kept them at –20 ºC in the freezer. We regularly took photographs of the larvae in the different treatment groups until they pupated. During the 24 and 48 hours it takes for a pupa to become an adult, we recorded their weight. Finally, all collected feces and leaves were chemically analyzed by UPLC-QTOF-MS and targeted UPLC-MS/MS (details see section 3 in SI-1).

Supplementary figures

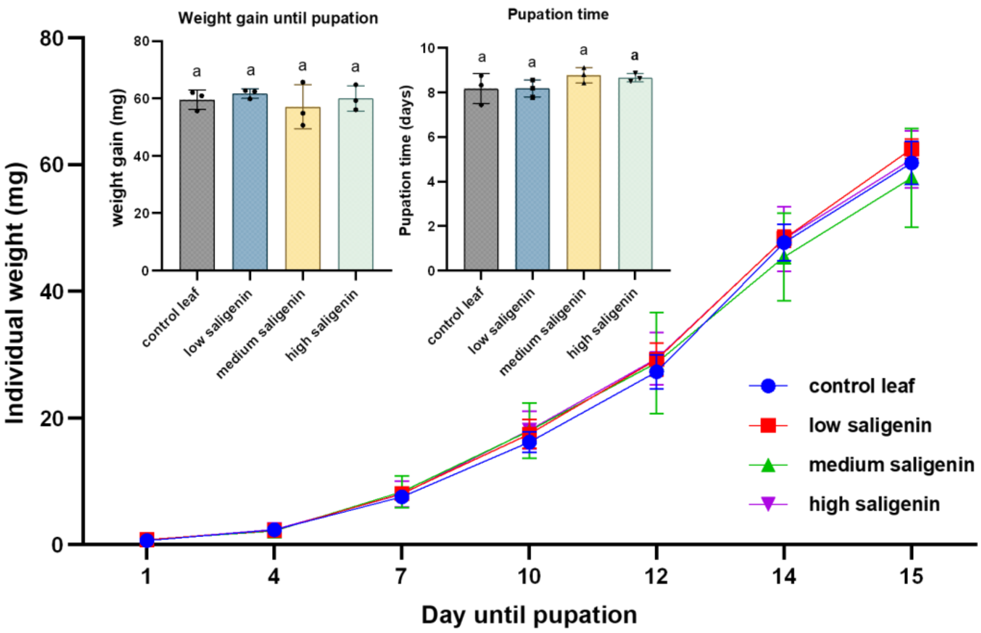

Figure S3.1 The weight and weight gain until pupation of *Chrysomela tremulae* larvae, and pupation time in four groups: control (natural concentration), low concentration (adding 3.03 mg saligenin /mL water solution), medium concentration (adding 6.06 mg saligenin /mL water solution), and high concentration (adding 20.2 mg saligenin /mL water solution). One-way analysis of variance (ANOVA) with $P < 0.05$ being considered statistical significance. Bars represent mean ± SEM, the saligenin coating experiment repeated biologically three times.

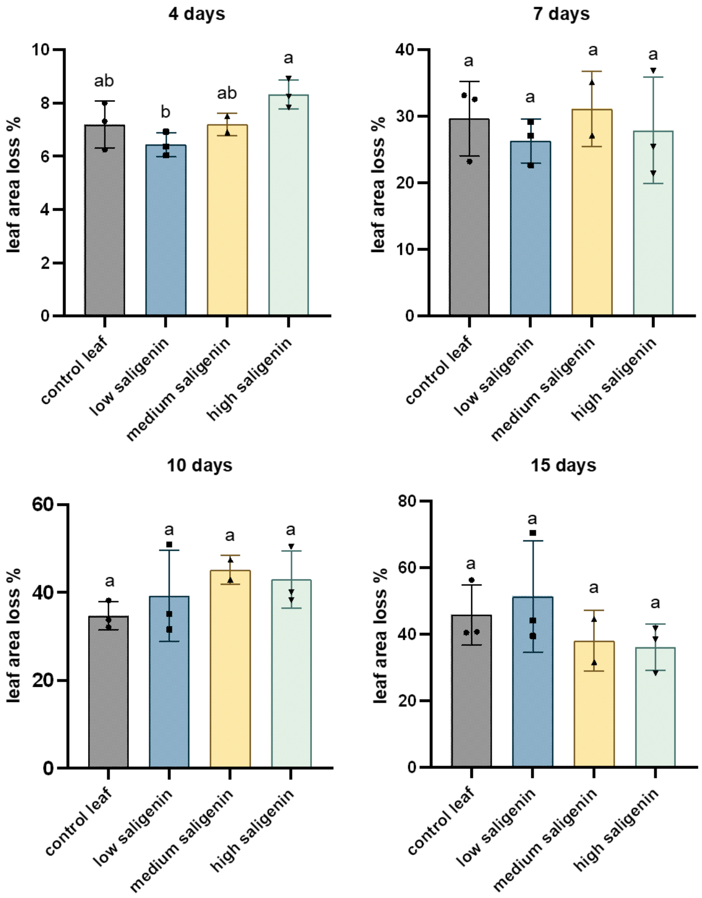

Figure S3.2 *Chrysomela tremulae* larvae inflicted leaf area loss in *Populus nigra* leaves from larval hatching to pupation in the saligenin coating experiment. Data was acquired as full scan peak intensities of extracted ion traces on an LC-Q-TOF-MS. Four groups: control (natural concentration), low concentration (adding 3.03 mg saligenin /mL water solution), medium concentration (adding 6.06 mg saligenin /mL water solution), and high concentration (adding 20.2 mg saligenin /mL water solution). Each group has three Petri-dish replicates (with 10 larvae in each Petri-dish). Results shown represent the means ± standard error (SE). Different letters indicate significant differences [$P < 0.05$, one-way analysis of variance (ANOVA) with Tukey's post hoc test] (n = 30).

SI-4: Microbial community characterization of *Chrysomela tremulae*

Materials and Methods

1. Characterization of the native bacterial communities of *C. tremulae*

(1) Sample preparation

To characterize the microbiota of larvae and adult beetles, insects were collected from the rearing and anesthetized for 1 min at −20°C, washed individually in 0.1% SDS sterile solution inside of a microcentrifuge tube, and shaken for 30 s. They were then transferred to another microcentrifuge tube with 70% ethanol and again shaken for 30 s. Subsequently, each specimen was washed thrice in sterile 1x PBS and dissected as described elsewhere (10). Five guts were pooled in 1.5 mL microcentrifuge tubes and flash frozen in liquid nitrogen to create each biological replicate. Six replicates were prepared for each life stage. Additionally, five whole adults were pooled to create a whole adult reference sample. Likewise, five whole larvae were pooled to create another whole larva reference sample.

(2) DNA extraction

The insect samples were homogenized in 1.5 mL microcentrifuge tubes with liquid nitrogen and sterile micro pestles. The MasterPureTM complete DNA and RNA isolation Kit (Epicenter Technologies) was used following the manufacturer's protocol, with an additional incubation step with 4 μL lysozyme (100 mg/mL) at 37°C before protein digestion and a 600 μL phenol/chloroform/isoamyl alcohol (Carl-Roth A156) step before DNA precipitation. A positive control (ZymoBIOMICSTM Microbial Community Standard, Zymo Research, USA) and negative controls (extraction reagents without sample tissue) were included throughout the extraction steps. The DNA was re-suspended in ultrapure Millipore water and the concentration was measured on a Qubit fluorometer using a 1x dsDNA High sensitivity assay (Thermo Fisher Scientific).

(3) Amplicon sequencing

Altogether, 12 beetle gut samples (6 per life stage), 2 whole individuals, one mock community as positive control and 1 negative extraction control were sequenced. Bacterial 16S rRNA gene regions were sequenced by a commercial provider (StarSeq, Mainz, Germany) on a MiSeq platform (Illumina) using double indexing and a paired end approach with a read length of 300 nucleotides. primers 341F (5′-CCTACGGGNGGCWGCAG-3′) and 806R (5′ GACTACNVGGGTWTCTAATCC-3′) were used to sequence the V3-V4 regions of the bacterial 16S rRNA (11).

(4) Sequence analysis

The reads were demultiplexed onboard in MiSeq Reporter software, allowing for one mismatch. The demultiplexed reads were processed following the *DADA2* pipeline (version 1.28.0) (12). The last 30 base pairs of all forward reads and the last 50 base pairs of the reverse reads were trimmed to remove nucleotides with low quality scores. The maximum expected error was set to 2 and the minimal overlap for merging the sequences was set to 12 nucleotides. Chimera removal was done with the "consensus" method. The resulting bacterial sequences were further filtered to remove any consensus sequence of length below 300 bp, as shorter reads were likely resulting from sequencing artifacts. The database used for taxonomy assignment was SILVA trainset v138.1 (13). The identities of the most abundant taxa were verified by blasting the sequence against the NCBI Nucleotide database National Center for Biotechnology Information (1988). Nucleotide. Available online at: https://www.ncbi.nlm.nih.gov/nucleotide/(accessed December 5, 2023).). The "phyloseq" R package (version 1.44.0) (14) was used to analyses the amplicon sequence variant (ASV) tables. Single reads and taxa that could not be classified as "bacteria" (e.g., chloroplasts) were removed from the dataset. The data were not rarefied to improve the detection of differentially abundant species (15). The alpha diversity was estimated with the Shannon and Simpson indices, and the beta diversity among groups was visualized with principal component analysis (PCoA) of the BrayCurtis dissimilarity index. All analyses were carried out in R version 4.3.0 (R Core Team (2023). R: A Language and Environment for Statistical Computing. Available online at: https://www.R-project.org (accessed February 5, 2024)).

(5) Quantification of the bacterial titers in larvae and adults

The DNA obtained for the microbial community analysis was also used to estimate bacterial titers in the gut and in whole individuals. Quantitative PCR (qPCR) was carried out in 20 µL reactions using Blue S'Green qPCR mix (Biozym), 1 µL template DNA and 0.4 µM of each primer. The bacterial 16S primers EUB338mod (5′-TCCTACGGGAGGCAGCAG-3′) and EUB518 (5′-ATTACCGCGGCTGCTGG-3′) were used (16). Standard curves with defined copy numbers of the 16S rRNA gene were created by amplifying the fragment first, followed by purification and determination of the DNA concentration via NanoDrop1000 (Peqlab, Germany). After determination of the DNA concentration, eight serials 1:10 dilutions were prepared to generate the standards. One µL of each dilution was included in the qPCR reaction to standardize the measurements across reactions. The number of copies of DNA in the standard dilutions was calculated using the formula $N = \frac{DNA\ amount\ (ng) \times 6.022 \times 10^{23}\ (\frac{\text{number}}{\text{mole}})}{ragment\ length\ (bp) \times 1\times10^{9}(\frac{\text{ng}}{\text{g}}) \times 330 \frac{\text{g}}{\text{mole}}}$. The DNA copy numbers in the samples were estimated taking into account the efficiency of each reaction, using the formula: $X_0 = 10^{(Cq-b)\cdot m-1}$ where X is the estimated DNA

copy numbers, Cq is the quantification cycle of the qPCR, b is the intercept of the reaction's standard curve and m is the slope.

2. Saligenin feeding assay with microbiota-depleted *C. tremulae* larvae

(1) Egg surface sterilization protocol to obtain microbiota-depleted *C. tremulae* larvae

To obtain microbiota-depleted larvae, groups of approximately 50 eggs were collected using a moist, sterile paintbrush and placed in a 1.5 mL microcentrifuge tube. The eggs were rinsed with 1 mL of sterile 1x PBS by gently pipetting up and down for 30 seconds. 100 µL of this suspension were plated on a sterile LB agar and the rest of the PBS was discarded. Then, the eggs were rinsed in the same fashion using 70% ethanol for 30 seconds. The ethanol was carefully removed and discarded. The PBS washing step was carried out again thrice and 100 µL of the last wash were plated on sterile LB agar. The eggs were transferred to a Petri dish with 4% water agar using a new, sterile brush. They were incubated at 25°C and 70% RH for 5 days. The plated PBS washes were incubated for 5 days at 28°C and the success of the treatment was assessed by comparing the number of bacterial colonies of the first versus the last wash corresponding to the surface-sterilized eggs (Figure S4.4).

(2) Saligenin feeding bioassay with microbiota-depleted, reinfected and control *C. tremulae* larvae

To assess the role of the gut microbiota of *C. tremulae* in the saligenin metabolism, we randomly assigned 300 eggs from different egg clutches to the following microbial treatments: microbiota-depleted (surface-sterilized eggs), control (unaltered native microbiota on the egg surface), and reinfected (surface-sterilized eggs which were re-inoculated with the PBS suspension of the first washing step). After treating them, the eggs were incubated on sterile 4% water agar plates at 25°C and 70% RH for 5 days. Newly hatched larvae were assigned at random to water-coated or high saligenin-coated poplar leaves placed individually in 150 mm diameter Petri dishes (see Supporting Information 3 for bioassay methodology). Each treatment combination (microbial manipulation and leaf coating) had three repetitions consisting of 12 larvae per leaf. Larvae were transferred to freshly coated leaves every 1-2 days. The feces were collected from each replicate for chemical analyses on days 10 and 11 post- hatching. On day 12 post-hatching, defensive secretions were collected by gently probing the larvae with a 10 µL pipette tip and then were pooled to create a single sample per replicate. On the same day, all the larvae were weighed individually in a precision scale (Mettler-Toledo, Germany) and transferred to freshly coated leaves for pupation. The time to pupation and time to adult emergence were recorded for each individual. The differences in survival per treatment were assessed with

the Kaplan-Meier estimate. The statistical analyses were carried out in R version 4.3.0

(3) Characterization of the bacterial communities of larvae belonging to different treatments

One larva per repetition was removed from the assay on day 12 after hatching to quantify their salicinoid metabolite contents and characterize their bacterial communities. Each larva was individually homogenized with a sterile micro pestle in a 1.5 mL micro centrifuge tube. Then it was extracted with 1 mL of methanol/water (50:50, v:v) containing the standards described in the salicinoid metabolite quantification step (see section 3). After removing the liquid phase for chemical analysis, the samples were left under the extraction hood for 20 minutes to remove any methanol excess. DNA extraction, amplicon sequencing and sequence analysis were carried out on the remaining tissue pellet following the same methods used to characterize the native bacterial communities (see section 1.).

3. Quantification of kynurenine, kynurenic acid, 4-hydroxyquinoline, chrysotremulin A (11), chrysotremulin C (13), tryptophane, phenylalanine, tyrosine, methionine, salicylic acid, salicylic acid glucoside, and saligenin by LC-MS/MS.

For quantitative analysis of metabolites in feces and whole larvae, 3 to 55 mg of feces and 20 to 35 mg of whole larvae were extracted with 1 mL of methanol/water (50:50, v:v) containing the internal standards D4-SA (40 ng/mL; Santa Cruz Biotechnology, USA), a mixture of $^{15}N/^{13}C$ labeled amino acids (10 µg/mL; Isotec, Miamisburg, OH, USA; U-^{13}C-^{15}N-Phe 560 ng/mL, U-^{13}C-^{15}N-Tyr 477 ng/mL, U-^{13}C-^{15}N-Met 217 ng/mL) and D5-tryptophane (1045 ng/mL; Cambridge Isotope Laboratories, Inc.; Andover, MA).

Analysis was performed by LC-MS/MS on an Agilent 1260 series HPLC system (Agilent Technologies) coupled to a tandem mass spectrometer QTRAP 6500 (SCIEX, Darmstadt, Germany) (details in Supporting information-1). Quantification of all metabolites was carried out using internal standards applying experimentally determined response factors, except for salicylalcohol which was quantified based on an external standard curve generated with an authentic standard (Sigma-Aldrich, Taufkirchen, Germany).

Table S4.1 Details of analysis of metabolites by LC-MS/MS [HPLC 1260 (Agilent Technologies)-QTRAP6500 (SCIEX)] in positive ionization mode

Q1	Q3	RT (min)	Compound	Internal std	RF	DP	EP	CE	CXP
315	209	5.6	Chrysotremulin A	D4-SA	0.37	20	10	13	10
252	146	6.1	Chrysotremulin C	D5-Trp	0.88	20	4.5	21	6
209	192	1.5	kynurenine	D4-SA	0.19	20	10	13	10
190	144	3.2	kynurenic acid	D4-SA	3.40	150	10	25	10
146	77	3.1	4-OH-quinoline	D4-SA	0.22	20	10	37	10
150.2	104.1	0.6	Met	U-13C,15N-Met	1.00	20	4	13	10
166.2	120.2	1.4	Phe	U-13C,15N-Phe	1.00	20	6	17	10
182.1	136.2	0.8	Tyr	U-13C,15N-Tyr	1.00	20	7	17	10
205.2	188.1	2.9	Trp	D5-Trp	1.00	20	4.5	13	10
156.2	109.1	0.6	U-13C,15N-Met			20	4	13	4
176.2	129.2	1.4	U-13C,15N-Phe			20	6	17	4
192.1	145.2	0.8	U-13C,15N-Tyr			20	7	17	4
210	193	2.9	D5-Trp			20	4.5	13	6
143	69	5.8	D4-SA			30	8	37	12

Table S4.2 Details of analysis of metabolites by LC-MS/MS [HPLC 1260 (Agilent Technologies)-QTRAP6500 (SCIEX)] in negative ionization mode

Q1	Q3	RT (min)	Compound	Internal std	RF	DP	EP	CE	CXP
123	93	3.5	salicylalcohol	ext. Standard	-	-20	-10	-22	-2
136.93	93	5.9	salicylic acid	D4-SA	1.00	-20	-8	-22	-2
299.128	136.9	3.3	salicylic acid glucoside	D4-SA	8.27	-20	-10	-18	-21
140.934	97	5.7	D4-SA			-20	-8	-22	0

Table S4.3 Source of standards used for the determination of response factors for the analysis of metabolites by LC-MS/MS [HPLC 1260 (Agilent Technologies)-QTRAP6500 (SCIEX)]

Analyte	Name	Company
kynurenine	L-Kynurenine Hydrate	Sigma-Aldrich, Taufkirchen, Germany
kynurenic acid	kynurenic acid hydrate	TCI Chemicals, Eschborn, Germany
4-OH-quinoline	4-Quinolinol	Sigma-Aldrich, Taufkirchen, Germany
Chrysotremulin A (**11**)		isolated in the course of this study
Chrysotremulin C (**13**)		isolated in the course of this study

Supplementary figures

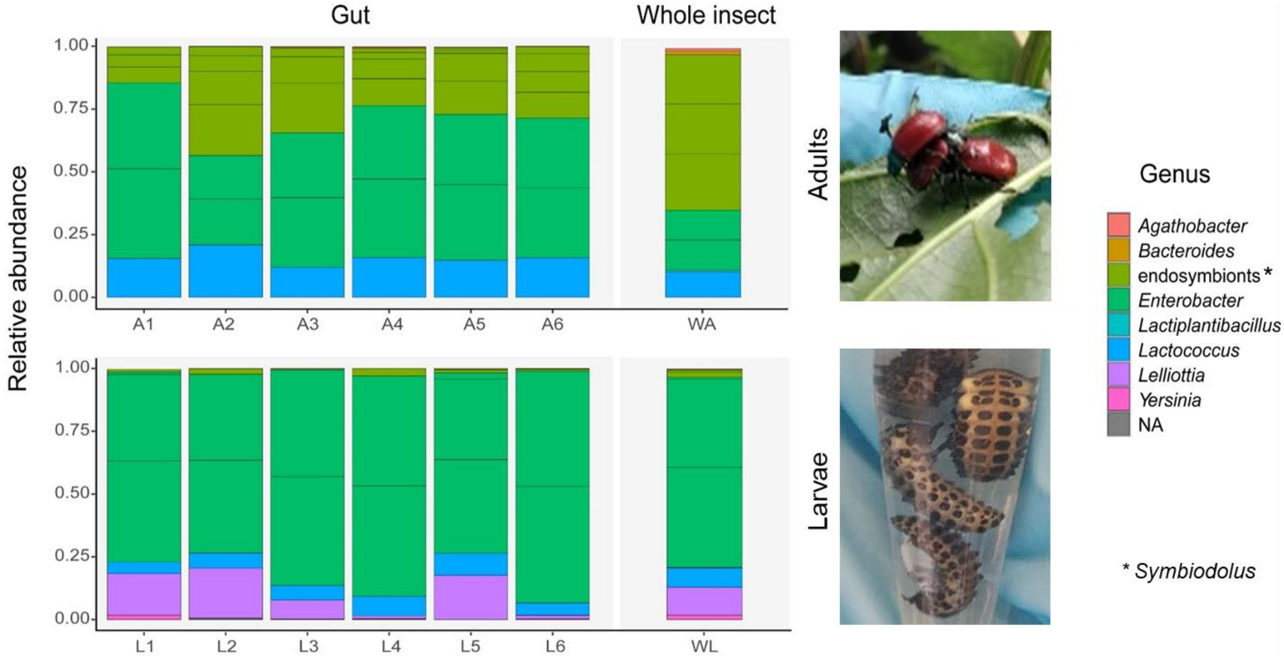

Figure S4.1 Top 15 amplicon sequencing variants (ASVs) from dissected guts and whole individuals of adults and third-instar larvae of *Chrysomela tremulae* reared in captivity. Relative abundances are indicated at ASV level; taxa marked with an asterisk (*) were refined to species by nucleotide blast against the NCBI database. When blasted against NCBI database, the assigned taxonomy corresponded to *Symbiodolus clandestinus*.

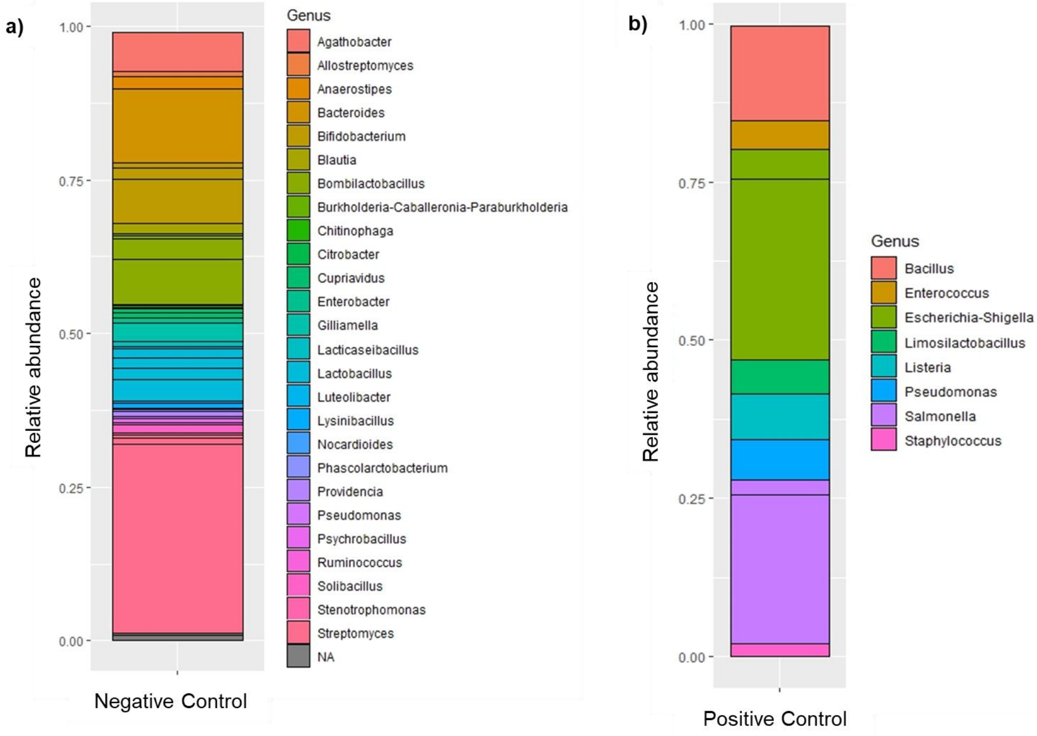

Figure S4.2 Top 15 amplicon sequencing variants (ASVs) from a) negative control (reagents used throughout

the extraction) and b) positive control (ZymoBIOMICS Microbial Community Standard).

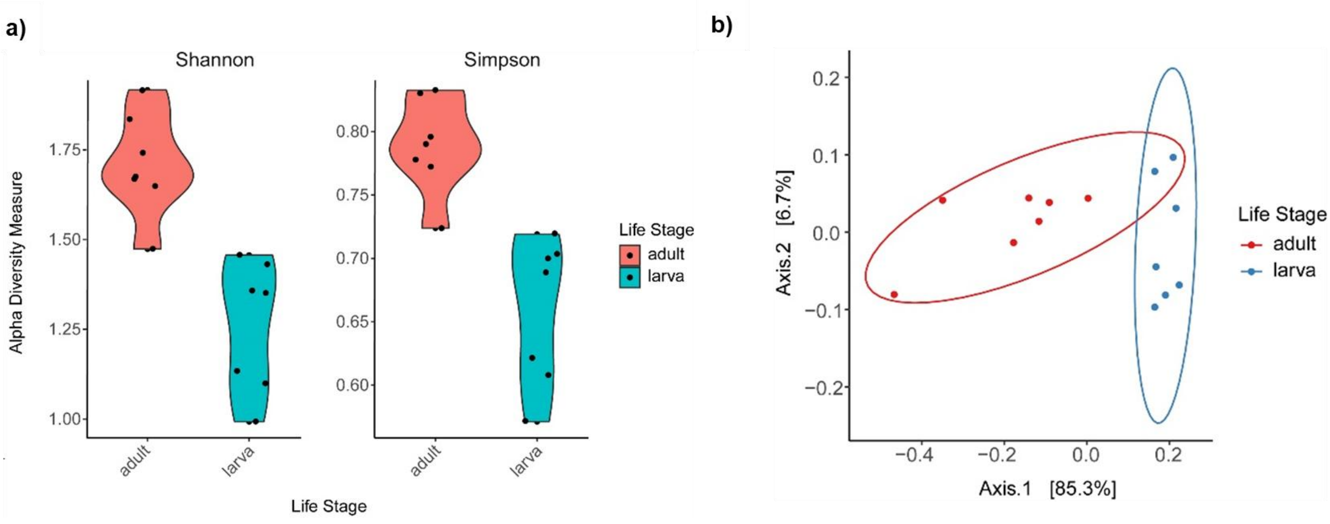

Figure S4.3 Diversity of the bacterial communities in larvae and adult *Chrysomela tremulae*. a) α-diversity measured by Shannon and Simpson indices, b) Principal Coordinate Analysis (PCoA) of Bray Curtis dissimilarity matrix depicting the *β*-diversity.

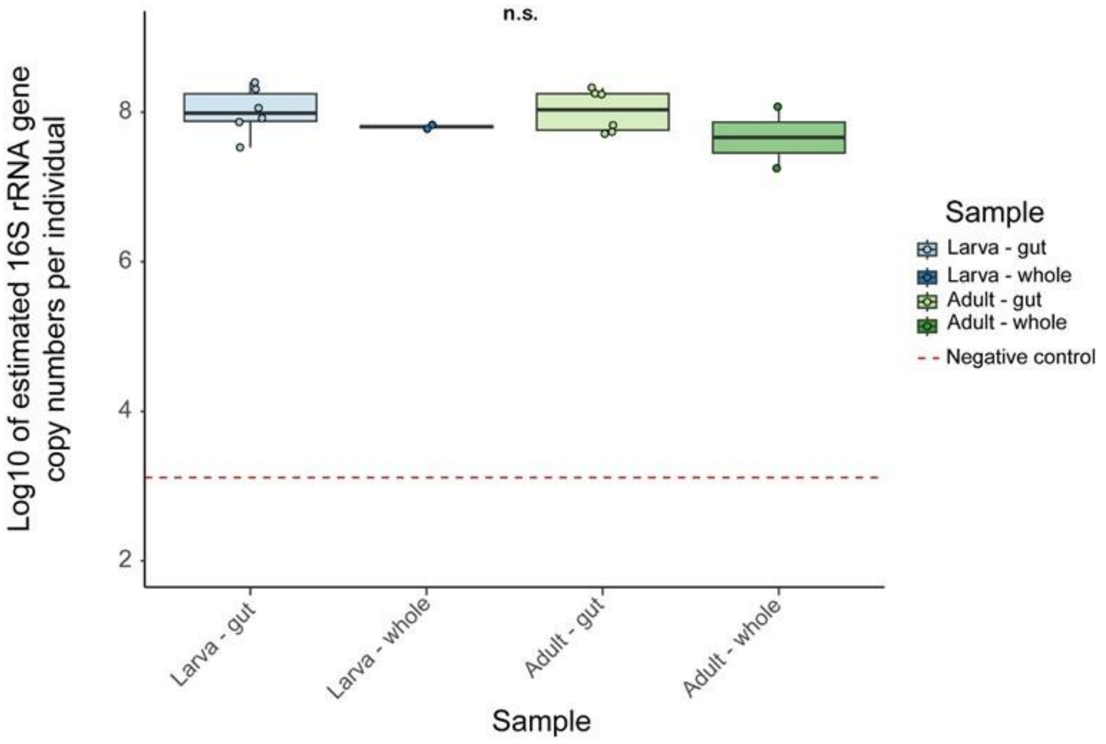

Figure S4.4 Bacterial loads in third-instar *Chrysomela tremulae* larvae and adult guts compared to whole individuals. There were no significant differences among the samples (negative binomial generalized linear model, Pr(>|z|) = 0.016). Millipore water was used instead of DNA extract for the negative control.

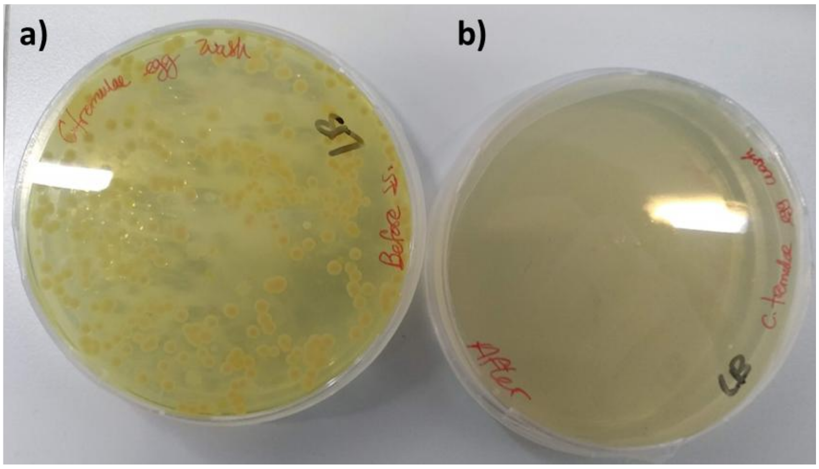

Figure S4.5 Bacterial colonies obtained from plating the PBS washing steps from the egg surface sterilization protocol 5 days post-inoculation, incubated at 28 °C. a) PBS wash before egg surface sterilization. b) after egg surface sterilization.

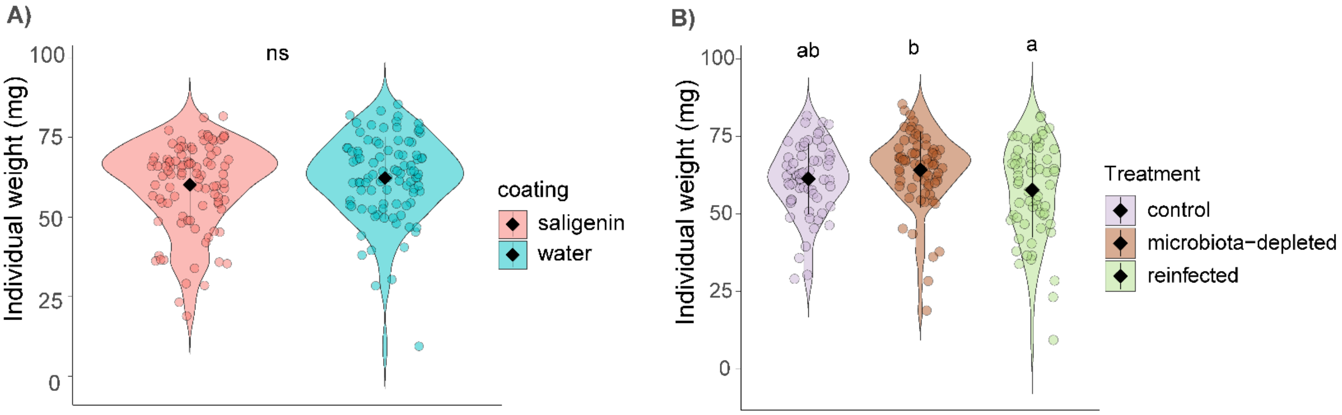

Figure S4.6 Individual *Chrysomela tremulae* larval weight at day 12 after hatching. A) Weight by leaf coating treatment. B) Weight by microbial community treatment. Statistical test: squared-root transformed data for normalization. Generalized linear model followed by least-squared means post-hoc test. Different letters indicate a P-value < 0.05 in the post-hoc test. n.s. = no significant differences, $P > 0.05$

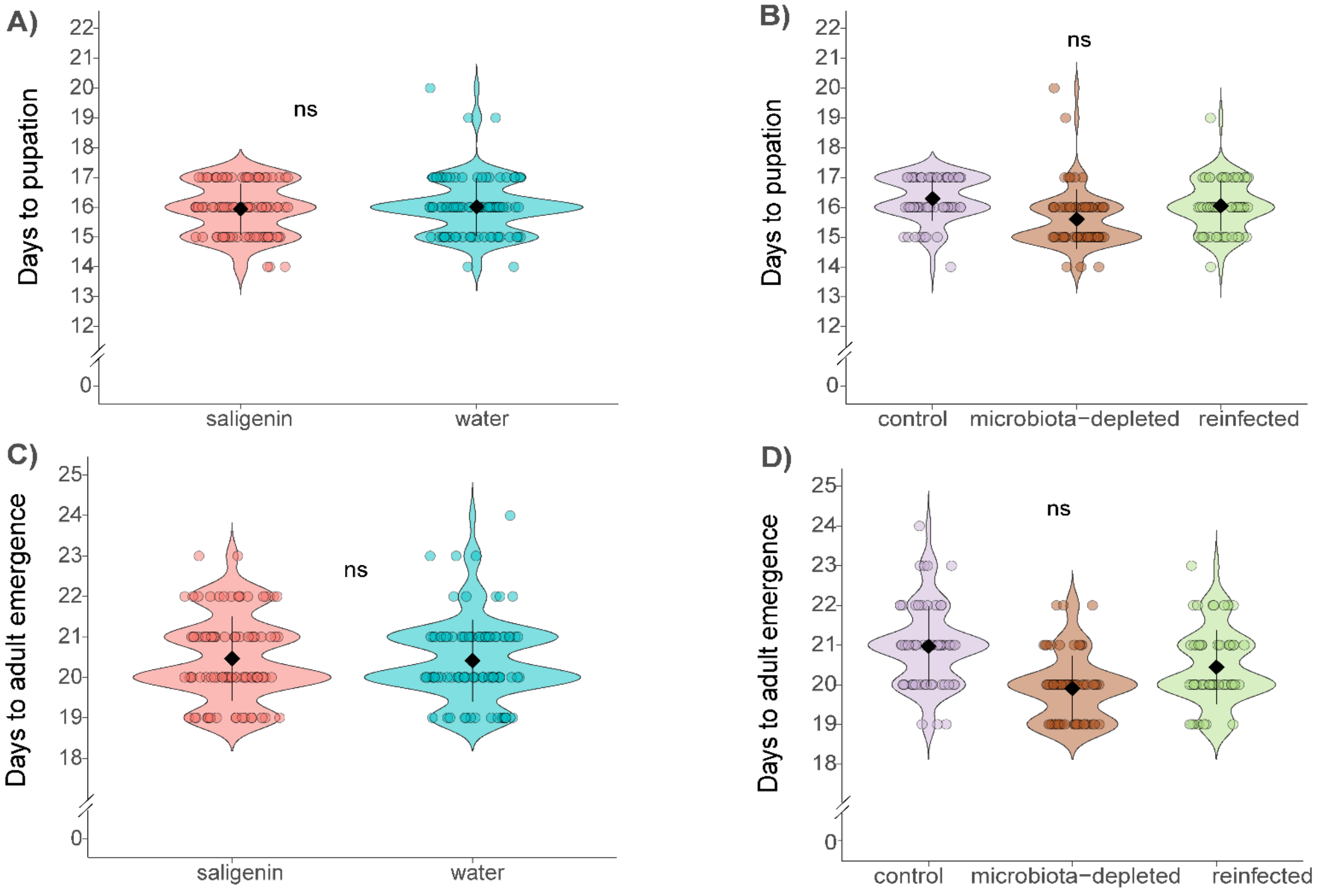

Figure S4.7 Development time of *Chrysomela tremulae* larvae exposed to a saligenin-enriched diet with the presence or absence of its native microbial community. A) Days to pupation of saligenin-rich versus water coating, B) days to pupation of microbiota-depleted, control and reinfected larvae, C) days until adult emergence of saligenin-rich versus water coating, D) days until adult emergence of microbiota-depleted, control, and reinfected larvae. Statistics: Poisson generalized linear model, post-hoc test: least-squared means. ns = no significant differences ($P > 0.05$).

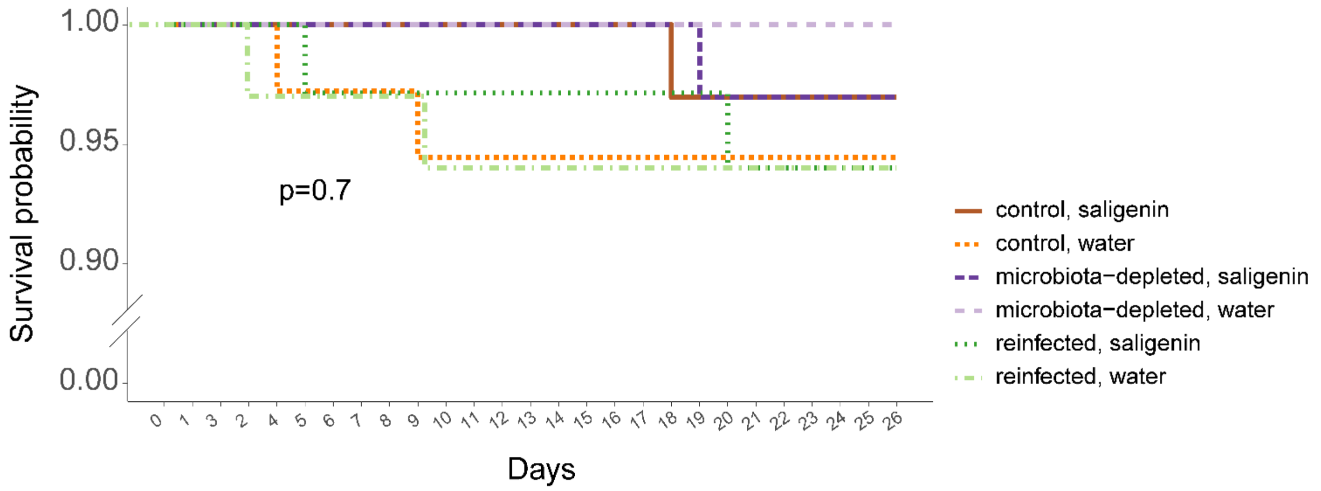

Figure S4.8 Kaplan-Meier estimate of survival probability of *Chrysomela tremulae* larvae assigned to different diets (saligenin-rich or water coating) and different microbial treatments (microbiota-depleted, control and reinfected).

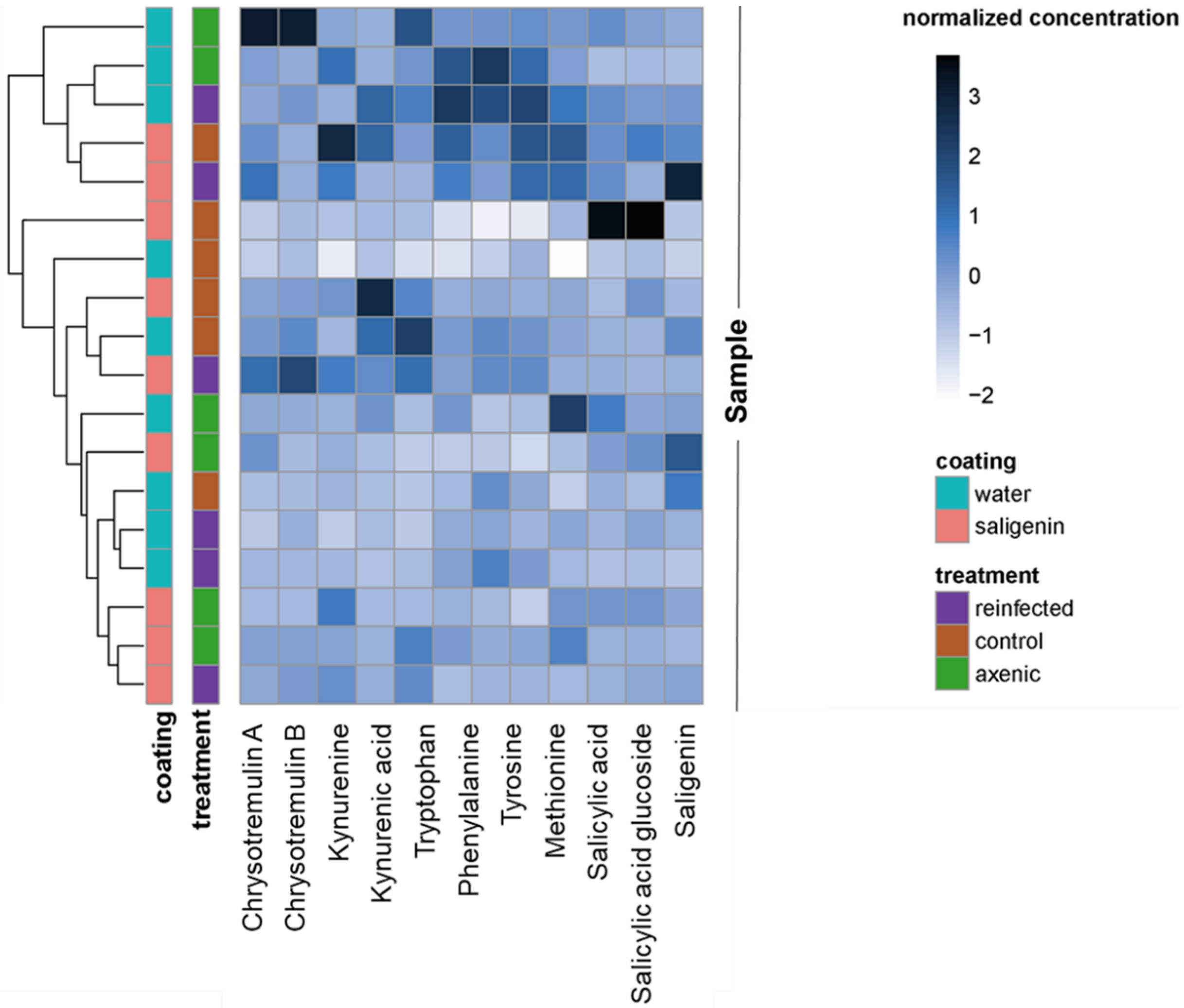

Figure S4.9 Heatmap of salicinoids and amino acids measured in third-instar *Chrysomela tremulae* larvae. Clustering method: Euclidean. Matrix normalization: by columns (compound concentrations).

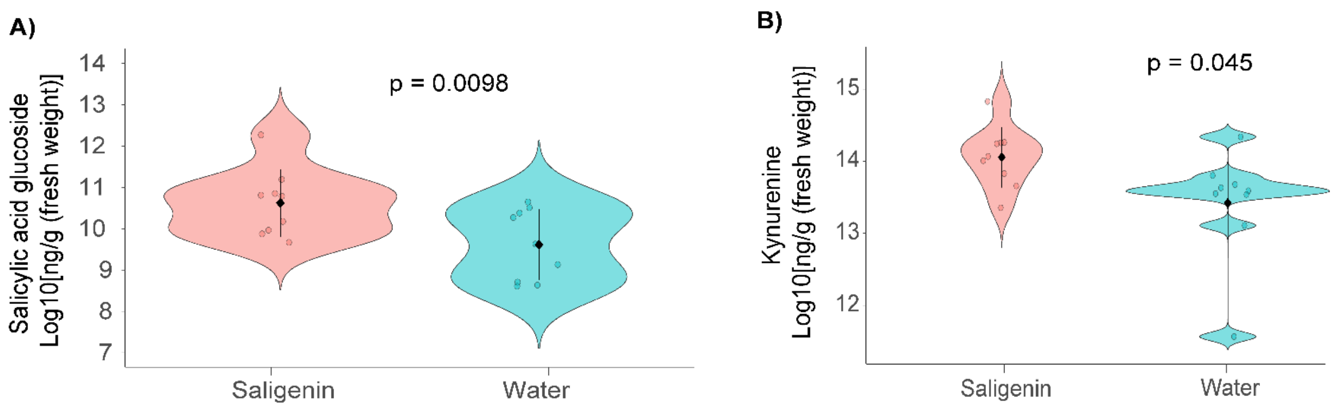

Figure S4.10 Compounds with significantly different concentrations in third-instar *Chrysomela tremulae* larvae exposed to increased saligenin contents in their diet. A) salicylic acid glucoside, B) kynurenine. Statistics: Generalized lognormal model followed by least-squared means post-hoc test.

Table S4.4 Statistical analysis of the compounds in third-instar *Chrysomela tremulae* larvae which were not significantly different between diet treatments nor among microbiota treatments. LSQM = least-squared means test.

**Compound**	**Model**	**Pr(>Chi)**	**LSQM**
Saligenin	glm(log(Salicylalcohol)~treat+coat)	treat: p = 0.55; coat: p = 0.26	n.s.
Chrysotremulin A (**11**)	glm(log(XP.1)~treat+coat)	treat: p = 0.73; coat: p = 0.21	n.s.
Chrysotremulin C (**13**)	glm(log(XP.3)~treat+coat)	treat: p = 0.34; coat: p = 0.97	n.s.
Tryptophan	glm(log(trp)~treat+coat)	treat: p = 0.39; coat: p = 0.33	n.s.
Salicylic acid	glm(log(SA)~treat+coat)	treat: p = 0.66; coat: p = 0.12	n.s.
Kynurenic acid	glm(log(kynurenic.acid)~treat+coat)	treat: p = 0.78; coat: p = 0.46	n.s
4-hydroxyquinoline	glm(log(X4.OH.quinoline)~treat+coat)	treat: p = 0.99; coat: p = 0.93	n.s.

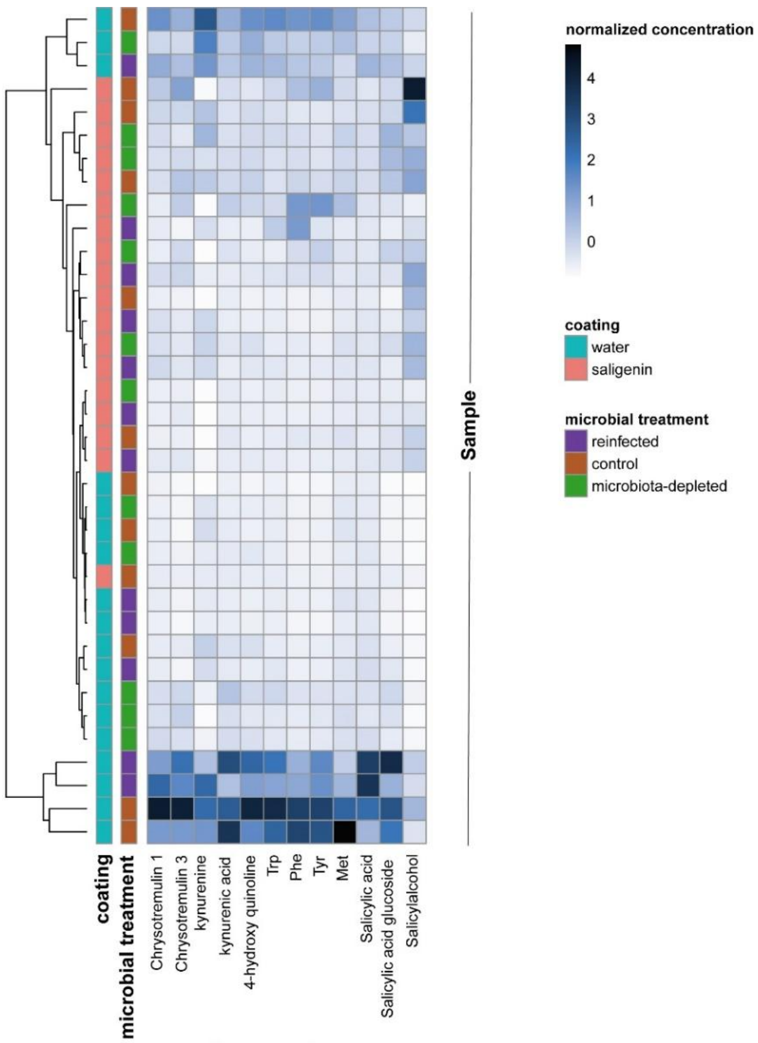

Figure S4.11 Heatmap of salicinoids and amino acids measured in the feces of *Chrysomela tremulae* larvae. Clustering method: Euclidean. Matrix normalization: by columns (compound concentrations).

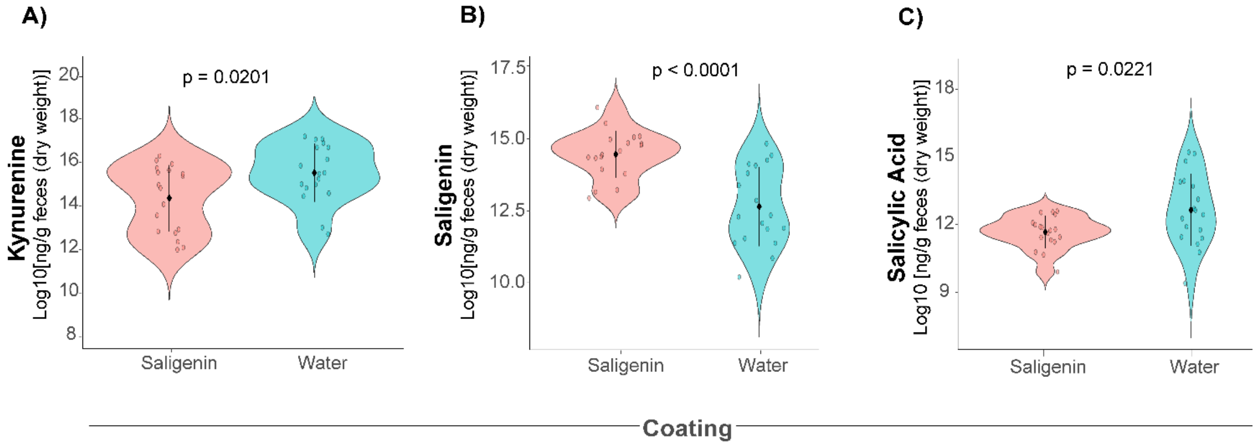

Figure S4.12 Compounds with significantly different concentrations in the feces of *Chrysomela*

tremulae larvae exposed to increased saligenin contents in their diet. A) kynurenine, B) saligenin, C) Salicylic Acid. Statistics: Generalized lognormal model followed by least-squared means post-hoc test.

Table S4.5 Statistical analysis of the remaining analyzed compounds in *Chrysomela tremulae* beetle feces which were not significantly different between diet treatments nor among microbiota treatments. LSQM = least-squared means test.

Compound	Model	Pr(>Chi)	LSQM
Chrysotremulin A (**11**)	glm(log(XP.1)~treat+coat+coat*treat	treat: p = 0.15; coat: p = 0.29	n.s.
Chrysotremulin C (**13**)	glm(log(XP.3)~treat+coat	treat: p = 0.92; coat: p = 0.97	n.s.
Tryptophan	glm(log(trp)~treat+coat	treat: p = 0.90; coat: p = 0.13	n.s.
Salicylic acid glucoside	glm(log(SA.Gluc)~treat+coat	treat: p = 0.92; coat: p = 0. 84	n.s.
Kynurenic acid	glm(log(kynurenic.acid)~treat+coat	treat: p = 0.44; coat: p = 0. 08	n.s
4-hydroxyquinoline	glm(log(X4.OH.quinoline)~treat+coat	treat: p = 0.92; coat: p = 0. 13	n.s.

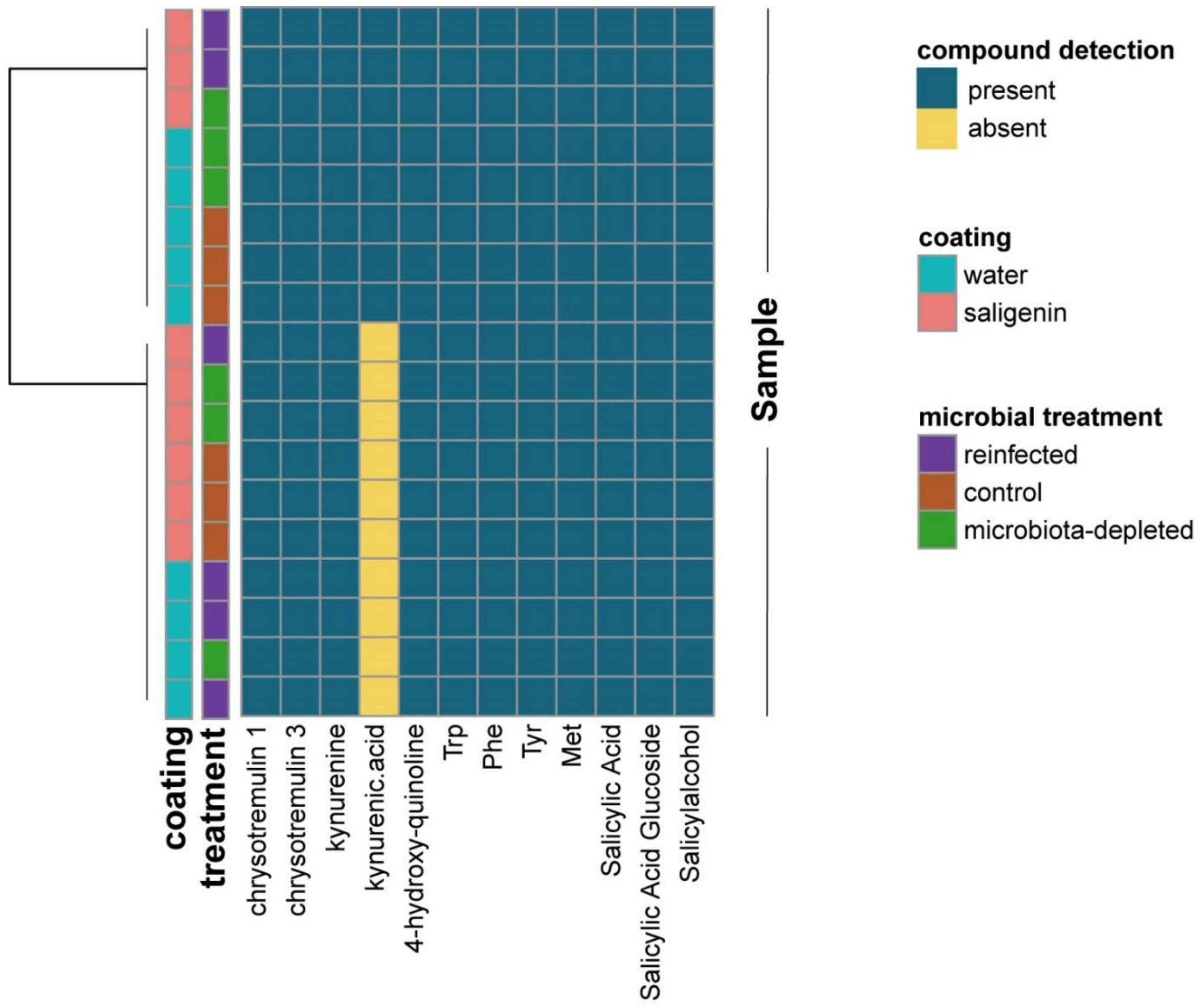

Figure S4.13 Presence/absence heatmap of salicinoids and amino acids measured in the defensive secretions of *Chrysomela tremulae* beetles. Clustering method: Euclidean; binary matrix.

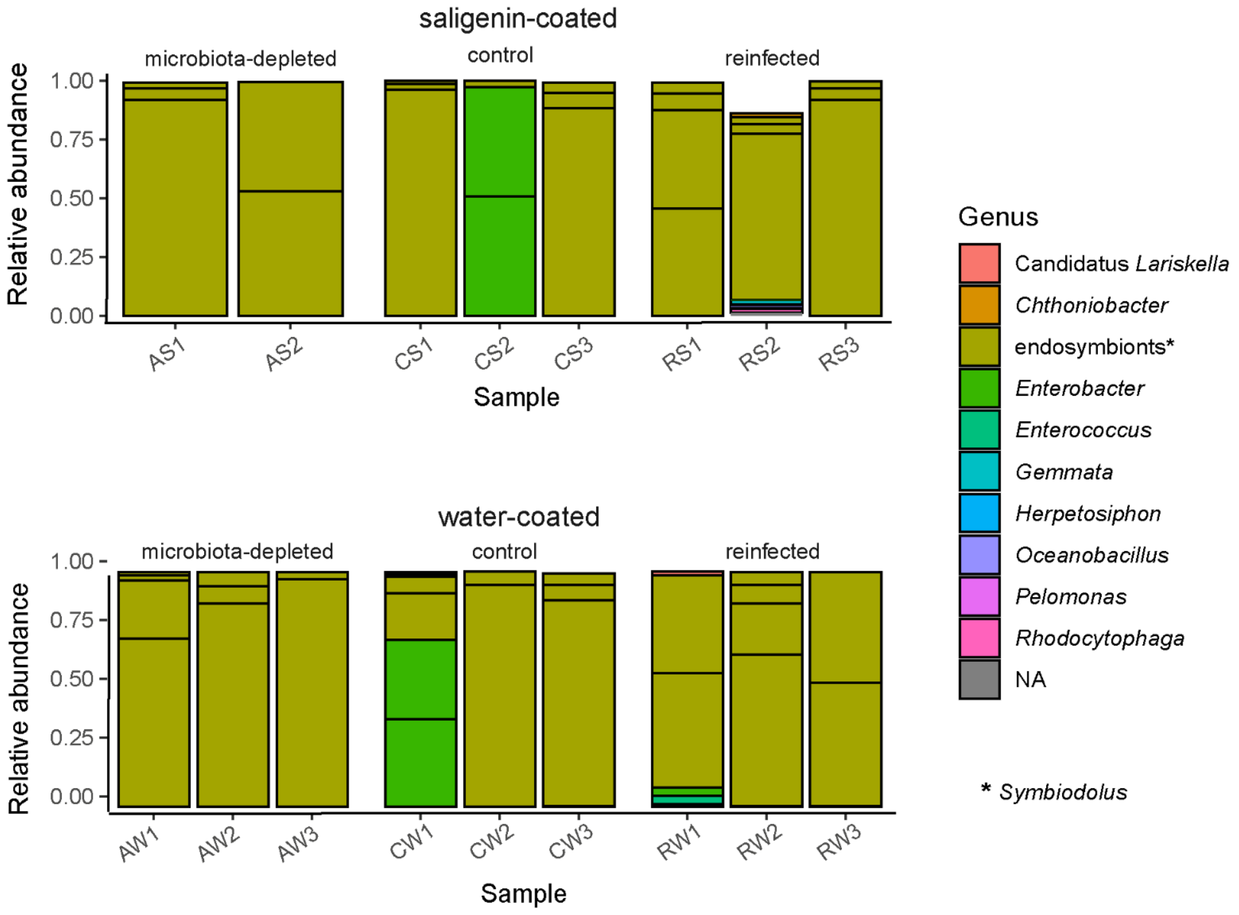

Figure S4.14 Relative abundances of the top 15 amplicon sequencing variants (ASVs) of *Chrysomela tremulae* larvae collected after 12 days of being subjected to increased saligenin contents in their diet. Relative abundances are indicated at ASV level; taxa marked with an asterisk (*) were refined to species by nucleotide blast against the NCBI database. When blasted against NCBI database, the assigned taxonomy corresponded to *Candidatus Symbiodolus clandestinus*. Top row: saligenin coating. Bottom row: water coating control. Columns from left to right: microbiota-depleted, control, reinfected larvae.

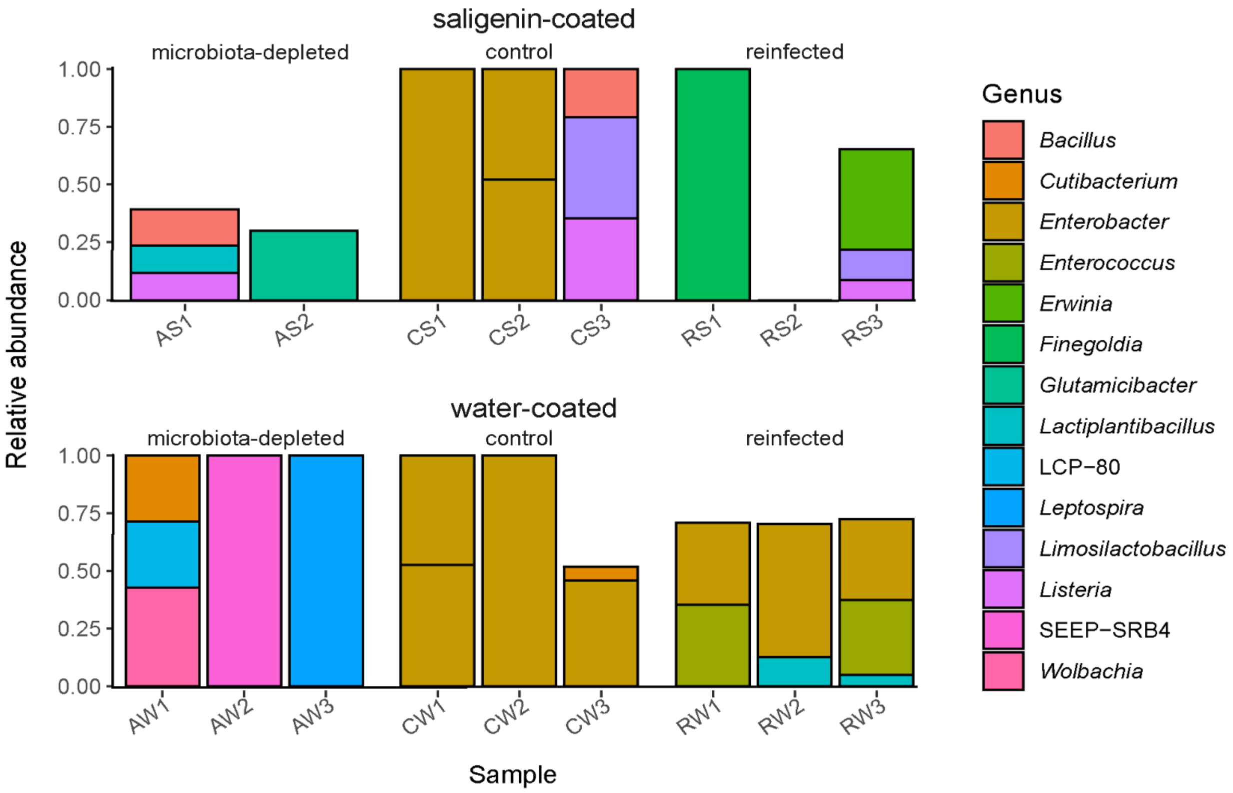

Figure S4.15 Relative abundances of the top 15 amplicon sequencing variants (ASVs) excluding the "endosymbiont" (*Symbiodolus*) ASV. *Chrysomela tremulae* larvae collected after 12 days of being subjected to increased saligenin contents in their diet. Top row: saligenin coating. Bottom row: water coating control. Columns from left to right: microbiota-depleted, control, reinfected larvae.

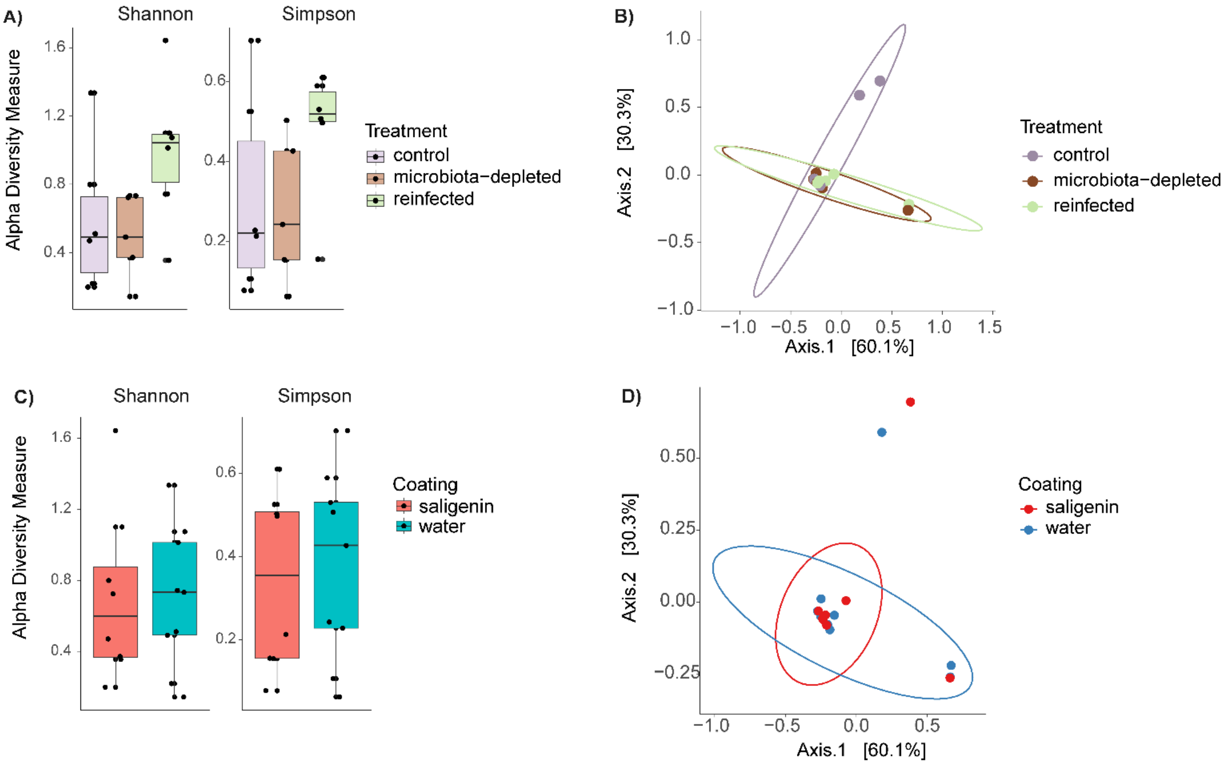

Figure S4.16 Diversity of the bacterial communities in larvae of *Chrysomela tremulae* after 12 days of initiating the saligenin-microbiota assay. A) α-diversity indices of the bacterial communities of microbiota-depleted, control and reinfected larvae, B) Principal Coordinate Analysis (PCoA) of Bray Curtis dissimilarity matrix depicting the β-diversity of the bacterial communities of microbiota-depleted, control and reinfected larvae, C) α-diversity indices of the bacterial communities of larvae assigned to saligenin or water coating diet treatments, D) Principal Coordinate Analysis (PCoA) of Bray Curtis dissimilarity matrix depicting the β-diversity of the bacterial communities of larvae assigned to saligenin or water coating diet treatments.

References:

1. T. Fabisch, J. Gershenzon, S. B. Unsicker, Specificity of herbivore defense responses in a woody plant, black poplar (*Populus nigra*). *J. Chem. Ecol.* **45**, 162-177 (2019).
2. F. Feistel, C. Paetz, R. C. Menezes, D. Veit, B. Schneider, Acylated quinic acids are the main salicortin metabolites in the Lepidopteran specialist herbivore *Cerura vinula*. *J. Chem. Ecol.* **44**, 497-509 (2018).
3. S. Abusoglu, D. Eryavuz Onmaz, G. Abusoglu, F. Humeyra Yerlikaya, A. Unlu, Measurement of kynurenine pathway metabolites by tandem mass spectrometry. *J. Mass Spectrom Adv. Clin. Lab.* **28**, 114-121 (2023).
4. X. R. Peng *et al.*, (+/-)-Spiroganoapplanin A, a complex polycyclic meroterpenoid dimer from *Ganoderma applanatum* displaying potential against Alzheimer's disease. *Org Chem Front* **9**, 3093-3101 (2022).
5. J. Wang *et al.*, Resveratrol alleviating the ovarian function under oxidative stress by alternating microbiota related tryptophan-kynurenine pathway. *Front. Immunol.* **13**, 911381-911398 (2022).
6. Z. Zhang *et al.*, Honeybee gut *Lactobacillus* modulates host learning and memory behaviors via regulating tryptophan metabolism. *Nat. Commun.* **13**, 2037-2050 (2022).
7. D. A. Rodriguez-Soacha *et al.*, "Photo-rimonabant": synthesis and biological evaluation of novel photoswitchable molecules derived from rimonabant lead to a highly selective and nanomolar "Cis-On" CB(1)R antagonist. *ACS Chem. Neurosci.* **12**, 1632-1647 (2021).
8. Z. Tang, W. Chen, Z. Zhu, H. Liu, SnCl4-catalyzed aza-acetalization of aromatic aldehydes: synthesis of aryl substituted 3,4-dihydro-2H-1,3-benzoxazines. *Syn. Commun.* **42**, 1372-1383 (2012).
9. C. M. Adeyemi *et al.*, Synthesis and anti-parasitic activity of C -benzylated (N -arylcarbamoyl)alkylphosphonate esters. *Tetrahedron* **73**, 1661-1667 (2017).
10. J. A. Ceja-Navarro, E. L. Brodie, F. E. Vega, A technique to dissect the alimentary canal of the coffee berry borer (*Hypothenemus hampei*), with isolation of internal microorganisms. *J. Entomol. Acarol. Res.* **44**, 21-24 (2012).
11. A. Klindworth *et al.*, Evaluation of general 16S ribosomal RNA gene PCR primers for classical and next-generation sequencing-based diversity studies. *Nucleic Acids Res.* **41**, e1-e11 (2013).

12. B. J. Callahan *et al.*, DADA2: High-resolution sample inference from Illumina amplicon data. *Nat. Methods* **13**, 581-583 (2016).
13. C. Quast *et al.*, The SILVA ribosomal RNA gene database project: improved data processing and web-based tools. *Nucleic Acids Res.* **41**, D590-D596 (2012).
14. M. Watson, P. J. McMurdie, S. Holmes, phyloseq: An R package for reproducible interactive analysis and graphics of microbiome census data. *PLoS One* **8**, e61217-e61228 (2013).
15. A. C. McHardy, P. J. McMurdie, S. Holmes, Waste not, want not: why rarefying microbiome data is inadmissible. *PLoS Comput. Biol.* **10**, e1003531-e1003543 (2014).
16. N. Fierer, J. A. Jackson, R. Vilgalys, R. B. Jackson, Assessment of soil microbial community structure by use of taxon-specific quantitative PCR assays. *Appl. Environ. Microbiol.* **71**, 4117-4120 (2005).
